# SOX17 is a Critical Factor in Maintaining Endothelial Function in Pulmonary Hypertension by an Exosome‐Mediated Autocrine Manner

**DOI:** 10.1002/advs.202206139

**Published:** 2023-03-15

**Authors:** Xiaozhou Zou, Ting Liu, Zhongjie Huang, Wei Zhou, Mengnan Yuan, Hongying Zhao, Zongfu Pan, Pengcheng Chen, Yanfei Shao, Xiaoping Hu, Su Zhang, Shuilian Zheng, Yiwen Zhang, Ping Huang

**Affiliations:** ^1^ Center for Clinical Pharmacy Cancer Center Department of Pharmacy Zhejiang Provincial People's Hospital Affiliated People's Hospital Hangzhou Medical College Hangzhou 310014 P. R. China; ^2^ Key Laboratory of Endocrine Gland Diseases of Zhejiang Province Hangzhou 310014 P. R. China; ^3^ Department of Pharmacy Affiliated Hangzhou First People's Hospital Zhejiang University School of Medicine Hangzhou 310006 P. R. China; ^4^ Department of Clinical Pharmacy Key Laboratory of Clinical Cancer Pharmacology and Toxicology Research of Zhejiang Province Affiliated Hangzhou First People's Hospital Zhejiang University School of Medicine Hangzhou 310006 P. R. China; ^5^ School of Pharmaceutical Sciences Zhejiang Chinese Medical University Hangzhou 310014 P. R. China; ^6^ Zhongnan Hospital of Wuhan University Institute of Hepatobiliary Diseases of Wuhan University Transplant Center of Wuhan University Hubei Key Laboratory of Medical Technology on Transplantation Wuhan 430000 P. R. China

**Keywords:** endothelium dysfunction, exosomes, miRNAs, pulmonary hypertension, sry‐related high mobility group domain family F 17

## Abstract

Endothelial dysfunction is considered a predominant driver for pulmonary vascular remodeling in pulmonary hypertension (PH). SOX17, a key regulator of vascular homoeostasis, has been found to harbor mutations in PH patients, which are associated with PH susceptibility. Here, this study explores whether SOX17 mediates the autocrine activity of pulmonary artery ECs to maintain endothelial function and vascular homeostasis in PH and its underlying mechanism. It is found that SOX17 expression is downregulated in the endothelium of remodeled pulmonary arteries in IPH patients and SU5416/hypoxia (Su/hypo)‐induced PH mice as well as dysfunctional HPAECs. Endothelial knockdown of SOX17 accelerates the progression of Su/hypo‐induced PH in mice. SOX17 overexpression in the pulmonary endothelium of mice attenuates Su/hypo‐induced PH. SOX17‐associated exosomes block the proliferation, apoptosis, and inflammation of HPAECs, preventing pulmonary arterial remodeling and Su/hypo‐induced PH. Mechanistic analyses demonstrates that overexpressing SOX17 promotes the exosome‐mediated release of miR‐224‐5p and miR‐361‐3p, which are internalized by injured HPAECs in an autocrine manner, ultimately repressing the upregulation of NR4A3 and PCSK9 genes and improving endothelial function. These results suggest that SOX17 is a key gene in maintaining endothelial function and vascular homeostasis in PH through regulating exosomal miRNAs in an autocrine manner.

## Graphical abstract

Endothelial dysfunction is considered a predominant driver for pulmonary vascular remodeling in pulmonary hypertension. In this study, it is shown that SOX17, serving as a key factor in maintaining endothelial function and vascular homeostasis, prevents endothelial dysfunction and pulmonary arterial remodeling through regulating exosomal miRNAs in an autocrine manner in pulmonary hypertension.

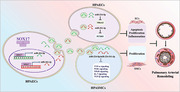

## 1 Introduction

Pulmonary arterial hypertension (PH) is a clinical and pathophysiological syndrome characterized by pulmonary vascular resistance and elevated pulmonary arterial pressure. Vascular remodeling is the main pathological mechanism underlying stenosis and occlusion of the pulmonary arteries in PH, accompanied by endothelial dysfunction and activation of fibroblasts and smooth muscle cells (SMCs).^[^ ^1^ ^]^ Endothelial cells (ECs) are the first critical vascular barrier to maintain vascular homeostasis via preserving vascular integrity, retaining vascular tone, and exhibiting an anti‐inflammatory niche. During the development of PH, endothelial dysfunction is believed to be the initial event of pulmonary vascular remodeling.^[^ ^2^ ^]^

The vascular endothelium is a tissue with powerful secretory functions and modulate vascular homeostasis by secreting various cytokines, such as endothelin 1, prostacyclin, and VEGF, in an autocrine or paracrine manner.^[^ ^3^ ^]^ Growing evidence supports that exosomes are also excellent regulators of vascular homeostasis via delivering multiple factors, such as DNA, RNA, lipids, metabolites, and cytosolic or cell surface proteins, to adjacent as well as distal vascular cells.^[^ ^4^ ^]^ In PH, exosomes derived from pulmonary arterial SMCs (PASMCs) mediate cell‐to‐cell communication between PASMCs and pulmonary arterial ECs (PAECs) to cause EC migration and vascular remodeling.^[^ ^5^ ^]^ Exosomes secreted from KLF2‐expressing ECs block atherosclerotic lesion formation by transporting miR‐143/145 to VSMCs in the aorta of ApoE^(−/−)^ mice.^[^ ^6^ ^]^ SMC‐derived exosomes mediate the transfer of KLF5‐induced miR‐155 from SMCs to ECs in a paracrine manner, destroying tight junctions and leading to an increased endothelial permeability and enhanced atherosclerotic progression.^[^ ^7^ ^]^ However, it is unclear whether PAEC‐derived exosomes participate in the regulation of PAEC function in an autocrine manner in pulmonary vascular homeostasis during PH.

Sry‐related high mobility group domain family F 17 (SOX17), a member of the Sry‐related high mobility group domain family of transcription factors, is a crucial developmental regulator of endothelial and hematopoietic lineages.^[^ ^8^, ^9^ ^]^ In the embryonic pulmonary vasculature, SOX17 is mainly expressed in mesenchymal progenitors, but is restricted to vascular endothelial cells in the mature lung.^[^ ^10^ ^]^ Endothelial‐specific upregulation of SOX17 enhances lung endothelial regeneration, ultimately restoring vascular homeostasis in the endotoxemia model.^[^ ^11^ ^]^ Sox17 expression in mouse embryonic and adult arterial ECs is also an important protective mechanism of arterial integrity, and SOX17 may be a key regulator of pulmonary vascular homoeostasis. Recently, SOX17 was reported as a novel PH candidate gene from several whole genome and whole exome sequencing analyses, with rare loss‐of‐function variants identified in heritable PH (HPH) patients.^[^ ^12^, ^13^, ^14^ ^]^ A heterozygous SOX17 mutation, NM_022454.4: c.379C>T; p. (Gln127*), is also found in human PAECs (HPAECs) to co‐segregate with the disease in the family, with complete penetrance.^[^ ^15^ ^]^ In our preliminary study, SOX17 was found to be decreased in the endothelium of remodeling pulmonary arteries in idiopathic PH (IPH) patients, accompanied by obvious proliferation and inflammation. Thus, SOX17 loss may be a generalizable condition not unique to HPH, which may be closely related to endothelial dysfunction and vascular dyshomeostasis in IPH and other forms of PH.

The current study aimed to investigate if SOX17 mediates the autocrine regulation of PAECs to maintain endothelial function and pulmonary vascular homeostasis in PH and its underlying mechanism.

## 2 Results

### 2.1 Endothelial‐Specific SOX17 Knockdown Accelerated the Progression of PH and SOX17 Overexpression Prevented PH through Mediating the Autocrine Action of PAECs in Regulating Endothelial Function

**Figure 1:**
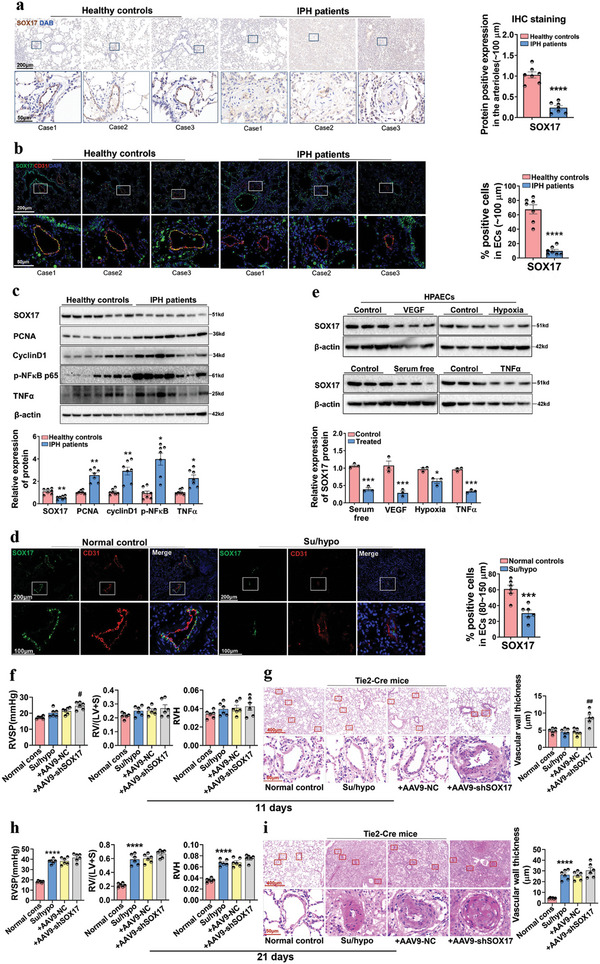
The expression of SOX17 is absent in the pulmonary arterial endothelium from IPH patients and in HPAECs treated by serum‐free medium, VEGF, hypoxia, and TNF*α*. a) IHC staining for the expression of SOX17 in the lung tissues of healthy donors and IPH patients, and the quantification of SOX17^+^ pulmonary arteries (≈100 µm). Values are mean fold‐changes compared to healthy controls, ****P < 0.0001 versus healthy controls, *n* = 7. b) Dual‐IF staining for the location and expression of SOX17 and CD31 in the lung tissues of healthy donors and IPH patients, and the quantification of SOX17^+^ and CD31^+^ pulmonary arteries (≈100 µm). Values are mean fold‐changes of healthy controls, ****P < 0.0001 versus healthy controls, *n* = 7. c) Western blotting assay for the expression of SOX17, PCNA, cyclinD1, p‐NF*κ*B, and TNF*α* proteins in the lung homogenates from healthy controls and IPH patients, and the quantified data, *P < 0.05, **P < 0.01, ***P < 0.001 versus healthy controls, *n* = 7. d) Dual‐IF staining for the location and expression of SOX17 and CD31 in the lung tissues of mice treated with Su/hypo, ***P < 0.001 versus normal controls, *n* = 6. e) Western blotting assay for the protein expression of SOX17 in HPAECs treated with VEGF, serum‐free (HPAECs were seeded and allowed to attach for 24 h in the normal medium [ECM endothelial cells medium, supplemented with 5% FBS, 1% endothelial cell growth supplement, and 1% antibiotic solution]). The following day, the medium was removed, and the cells were cultured in serum‐free medium (ECM endothelial cells medium, supplemented with 0% FBS, 1% endothelial cell growth supplement, and 1% antibiotic solution), hypoxia, and TNF*α*, and the quantified data, *P < 0.05, ***P < 0.001 versus Control, *n* = 3. f) RVSP, right ventricular hypertrophy (RV/LV+S), RV weight/tibial length (RVH) in the Tie2‐Cre mice treated with AAV9‐shSOX17‐loxp (AAV9‐shSOX17) for 21 d and Su/hypo for another 11 d were examined, #*P* < 0.05 versus Su/hypo + AAV9‐NC‐loxp (AAV9‐NC), *n* = 6. g) HE staining for the vascular remodeling in the Tie2‐Cre mice, and the quantification of vascular wall thickness (80–150 µm), ##*P* < 0.01 versus Su/hypo + AAV9‐NC, *n* = 6. h) RVSP, RV/LV+S, RVH in the Tie2‐Cre mice treated with AAV9‐shSOX17 for 21 d and Su/hypo for another 21 d were examined, ****P < 0.0001 versus Normal controls, *n* = 6. i) HE staining for the vascular remodeling in the Tie2‐Cre mice, and the quantification of vascular wall thickness (80–150 µm), ****P < 0. 0001 versus Normal controls, *n* = 6.

The expression of SOX17 was examined in the pulmonary arteries of patients with IPH and healthy controls (*n* = 7). All investigations using human samples were conducted as per the guidelines specified in the Declaration of Helsinki and approved by the Ethics Committee of Zhong Nan Hospital of Wuhan University (IRB‐2021077k). As shown in **Figure** **1**a, SOX17 was present in the pulmonary arteries of healthy controls but decreased in the remodeled pulmonary arteries of IPH patients. The dual‐fluorescence (dual‐IF) assay for CD31 and SOX17 proteins verified the location and specific downregulation of SOX17 in the endothelium of remodeled pulmonary arteries from IPH patients (Figure 1b). Western blotting of the pulmonary tissue homogenates also revealed a decrease of SOX17 in IPH patients, accompanied by an increase of PCNA, cyclin D1, p‐NF‐*κ*B p65, and TNF*α* (Figure 1c). The expression of SOX17 in the endothelium of SU5416/hypoxia (Su/hypo)‐induced PH mice was detected. Dual‐IF assay for CD31 and SOX17 confirmed the location and specific downregulation of SOX17 in the endothelium of remodeled pulmonary arteries from PH mice (Figure 1d). We further detected the expression of SOX17 in HPAECs under multiple conditions that induce endothelial dysfunction. As shown in Figure 1e, the expression of SOX17 in HPAECs was inhibited by VEGF, serum free, hypoxia, and TNF*α*. Then, the role of SOX17 knockdown in HPAEC dysfunction was detected. As shown in Figure S1a,b (Supporting Information), SOX17 siRNA induced a significant upregulation of PCNA, cyclinD1, p‐NF*κ*B p65, without affecting the expression of caspase3 and cleaved‐caspase3. To detect the role of SOX17 in the endothelium dysfunction and vascular remodeling of PH, we built endothelial‐specific SOX17‐knockdown and ‐overexpression mice by injecting Tie2‐Cre mice with AAV9‐shSOX17‐loxp (AAV9‐shSOX17) and AAV9‐SOX17‐loxp (AAV9‐SOX17) through a single intratracheal injection. AAV9‐shSOX17 treatment for 21 days decreased the expression of SOX17 in the PAECs separated from the Tie2‐Cre mice and the pulmonary arterial endothelium of the Tie2‐Cre mice (Figure S1c,d, Supporting Information), without affecting the expression of SOX17 in the PASMCs separated from the Tie2‐Cre mice (Figure S1e, Supporting Information). Furthermore, SOX17 knockdown alone in the pulmonary endothelium of mice did not affect the pulmonary vascular structure and hemodynamics index about PH (Figure S1f,g, Supporting Information). Then, the Tie2‐Cre mice with endothelial‐specific SOX17 knockdown were treated with Su/hypo for 21 d. As shown in Figure 1f,g, Su/hypo treatment for 11 d increased right ventricular systolic pressure (RVSP) and caused vascular remodeling in the SOX17‐knockdown Tie2‐Cre mice, without affecting the normal Tie2‐Cre mice. However, at day 21 post‐Su/hypo, there was no appreciable difference between the SOX17‐knockdown and normal Tie2‐Cre mice (Figure 1h,i). These findings indicated that endothelial‐specific SOX17 knockdown accelerated the development of PH. Furthermore, we also found endothelial‐specific SOX17 overexpression could attenuate Su/hypo‐induced PH (Figure S2, Supporting Information).

We then explored whether PAECs could regulate endothelial function in an autocrine manner and whether SOX17 mediated this process. First, HPAECs (at 1 × 10^5^ cells mL^−1^ cellular density) treated with VEGF, TNF*α*, or serum‐free medium were co‐cultured with normal HPAECs at three cellular densities (1 × 10^4^, 1 × 10^5^, and 1 × 10^6^ cells mL^−1^), respectively, in transwell dishes and separated by a porous membrane, which allows exchange of exosomal particles^[^ ^16^ ^]^ (Figure S3a, Supporting Information). HPAECs at high density suppressed VEGF‐induced proliferation, TNF*α*‐caused inflammation, and serum‐free‐induced apoptosis (Figure S3b–d, Supporting Information). Next, SOX17 was overexpressed by an adenoviral gene vector (Ad‐SOX17) (Figure S4a,b, Supporting Information). SOX17‐overexpressing HPAECs at 1 × 10^5^ cells mL^−1^ were co‐cultured with HPAECs (1 × 10^5^ cells mL^−1^) treated with VEGF, serum‐free medium, hypoxia, and TNF*α* (Figure S5a, Supporting Information). Co‐culture did not affect the expression of SOX17 in the lower chamber (Figure S5b,c, Supporting Information), but blocked VEGF‐induced proliferation (Figure S5d,e, Supporting Information), serum‐free‐induced apoptosis (Figure S5f,g, Supporting Information), and hypoxia‐ and TNF*α*‐induced inflammation of HPAECs (Figure S5h,i, Supporting Information). These results indicated that SOX17 mediates the autocrine regulation of PAECs on endothelial function, possibly through exosomes.

### 2.2 SOX17‐Associated Exosomes Mediate the Autocrine Regulation of PAECs on Endothelial Function and Attenuate Pulmonary Arterial Remodeling and PH

To confirm the role of SOX17‐associated exosomes on endothelial function, the supernatant of SOX17‐overexpressing HPAECs was used to isolate SOX17‐associated exosomes. The purified exosomes were identified using transmission electron microscopy (TEM) (Figure S6a, Supporting Information) and protein biomarker analysis (Figure S6b, Supporting Information). The size of the exosomes in the conditioned media was predominantly <100 nm in diameter, and 1 mL medium contained ≈1–2 × 10^10^ particles (Figure S6c, Supporting Information). We further examined whether exosomes might deliver virions. As shown in Figure S7a (Supporting Information), there was no GFP fluorescence signal in HPAECs incubated with SOX17‐associated exosomes. The virions were primarily distributed in the nucleus of HPAECs after transfection with Ad‐Empty for 12 h, which were mostly excluded from cytoplasmic vesicles (Figure S7b, Supporting Information), indicating virions were not encapsulated by exosomes.

**Figure 2:**
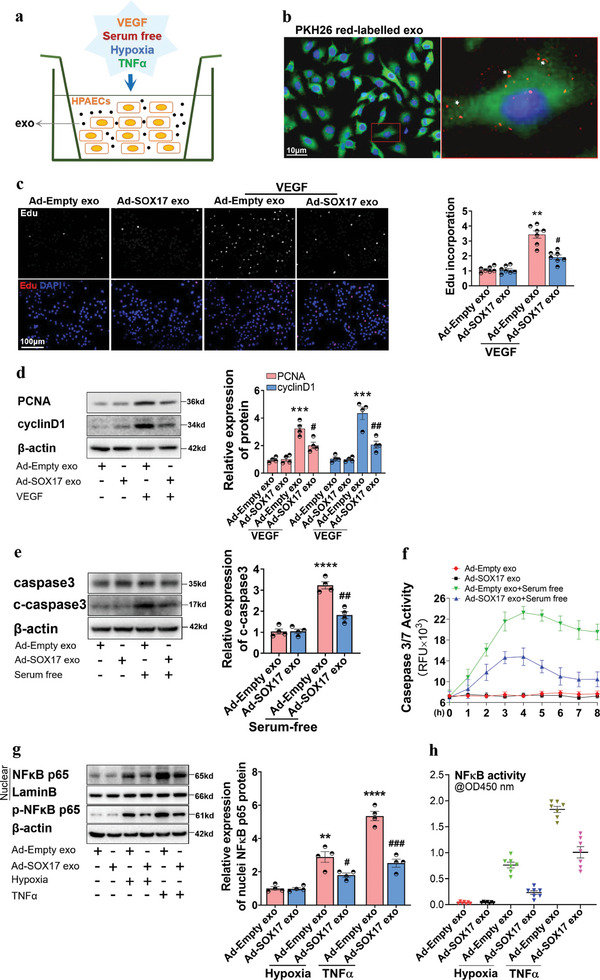
SOX17 overexpression in HPAECs resisted endothelial dysfunction through exosomes. a) The schematic diagram for the conditional treatment of HPAECs. b) The internalization of exosomes labeled with PKH26 was detected by the red stains, as indicated by white arrowheads. c) The DNA replication activity (EdU assay) for the proliferation induced by VEGF in HPAECs co‐cultured with SOX17‐associated exosomes, and the quantified data, **P < 0.01 versus Ad‐Empty exo, #*P* < 0.05 versus Ad‐Empty exo + VEGF group, *n* = 7. d) Western blotting assay for the protein expression of PCNA and cyclin D1 induced by VEGF in HPAECs co‐cultured with SOX17‐associated exosomes, and the quantified data, ***P < 0.001 versus Ad‐Empty exo group, #*P* < 0.05, ##*P* < 0.01 versus Ad‐Empty exo + VEGF group, *n* = 4. e) Western blotting assay for the expression of caspase3 and cleaved‐caspase3 proteins induced by serum‐free conditions in HPAECs treated with SOX17‐associated exosomes, and the quantified data, ****P < 0.0001 versus Ad‐Empty exo group, ##*P* < 0.01 versus Ad‐Empty exo + serum‐free group, *n* = 4. f) Caspase 3/7 activation induced by serum‐free conditions in HPAECs treated with SOX17‐associated exosomes were examined by the Apo‐One Homogeneous caspase3/7 assay kits, *n* = 6. g) Western blotting assay for the protein expression of nuclear NF*κ*B p65 and p‐NF*κ*B p65 stimulated by hypoxia and TNF‐*α* in HPAECs treated with SOX17‐associated exosomes, **P < 0.01, ****P < 0.0001 versus Ad‐Empty exo group, #*P* < 0.05, ###*P* < 0.001 versus treatment controls, *n* = 4. h) NF*κ*B activation stimulated by hypoxia and TNF‐*α* in HPAECs treated with SOX17‐associated exosomes was examined by NF*κ*B p65 transcription factor assay kits, *n* = 7.

HPAECs were then cultured with SOX17‐associated exosomes under the treatment of VEGF, serum‐free conditions, hypoxia, and TNF*α* to explore the role of SOX17‐associated exosomes on endothelial function (**Figure** **2**a). To detect the internalization of exosomes by HPAECs, SOX17‐associated exosomes were labeled with PKH‐26 and incubated with normal HPAECs. As revealed in Figure 2b, red fluorescence was found in the cytoplasm of HPAECs. In addition, the expression of SOX17 in HPAECs incubated with SOX17‐associated exosomes was not changed at either the protein or mRNA levels (Figure S8a, Supporting Information). The number of exosomes did not differ significantly between the treated groups (Figure S8b, Supporting Information), excluding the effect of SOX17 on the number of exosomes. After incubation with SOX17‐associated exosomes, VEGF‐induced proliferation (Figure 2c,d), serum‐free‐induced apoptosis (Figure 2e,f), and hypoxia‐ and TNF *α*‐caused inflammation (Figure 2g,h and Figure S9a, Supporting Information) in HPAECs were significantly suppressed. These results demonstrated that SOX17‐associated exosomes mediate the autocrine regulation of PAECs on endothelial function.

**Figure 3:**
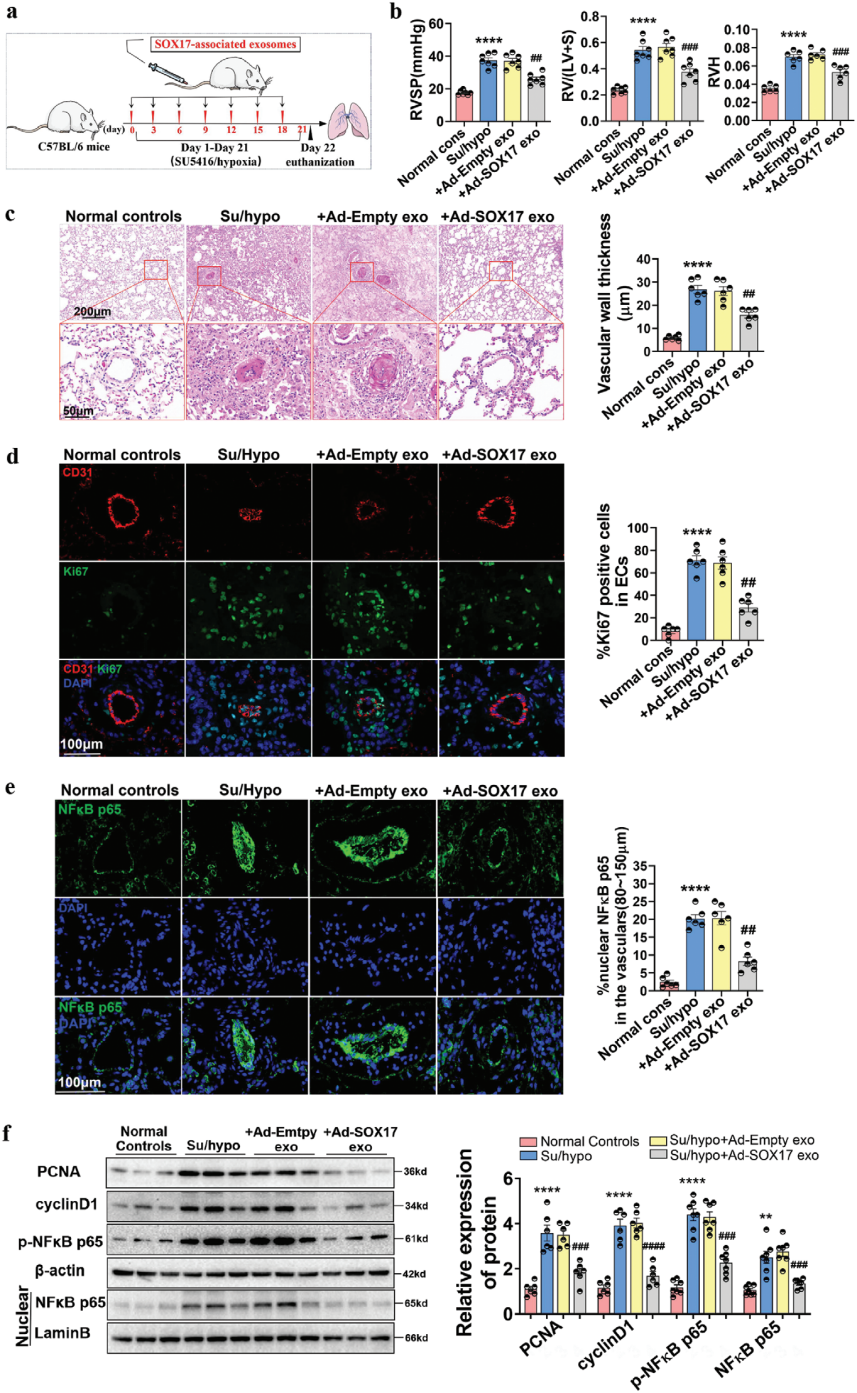
SOX17‐associated exosomes blocked vascular remodeling, proliferation, and inflammation in mice with Su/hypo‐induced PH. a) The schematic diagram for the construction of PH mice model. b) RVSP, RV/LV+S, and RVH in normal mice, PH mice, and PH mice treated with SOX17‐associated exosomes were examined, ****P < 0.0001 versus Normal controls, ##*P* < 0.01, ###*P* < 0.001 versus Su/hypo + Ad‐Empty exo, *n* = 6–7. c) HE staining of the lung tissues of healthy mice, PH mice, and PH mice treated with SOX17‐associated exosomes, with quantification of vascular wall thickness (80–150 µm), ****P < 0.0001 versus Normal controls, ##*P* < 0.01 versus Su/hypo + Ad‐Empty exo, *n* = 6. d) Multiple‐IF stainings for the location and expression of CD31 and Ki67 in the lung tissues of mice were detected, with quantification of percent of Ki67 positive nuclei expression relative to total nuclei in CD31 positive cells, ****P < 0.0001 versus Normal controls, ##*P* < 0.01 versus Su/hypo + Ad‐Empty exo, *n* = 6. e) IF staining for the location and expression of NF*κ*B p65 in the lung tissues of mice, quantification of percent of NF*κ*B p65 positive nuclei expression relative to total nuclei in the pulmonary arteries (80–150 µm), ****P < 0.0001 versus Normal controls, ##*P* < 0.01 versus Su/hypo + Ad‐Empty exo, *n* = 6. f) Representative western blotting assay for the protein expression of PCNA, cyclin D1, p‐NF*κ*B p65, and NF*κ*B p65 in the lung tissues of mice, **P < 0.01, ****P < 0.0001 versus Normal controls, ###*P* < 0.001, ####*P* < 0.0001 versus Su/hypo + Ad‐Empty exo, *n* = 6–7. Normal cons: Normal control, conditions.

Further, the role of SOX17‐associated exosomes in SU5416/hypoxia (Su/hypo)‐induced PH was examined. The distribution of SOX17‐associated exosomes labeled using PKH67 in mice after intravenous tail injection was first confirmed by the appearance of green fluorescence in the vasculature of different organs, including lung, liver, and kidney tissues (Figure S10a,b, Supporting Information). Treatment with HPAECs‐derived exosomes through intravenous tail injection once every 3 d for 21 d showed no effect on the hemodynamics, body weight, and vascular morphology of the normal mice (Figure S11a–c, Supporting Information). Mice were treated with Su/hypo for 21 d and injected with SOX17‐associated exosomes once every 3 d (**Figure** **3**a). The increased levels of RVSP, right ventricular hypertrophy (RV/LV+S), and RV weight/tibial length (RVH) of mice stimulated by Su/hypo were significantly prevented by SOX17‐associated exosomes (Figure 3b). The pulmonary vascular remodeling induced by Su/hypo was also alleviated by SOX17‐associated exosomes (Figure 3c). Moreover, SOX17‐associated exosomes downregulated the number of dual‐positive Ki67^+^/CD31^+^ cells in the pulmonary arterial endothelium of PH mice (Figure 3d), and decreased the increased number of positive Ki67^+^ expression in the pulmonary arterial media and adventitia (without quantitation). Moreover, SOX17‐associated exosomes inhibited expression of nuclear NF*κ*B p65 detected in the pulmonary arteries of PH mice (Figure 3e). Western blots further verified that the increased expression of PCNA, cyclinD1, p‐NF*κ*B p65, and nuclear NF*κ*B p65 protein were repressed in the pulmonary tissues from PH mice (Figure 3f). Thus, SOX17‐associated exosomes resist endothelium dysfunction and maintain pulmonary vascular homeostasis in PH.

### 2.3 SOX17 Directly Induces the Transcription and Promotes Exosome‐Mediated Release of miR‐224‐5p and miR‐361‐3p

**Figure 4:**
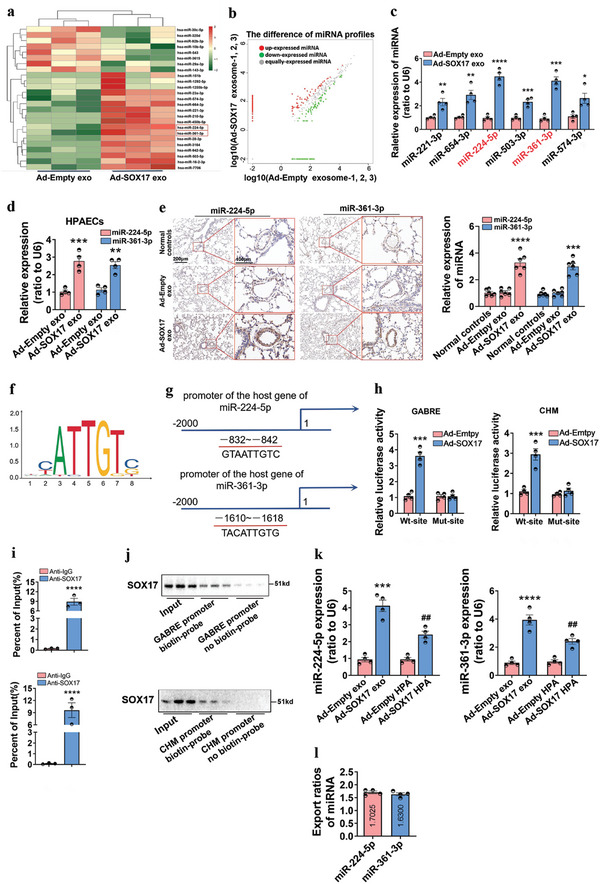
SOX17 induces the expression of miR‐224‐5p and miR‐361‐3p at the transcriptional level and stimulates the release of miR‐224‐5p and miR‐361‐3p into intercellular space through exosomes. a,b) Heat map and scatter diagram of SOX17‐induced changes in exosome‐associated miRNA expression. Exosomes were collected from HPAECs treated with Ad‐Empty or Ad‐SOX17 to perform unsupervised hierarchical clustering. Each row represents one miRNA, and each column represents one sample. c) qRT‐PCR assay for the expression of the selected six differential miRNAs in SOX17‐associated exosomes, *P < 0.05, **P < 0.01, ***P < 0.001, ****P < 0.0001 versus Ad‐Empty exo, *n* = 4. d) qRT‐PCR assay for the expression of miR‐224‐5p and miR‐361‐3p in HPAECs treated with SOX17‐associated exosomes, **P < 0.01, ***P < 0.001 versus Ad‐Empty exosomes, *n* = 4. e) RNA‐ISH assay for the expression of miRNA‐224‐5p and miR‐361‐3p in the lung tissues of mice treated with SOX17‐associated exosomes through intravenous injection, values are mean fold‐changes of controls, ***P < 0.001, ****P < 0.0001 versus Ad‐Empty exo, *n* = 6. f) The DNA motif for SOX17 was obtained from JASPAR. g) The binding sites in the promoter of the host gene of miR‐224‐5p (GABRE) and miR‐361‐3p (CHM) were predicted by miRIAD and JASPAR. (h) The regulation of SOX17 in the promoter of the host gene of miR‐224‐5p (GABRE) and miR‐361‐3p (CHM) were detected by luciferase reporter assay, ***P < 0.001 versus Ad‐Empty, *n* = 4. i,j) The combination between SOX17 and the promoters of the host gene of miR‐224‐5p and miR‐361‐3p was verified by ChIP and DNA pull‐down assays, ****P < 0.0001 versus Anti‐lgG, *n* = 3. k) The expression of miR‐224‐5p and miR‐361‐3p in SOX17‐associated exosomes as well as SOX17‐overexpressing HPAECs were examined by qRT‐PCR, ***P < 0.001, ****P < 0.0001 versus Ad‐Empty exo group, ##*P* < 0.01 versus Ad‐Empty HPA group, *n* = 4. l) Export ratios of miR‐224‐5p and miR‐361‐3p in SOX17‐associated exosomes relative to SOX17‐overexpressing HPAECs.

Exosomal miRNAs are stably stored and transferred by exosomes at a distance to induce transcriptomic changes, in essence acting as important intercellular messengers.^[^ ^17^, ^18^ ^]^ The proportion of some miRNAs is higher in exosomes than in their parent cells.^[^ ^19^ ^]^ Therefore, we used miRNA profiling to explore the downstream signaling of SOX17‐associated exosomes. A total of 453 differentially expressed miRNAs was amassed among all samples, and 34 of these showed significant differential expression with *P*‐value < 0.05 (Table S1, Supporting Information). Among them, the top 25 differentially expressed miRNAs were obtained after the Benjamini–Hochberg correction (*P*‐adj < 0.05), and their expression values are shown in the heat map and scatter diagram (**Figure** **4**a,b). MiRNA profiles in patients with chronic thromboembolic PH (CTEPH) (GSE56914), in which the patients do not receive medical treatments, has a strict size and collection criteria of samples. Besides, CTEPH shares the similar pathological mechanisms of pulmonary vascular remodeling to PH.^[^ ^20^, ^21^ ^]^ Therefore, the above top 25 miRNAs were compared with the miRNA profiles (GSE56914). Six miRNAs, including miR‐221‐3p, miR‐361‐3p, miR‐654‐3p, miR‐224‐5p, miR‐574‐3p, and miR‐503‐3p, showed upregulation in SOX17‐associated exosomes but downregulation in the miRNA profiles with CTEPH. A qRT‐PCR assay further confirmed the increased expression of miR‐221‐3p, miR‐361‐3p, miR‐654‐3p, miR‐224‐5p, and miR‐574‐3p in SOX17‐associated exosomes (Table S2, Supporting Information, and Figure 4c). To evaluate the function of these miRNAs, HPAECs were treated with miRNA mimics before treatment with serum‐free medium, TNF*α*, or hypoxia conditions. MiR‐224‐5p and miR‐361‐3p were excellent inhibitors of apoptosis and the inflammatory response (Figure S12a–c, Supporting Information). Previous studies have confirmed the downregulation of miR‐224‐5p and miR‐361‐3p in IPH patients.^[^ ^22^, ^23^ ^]^ Here, we also verified miR‐224‐5p and miR‐361‐3p were downregulated in the lung tissue homogenate of IPH patients (Figure S12d, Supporting Information) and increased in HPAECs and pulmonary arteries of mice treated with SOX17‐associated exosomes (Figure 4d,e). It is indicating that miR‐224‐5p and miR‐361‐3p were transported to recipient cells by SOX17‐associated exosomes. In addition, SOX17 knockdown also reduced the expression of miR‐224‐5p and miR‐361‐3p in HPAECs, exosomes generated from these HPAECs, and primary PAECs separated from Tie2‐Cre mice treated with AAV9‐shSOX17 (Figure S12e–g, Supporting Information).

To explore the underlying mechanism for inducing miRNA expression in SOX17‐associated exosomes, we used miRIAD (https://www.miriad‐database.org/miRNA) and JASPAR (http://jaspar.genereg.net/) websites to obtain the DNA motif and binding site of SOX17 on the promoters (Figure 4f) and found the predicted binding sites of SOX17 in the promoter of the host genes of miR‐224‐5p (GABRE) and miR‐361‐3p (CHM) (Figure 4g). A luciferase reporter assay was used to verify the binding sites. As shown in Figure 4h, the luciferase activity of wild‐type GABRE and CHM promoters was significantly increased by SOX17 overexpression, whereas that of GABRE and CHM promoters with a mutant binding site revealed no changes. The interactivity of SOX17 with GABRE and CHM promoters was further confirmed by ChIP and DNA pull‐down assays (Figure 4i,j). Moreover, as shown in Figure 4k, after SOX17 overexpression, the intracellular and exosomal level of miR‐224‐5p was increased by 2.41‐fold and 4.12‐fold, respectively, while the level of miR‐361‐3p was increased by 2.40‐fold and 3.93‐fold. Furthermore, the ratio of miRNA level in the exosomes to that in the cells could be defined as miRNA export ratio, which was 1.70 for miR‐224‐5p and 1.63 for miR‐361‐3p (Figure 4l), indicating that SOX17 overexpression could promote exosome‐mediated release of miR‐224‐5p and miR‐361‐3p. These results indicated that SOX17 directly induces the transcription of miR‐224‐5p and miR‐361‐3p and promotes their release through exosomes, which are internalized by recipient HPAECs.

### 2.4 miR‐224‐5p and miR‐361‐3p Mediate the Role of SOX17‐Associated Exosomes and SOX17 in the Endothelial Function and PH

**Figure 5:**
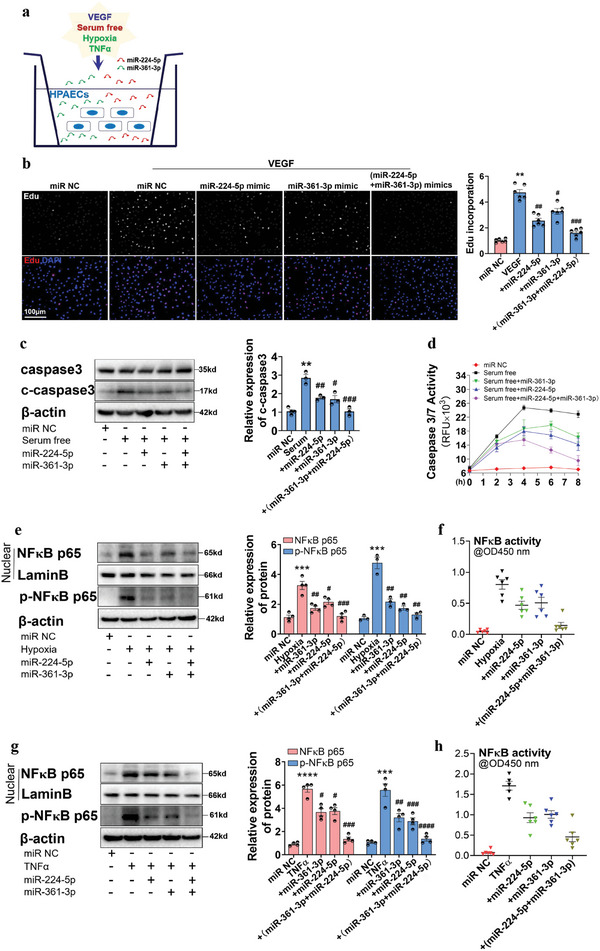
miR‐224‐5p and miR‐361‐3p perform a preferable inhibitory effect on HPAECs dysfunction and mediate the protective role of SOX17‐associated exosomes in endothelial dysfunction. a) The schematic diagram for the conditional treatment of HPAECs. b) EdU assay for the influence of miR‐224‐5p and miR‐361‐3p overexpression on VEGF‐induced proliferation of HPAECs, **P < 0.01 versus miR negative control, #*P* < 0.05, *##P* < 0.01, *###P* < 0.001 versus VEGF + miR negative control, *n* = 6. c) Western blotting assay for the protein expression of caspase3 and cleaved‐caspase3 induced by serum‐free conditions in HPAECs treated with miR‐224‐5p and/or miR‐361‐3p mimics, **P < 0.01 versus miR negative control, #*P* < 0.05, *##P* < 0.01, *###P* < 0.001 versus Serum free + miR negative control, *n* = 3. d) Caspase‐3/7 assay kit was used to detect apoptosis induced by serum‐free culture in HPAECs treated with miR‐224‐5p and/or miR‐361‐3p mimics. e) Western blotting assay for the protein expression of nuclear NF*κ*B p65 and p‐NF*κ*B p65 stimulated by hypoxia in HPAECs treated with miR‐224‐5p and/or miR‐361‐3p mimics, ***P < 0.001 versus miR negative control, *#P* < 0.05, *##P* < 0.01, *###P* < 0.001 versus Hypoxia + miR negative control, *n* = 3–4. f) NF*κ*B p65 transcription factor assay kits for the NF*κ*B activity in HPAECs stimulated by hypoxia in HPAECs treated with miR‐224‐5p and/or miR‐361‐3p mimics, *n* = 6. g) Western blotting assay for the protein expression of nuclear NF*κ*B p65 and p‐NF*κ*B p65 induced by TNF*α* in HPAECs treated with miR‐224‐5p and/or miR‐361‐3p mimics, ***P < 0.001, ****P < 0.0001 versus miR negative control, #*P* < 0.05, *##P* < 0.01, *###P* < 0.001, *####P* < 0.0001 versus TNF*α* + miR negative control, *n* = 4. h) NF*κ*B p65 transcription factor assay kits were used to measure the NF*κ*B activity in HPAECs induced by TNF*α* in HPAECs treated with miR‐224‐5p and/or miR‐361‐3p mimics, *n* = 5–6.

To examine their role in regulation, miR‐224‐5p and miR‐361‐3p were pooled to evaluate proliferation, apoptosis, and inflammation in HPAECs (**Figure** **5**a). The combined treatment of miR‐224‐5p and miR‐361‐3p performed a more effective suppression on VEGF‐induced proliferation (Figure 5b), serum‐free‐induced apoptosis (Figure 5c,d), and hypoxia‐/TNF*α*‐caused inflammation (Figure 5e–h) in HPAECs than when used as single agents. The mediative role of miR‐224‐5p and miR‐361‐3p in the function of SOX17‐associated exosomes was also analyzed. As shown in Figure S12h,i (Supporting Information), combined inhibition of miR‐224‐5p and miR‐361‐3p offset the protective role of SOX17‐associated exosomes in the inflammatory response, even though treatment with each miRNA separately also exerted a partial inhibitory effect. SOX17‐associated exosomes, therefore, work well in endothelial dysfunction mostly through the combined utilization of miR‐224‐5p and miR‐361‐3p. Moreover, we explored whether treatment with miR‐224‐5p and miR‐361‐3p could attenuate Su/hypo‐induced PH. The Tie2‐Cre mice with endothelial knockdown of SOX17 were treated with Su/hypo for 21 d, and during this period, miR‐224‐5p and miR‐361‐3p mimics were injected through tail vein once every 3 d. The overexpression efficiency of miR‐224‐5p and miR‐361‐3p were verified through RNA‐ISH assay (Figure S13a,b, Supporting Information). The increased RVSP, RV/LV+S, RVH, and pulmonary vascular remodeling induced by Su/hypo were significantly blocked through the combined administration of miR‐224‐5p and miR‐361‐3p mimics (Figure S13c,d, Supporting Information). Since AAV9‐shSOX17 had no influence on the Su/hypo induced PH at day 21 post‐Su/hypo, these results indicated that treatment with miR‐224‐5p and miR‐361‐3p attenuated Su/hypo‐induced PH.

The histopathology of PH is characterized by arteriolar intimal proliferation, evolving to aspects of concentric or eccentric laminar sclerosis, medial hypertrophy and adventitial proliferation, with variable inflammatory reactions and occasional aspects of fibrinoid necrosis.^[^ ^24^ ^]^ The aberrant proliferation of PASMCs is a significant inducer for medial hypertrophy, which is partially triggered by endothelial dysfunction.^[^ ^3^ ^]^ Thus, whether SOX17‐associated exosomes regulate the proliferation of PASMCs in a paracrine manner was also analyzed. Human PASMCs (HPASMCs) were co‐cultured with SOX17‐overexpressing HPAECs in transwell dishes (Figure S14a, Supporting Information). The expression of miR‐224‐5p and miR‐361‐3p in HPASMCs incubated with SOX17‐overexpressing HPAECs was significantly increased (Figure S14b, Supporting Information), while the mRNA expression of SOX17 was unchanged (Figure S14c, Supporting Information). Mediation of miR‐224‐5p and miR‐361‐3p in SOX17‐associated exosomes on the proliferation of HPASMCs was also examined. As shown in Figure S14d,e (Supporting Information), SOX17‐overexpressing HPAECs alleviated the proliferation of HPASMCs induced by PDGF, which was reversed by an exosome inhibitor (GW4869) as well as miR‐224‐5p and miR‐361‐3p inhibitors. Interestingly, compared with co‐cultivation conditions, SOX17 overexpression in HPASMCs did not inhibit the proliferation of HPASMCs induced by PDGF (Figure S15a, Supporting Information). These results indicate that SOX17‐associated exosomes mediate the proliferation of PASMCs in a paracrine manner mainly through miR‐224‐5p and miR‐361‐3p.

### 2.5 Exosomal miR‐224‐5p and miR‐361‐3p Modulate Endothelial Function through Targeting NR4A3 and PCSK9

**Figure 6:**
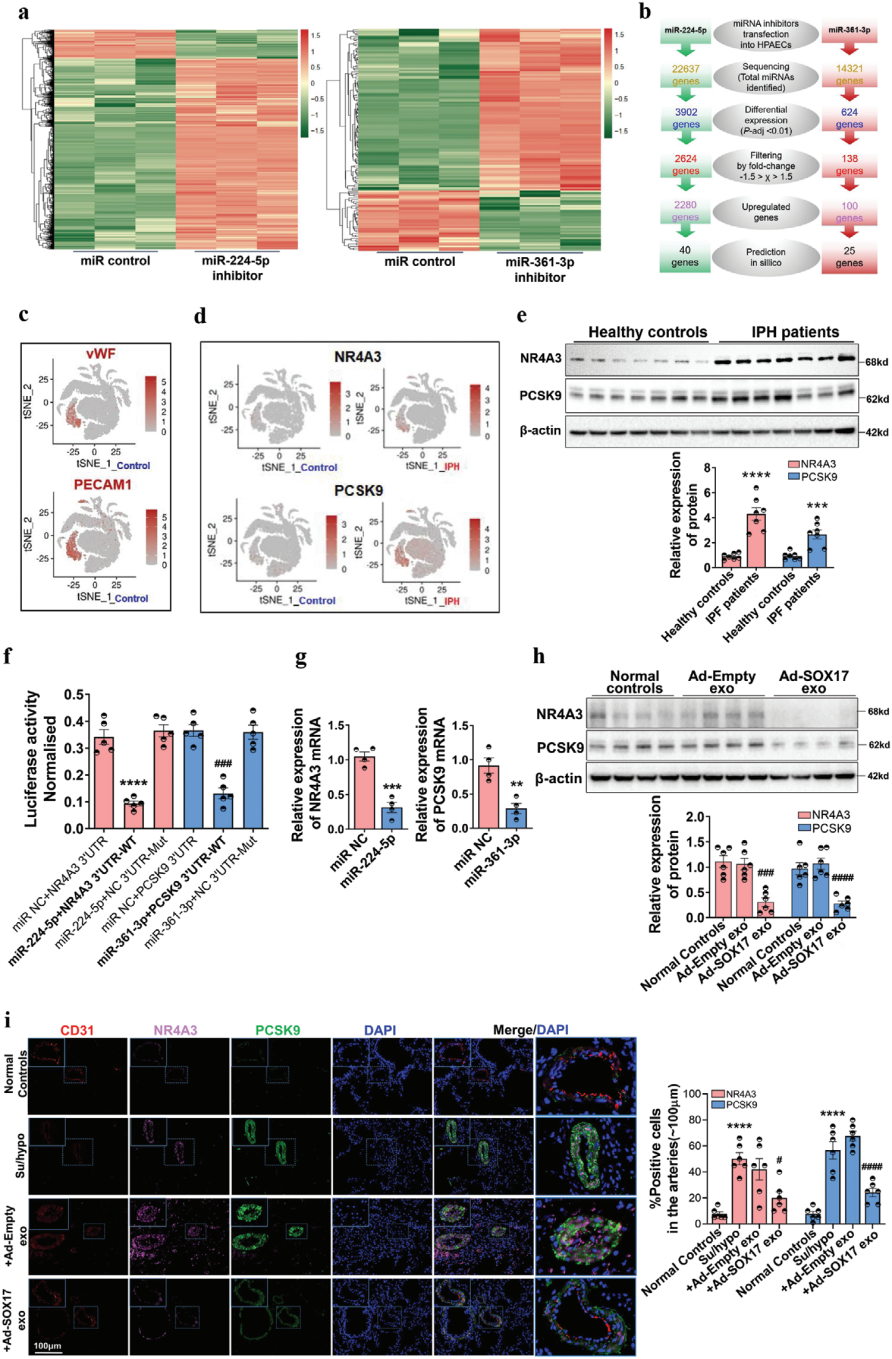
NR4A3 and PCSK9 were selected by RNA sequencing and single cell populations and verified to be the targets of SOX17‐associated exosomes. a) A heat map was constructed for HPAECs transfected with miR‐224‐5p or miR‐361‐3p inhibitor. Red color represents an expression level above mean and green color represents expression level lower than mean. b) A diagram for exploration of the potential miRNA targets in PH. HPAECs were transfected with miRNA inhibitors and subjected to RNA sequencing. Pairwise differential expression analysis was performed based on a model using the negative binomial distribution. *P* values were adjusted for multiple test correction using the Benjamini–Hochberg procedure. miRmap and PITA database were used to predict the potential target of miRNA. c) The endothelial cell clusters were annotated in a single cell population (GSE169471) of healthy donors via vWF and PECAM1. d) The expression of NR4A3 and PCSK9 by cells was selected by stepwise regression models in a single cell population (GSE169471) with healthy controls and IPH patients. e) Western blotting assay for the expression of NR4A3 and PCSK9 in the lung homogenates from healthy controls and IPH patients, ***P < 0.001, ****P < 0.0001 versus healthy controls, *n* = 7. f) The luciferase reporter assay was used to verify the binding site of miRNAs on the predicted targets, ****P < 0.0001 versus miR NC + NR4A3 3′UTR, ###*P* < 0.01 versus miR NC + PCSK9 3′UTR, *n* = 4. g) qRT‐PCR assay for the mRNA expression of NR4A3 and PCSK9 in HPAECs treated with miR‐224‐5p or miR‐361‐3p mimics, **P < 0.01, ***P < 0.001 versus miR NC, *n* = 4. h) Western blotting assay for the expression of NR4A3 and PCSK9 in the lung homogenates from mice treated with SOX17‐associated exosomes through tail veins injection, ###*P* < 0.001, ####*P* < 0.0001 versus Ad‐Empty exo, *n* = 6. i) Multi‐IF staining assay for the co‐expression and location of CD31, NR4A3, and PCSK9 in the lung tissues of healthy mice, PH mice, and PH mice injected with SOX17‐associated exosomes, ****P < 0.0001 versus Normal Controls, #*P* < 0.05, ####*P* < 0.0001 versus Su/hypo + Ad‐Empty exo group, *n* = 6.

To identify the downstream targets, RNA profiling was conducted in HPAECs transfected with miR‐224‐5p and miR‐361‐3p inhibitors. The Benjamini–Hochberg method was used to adjust the *P*‐values to correct for multiple testing. We identified 2280 upregulated transcripts and 344 downregulated transcripts for miR‐224‐5p inhibition, and 100 upregulated transcripts and 37 downregulated transcripts for miR‐361‐3p inhibition (fold‐change > 1.5, adjusted *P*‐value < 0.01). Heat maps and unsupervised hierarchical clustering were used to summarize differential gene expression, shown in **Figure** **6**a. The differential transcripts were further evaluated via GO (Gene Ontology) and KEGG (Kyoto Encyclopedia of Genes and Genomes) analysis. Significant associations were found with the TNF signaling pathway (*P*‐adj  =  1.19 × 10^−10^), NF‐*κ*B signaling pathway (*P*‐adj  =  5.94 × 10^−8^), IL‐17 signaling pathway (*P*‐adj  =  9.61× 10^−7^), apoptosis (*P*‐adj  =  7.73× 10^−5^), TGF‐*β* signaling pathway (*P*‐adj  =  8.72 × 10^−3^), and the PI3K‐Akt signaling pathway (*P*‐adj  =  2.4 × 10^−3^) (Figure S16a,b and Table S3, Supporting Information).

Furthermore, the selected 2280 upregulated differential transcripts of miR‐224‐5p inhibition and 100 upregulated differential transcripts of miR‐361‐3p inhibition were compared with the list of predicted target genes of miR‐224‐5p and miR‐361‐3p in miRmap and PITA database to screen for intersection genes (Figure 6b). Pathway enrichment analysis was conducted for the 65 intersection genes of RNA‐Seq‐validated target genes and *in silico* predicted target genes, and 35 intersection genes showed regulation in cell proliferation, inflammation, apoptosis, and angiogenesis (Table S4, Supporting Information). The crucial genes for the regulation of endothelial cell proliferation, migration, and inflammatory response targeted by miR‐224‐5p included *OLR1*, *NR4A3*, *ICAM1/4*, *IL6*, and *MMP12/13*, whereas the targets of miR‐361‐3p included *vWF*, *IL21R*, *PCSK9*, and *NOG*. To filter the precise targets of miRNAs, we analyzed the lung microenvironment of healthy controls with a single‐cell sequencing dataset (GSE169471) and clustered cell populations using endothelial biomarkers including vWF and PECAM1 (Figure 6c). The expression of above 36 intersection genes were examined and selected by stepwise regression models in the above single cell population. As revealed in Figure 6d, NR4A3 and PCSK9 was mainly expressed by endothelial cluster and showed an significantly upregulated in IPH patients, which have been reported to regulate endothelial proliferation, angiogenesis, and blood vessel endothelial cell migration.^[^ ^25^, ^26^, ^27^, ^28^, ^29^, ^30^ ^]^ The upregulation of NR4A3 and PCSK9 proteins was further verified in the pulmonary tissue homogenates from IPH patients (Figure 6e). To confirm the direct regulation of miR‐224‐5p and miR‐361‐3p in their target genes, miRmap and PITA database were used to predict the potential binding sites, which are shown in Figure S17a (Supporting Information). The luciferase reporter assay and qRT‐PCR confirmed the targeted inhibition of miR‐224‐5p and miR‐361‐3p on the expression of NR4A3 and PCSK9 (Figure 6f,g). Furthermore, the influence of SOX17‐associated exosomes on NR4A3 and PCSK9 expression in vivo was detected. As shown in Figure 6h, SOX17‐associated exosomes significantly repressed the expression of NR4A3 and PCSK9 in the pulmonary tissue homogenates of the normal mice. In Su/hypo‐induced PH mice, the upregulation of NR4A3 and PCSK9 in the remodeling pulmonary arteries was also blocked by SOX17‐associated exosomes (Figure 6i and Figure S18, Supporting Information). Besides, the regulation of SOX17 knockdown on the expression of NR4A3 and PCSK9 proteins were also detected. SOX17 siRNA significantly increased the expression of PCSK9 and NR4A3 in HPAECs (Figure S19a, Supporting Information), while endothelial‐specific SOX17 knockdown in the Tie2‐Cre mice did not affect the expression of PCSK9 and NR4A3 in the endothelium of pulmonary arteries (Figure S19b, Supporting Information). Finally, the role of NR4A3 and PCSK9 on mediating the proliferation, inflammation, and apoptosis of HPAECs was further examined. As shown in Figure S20a–d (Supporting Information), silencing NR4A3 suppressed VEGF‐induced proliferation, hypoxia‐caused inflammation, without affecting serum‐free‐induced apoptosis in HPAECs. PCSK9 siRNA significantly blocks VEGF‐induced proliferation, hypoxia‐caused inflammation, and serum‐free‐induced apoptosis in HPAECs (Figure S20e–h, Supporting Information). It is suggested that high expression of NR4A3 and PCSK9 might exacerbate endothelium dysfunction of pulmonary artery and thereby promote vascular remodeling and PH.

Endothelial colony‐forming cells (ECFCs) often serve as surrogates for lung vascular diseases.^[^ ^31^, ^32^ ^]^ The mRNA expression of SOX17, miR‐224‐5p, miR‐361‐3p, NR4A3, and PCSK9 was verified in ECFCs from patients with IPH. Compared with controls, the IPH group had decreased expression of SOX17, miR‐224‐5p, and miR‐361‐3p, but increased expression of NR4A3 and PCSK9 (Figure S21a, Supporting Information). ECFCs from patients with IPH showed abnormal tube formation and proliferation, which were prevented by treatment with miR‐224‐5p and miR‐361‐3p mimics or NR4A3 and PCSK9 siRNA (Figure S21b–d, Supporting Information). These results demonstrated that NR4A3 and PCSK9 are the targets of miR‐224‐5p and miR‐361‐3p in regulating endothelial function.

## 3 Discussion

**Figure 7:**
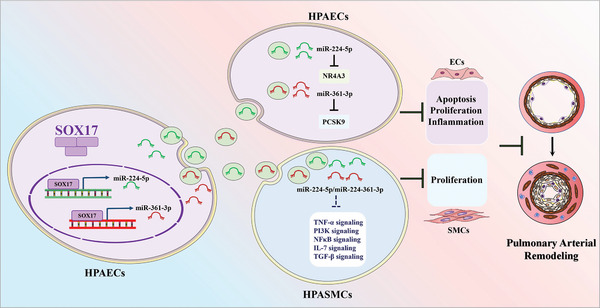
SOX17 mediates the autocrine and paracrine regulation of PAECs on vascular homeostasis through exosomes.

In this study, we confirmed that the PAECs‐derived exosomes improve endothelial dysfunction of pulmonary arteries induced by multiple factors in an autocrine manner and that SOX17 is a critical gene in maintaining endothelial function through this process. In addition, SOX17 also suppressed the aberrant proliferation of PASMCs in the same manner. SOX17‐associated exosomes attenuated pulmonary arterial remodeling and PH in Su/hypo‐treated mice, suggesting their potential therapeutic role in PH. Mechanistic studies uncovered that SOX17 promoted transcription and release of miR‐224‐5p and miR‐361‐3p, thereby targeting NR4A3 and PCSK9 to improve endothelial dysfunction (**Figure** **7**).

ECs have a strong secretory function, and their secretions regulate the phenotype of vascular cells in an autocrine or paracrine manner, thereby mediating vascular function. Classically, these secretions are thought to be cytokines and gas molecules with biological activity, such as interleukin‐8, endothelin 1, NO, and SO_2_.^[^ ^33^, ^34^, ^35^ ^]^ However, growing evidence demonstrates that exosomes are an important way for ECs to release their contents and regulate blood vessels.^[^ ^36^ ^]^ In PH, exosomes are also crucial mediators of cell‐to‐cell communication and regulation during pulmonary arterial remodeling.^[^ ^5^, ^6^, ^7^ ^]^ Nevertheless, the role of exosomes in the autocrine regulation of PAECs is not well understood. We found that normal HPAECs improved the function of a small number of injured PAECs via exosomes. This suggests a tolerance mechanism where in the initial stage of PH, normal PAECs maintain the function of adjacent, injured PAECs by releasing exosomes into the intercellular space, thereby resisting or slowing the development of PH. Preclinical studies demonstrated the therapeutic effects of exosomes in various diseases, including PH.^[^ ^37^, ^38^ ^]^ However, it is important to note that exosomes are just carriers for material delivery. Their biological activity depends on their contents, which are determined by the cell of origin. Therefore, modifying the cell of origin to increase the number of contents with therapeutic properties may enable exosomes to exert better therapeutic effects. In this study, we modified the expression of SOX17 in HPAECs, which is a novel PH, and found that overexpressing SOX17 enhanced the protective effect of exosomes on endothelial function. Moreover, SOX17‐associated exosomes exhibited a promising therapeutic effect on PH in mice. These findings uncover the role and mechanism of SOX17 in maintaining endothelial function and vascular homeostasis and suggest that modifying specific genes could be a feasible way to enhance the therapeutic effect of exosomes.

On the other hand, SOX17 may also mediate the balance of TGF*β*s and BMPs signaling, which is associated with vascular homeostasis in PH. Enhanced TGF*β*s signaling and impaired BMPs signaling have been confirmed to be responsible for the development of PH.^[^ ^39^ ^]^ It is showed that SOX17 interacts with Smad3, thereby causing the loss of expression of TGF*β*1 target genes in epithelial cells.^[^ ^40^ ^]^ During endothelial to haematopoietic transition, SOX17 is increased upon the activation of TGF*β* signaling, and impairs the transition and blood cell formation.^[^ ^41^ ^]^ In BMPs signaling, there is a positive feedback loop between SOX17 and BMP2, which triggers human embryonic lineage commitment and the transition of pluripotent stem cells to cardiac progenitors.^[^ ^42^ ^]^ Moreover, SOX17 is also associated with Wnt and Notch signaling,^[^ ^9^, ^43^, ^44^, ^45^ ^]^ whose enhancement contributes to the pulmonary vascular remodeling and PH.^[^ ^46^, ^47^ ^]^ It is suggested that SOX17 might mediate multiple vital pathways in PH development, which deserves further studies.

To investigate the underlying mechanism of SOX17‐associated exosomes on attenuating pulmonary arterial remodeling, we performed high‐throughput sequencing of miRNAs associated with exosomes. Further, the above top 25 miRNAs were compared with the reported miRNA profiles in patients with CTEPH (GSE56914). Although the datasets of miRNA profile in IPH were found, the patients in these datasets, such as GSE67597, were medically treated, which may affect the original expression profile of miRNAs. It is well accepted that pulmonary vascular remodeling plays a crucial role in the development of CTEPH, and shares the similar pathological mechanisms of PH.^[^ ^20^, ^21^ ^]^ Therefore, we used GSE56914 to screen the potential miRNAs. Six miRNAs induced by SOX17 but downregulation in the miRNA profiles with CTEPH (GSE56914). Real‐time PCR results further confirmed the upregulation of the six miRNAs in the SOX17‐associated exosomes. Then, the functional experiment further verified the inhibiting effect of miR‐224‐5p and miR‐361‐3p on the increased activity of caspase 3 induced by serum‐free and the increased activity of NFĸB p65 induced by hypoxia and TNF*α*. The reduced expression of miR‐224‐5p and miR‐361‐3p were further confirmed in the lung tissues of IPH patients, which has also been reported in PH‐associated studies.^[^ ^22^, ^23^ ^]^ miR‐224‐5p and miR‐361‐3p alone played a protective role under various experimental conditions, and their combined application greatly enhanced this efficiency. It has been demonstrated that the combined application of multiple miRNAs in disease‐relevant pathways mediates cancer progression^[^ ^48^, ^49^ ^]^ and cardiac dysfunction.^[^ ^50^, ^51^ ^]^ Although the roles of miR‐224‐5p and miR‐361‐3p in PH have not been confirmed, their targets significantly associated with crucial regulatory signaling factors in PH, including TNF‐*α*, PI3K‐AKT, VEGF, NF‐*κ*B, BCL2, and toll‐like receptor signaling. The synergism of the combined treatment observed in vitro and in vivo can be partly explained by the multiple pathways associated with the gene targets of miRNAs.

In pulmonary artery fibroblasts isolated from patients with IPH, miR‐224 is downregulated, and its role is unclear.^[^ ^22^ ^]^ The miR‐224 mimic suppresses the expression of TGF‐*β*, MMP9, TIMP‐1, ROR*γ*t, and inflammation in lung tissues of asthmatic mice,^[^ ^52^ ^]^ weakens apoptosis by reducing caspase‐3 and ROS production in cerebral ischemic/reperfusion injury,^[^ ^53^ ^]^ and blocks the proliferation of several cell types,^[^ ^54^, ^55^, ^56^ ^]^ implying that miR‐224 supplementation may play a protective role against the proliferation and inflammation of vascular cells. To explore the downstream mechanisms, we observed contemporaneous, opposing changes in the expression of miR‐224‐5p and its target, NR4A3, in the remodeled lung vasculatures of patients with PH and Su/hypo‐treated mice. NR4A3 belongs to the nuclear receptor 4A (NR4A) family, which regulates vascular smooth muscle cell function and is associated with vascular diseases.^[^ ^57^, ^58^ ^]^ Multiple inducing factors, including PDGF‐BB, thrombin, serum, and LDL, stimulated NR4A3 expression in SMCs.^[^ ^25^, ^59^, ^60^ ^]^ Takashi et al. found that SMCs isolated from NR4A3‐deficient mice exhibited decreased cell proliferation and identified cyclin D1 and D2 as NR4A3 target genes.^[^ ^25^ ^]^ In addition, NR4A3 accumulates in atherosclerotic lesions in patients and porcine balloon‐injured vascular injuries.^[^ ^25^, ^59^ ^]^ In this study, NR4A3 mRNA and protein were highly expressed in blood derived IPH ECFCs and in the remodeled lung vascular tissue from patients with IPH.

miR‐361 plays a protective role in vascular endothelial injury and inflammation induced by high glucose,^[^ ^61^ ^]^ and inhibited cell proliferation and migration. Specifically, miR‐361 expression was downregulated in the plasma of patients with PH, and its mimic alleviated the serotonin‐induced proliferation of HPASMCs.^[^ ^23^ ^]^ Further, miR‐361 sponges TNF‐*α* in PH.^[^ ^62^, ^63^ ^]^ In this study, we found that miR‐361‐3p expression was decreased, and its target gene PCSK9 expression was induced in ECFCs and lung tissues from patients with IPH and in PH mice. PCSK9 is reportedly secreted by vascular endothelial cells and regulates toll‐like receptor 4 expression, NF‐*κ*B activation, and apoptosis.^[^ ^27^ ^]^ PCSK9 also interacts with oxidized LDL receptor‐1 (LOX‐1) in a mutually facilitative fashion,^[^ ^64^ ^]^ and LOX‐1 has been shown to play a crucial role in PH.^[^ ^65^, ^66^ ^]^

The protective role of SOX17 exosomes in pulmonary vascular homeostasis may be attributed to the cumulative inhibition of various target genes of miR‐224‐5p and miR‐361‐3p more than NR4A3 and PCSK9 in this study. However, miRNAs can simultaneously and cooperatively regulate the expression of multiple target genes,^[^ ^67^ ^]^ though identifying the individual contributions of miR‐224‐5p and miR‐361‐3p gene targets is beyond the objective of this study. In addition, the underlying mechanism by which SOX17 induces expression of miR‐224‐5p and miR‐361‐3p in exosomes of HPAECs has not been evaluated. We infer that this may due to the binding of SOX17 to the enhancer or promoter regions of miRNAs, leading to hypomethylation of pri‐miRNA promoters, post‐transcriptional processing through indirect transcriptional regulation by CpG demethylation factors or via intermediate agents.^[^ ^68^, ^69^ ^]^

Our results showed that SOX17 selectively increased the expression of miR‐224‐5p and miR‐361‐3p inside exosomes, yet the mechanism of miRNA sorting is not well understood. Nevertheless, certain specific consensus sequence motifs (EXOmotifs) associated with miRNAs may control the loading of specific RNAs into exosomes.^[^ ^70^ ^]^ miR‐224‐5p has three potential EXOmotifs that differ from the consensus sequence at one nucleotide position (GGUG, CCUU, CACU), whereas miR‐361‐3p has two motifs, one with a perfect match to the consensus sequence (CCCU) and another showing alteration in one nucleotide (GGUG).

Taken together, we have shown that SOX17‐overexpressing dependent endothelial exosomes can imitate the homoeostatic effects of endothelial SOX17 in PH. This is an important study to evaluate the dysfunctional SOX17 signaling in PH and show the potential role of SOX17‐associated exosomes in treating PH and other diseases associated with inflammation, endothelial injury, proliferation, and apoptosis.

## 4 Experimental Section

The details for the procedures, materials used, and analytical methods are available in the Supporting Information.

### Quantification and Statistical Analysis

All data acquisition and analysis were performed by investigators blinded to the experimental group. For biochemical analyses, a minimum of three samples per genotype was used for each analysis, whereas in vivo analysis included at least six mice per group. These sample sizes are sufficient to determine whether there is a biologically meaningful difference between different genotypes. For in vitro studies, a sufficiently large number of cells were analyzed to ensure the identification of biologically meaningful differences using methods from studies cited throughout the paper. Moreover, results obtained in vitro were reliably reproduced in at least three independent experiments. All experimental data were expressed as mean ± SEM unless otherwise mentioned. The normality of the variables was tested using the Shapiro–Wilk test. The data from the analysis met the assumptions of the tests and the variance was similar between the experimental groups (if not, the data were not included in the following analysis). Unpaired two‐tailed Student's *t*‐test was used when comparing two experimental groups, whereas three experimental groups were analyzed using one‐way ANOVA followed by Tukey's post hoc test. GraphPad Prism 8.0 was used for calculations and *P*‐values < 0.05 were considered statistically significant.

## Conflict of Interest

The authors declare no conflict of interest.

## Supporting information

Supporting Information

## Data Availability

The data that support the findings of this study are available from the corresponding author upon reasonable request.
