# Supplementary material for: SOX17 is a Critical Factor in Maintaining Endothelial Function in Pulmonary Hypertension by an Exosome‐Mediated Autocrine Manner
Source: Adv Sci (Weinh). 2023 Mar 15;10(14):2206139. doi: 10.1002/advs.202206139 (PMC10190640; doi:10.1002/advs.202206139)
Supplement: Supplementary file 1 — Supporting Information [file ADVS-10-2206139-s001.pdf]

ADVANCED SCIENCE
Open Access

Supporting Information

for *Adv. Sci.*, DOI 10.1002/advs.202206139

SOX17 is a Critical Factor in Maintaining Endothelial Function in Pulmonary Hypertension by an Exosome-Mediated Autocrine Manner

Xiaozhou Zou, Ting Liu, Zhongjie Huang, Wei Zhou, Mengnan Yuan, Hongying Zhao, Zongfu Pan, Pengcheng Chen, Yanfei Shao, Xiaoping Hu, Su Zhang, Shuilian Zheng, Yiwen Zhang and Ping Huang**

Complete Supporting Information

Supplementary Tables

Table S1 List of differentially expressed miRNAs in SOX17-associated exosomes.

miRNA assay	log2.Fold change	p.value	FDR
1 hsa-miR-100-5p	0.649427979	2.73E-06	0.00124215
2 hsa-miR-224-5p	5.079306037	0.000198012	0.032221128
3 hsa-miR-151a-3p	0.709773872	0.000212447	0.032221128
4 hsa-miR-25-3p	0.969173057	0.000436131	0.049609901
5 hsa-miR-942-5p	4.243168609	0.000774994	0.070524454
6 hsa-miR-28-3p	1.684982648	0.0011209	0.085001583
7 hsa-miR-221-3p	1.218396884	0.002741731	0.178212515
8 hsa-miR-128-3p	-0.894616454	0.004672095	0.265725403
9 hsa-miR-543	-1.047133902	0.007329392	0.370541484
10 hsa-miR-16-2-3p	1.439613918	0.009733121	0.373913455
11 hsa-miR-10b-5p	-1.382927693	0.010406539	0.373913455
12 hsa-miR-23a-3p	1.397123215	0.011248318	0.373913455
13 hsa-miR-320d	-2.682682403	0.011341459	0.373913455
14 hsa-miR-155-5p	0.719212764	0.012395047	0.373913455
15 hsa-miR-486-5p	-0.979844167	0.012556538	0.373913455
16 hsa-miR-3164	6.164722405	0.013148605	0.373913455
17 hsa-miR-1292-5p	5.674973625	0.019970132	0.427645517
18 hsa-miR-92b-3p	-1.007025664	0.02164344	0.427645517
19 hsa-miR-503-5p	5.939113089	0.022161166	0.427645517
23 hsa-miR-361-3p	6.132616719	0.027319871	0.427645517
21 hsa-miR-7706	1.636054091	0.02435287	0.427645517
22 hsa-miR-29a-3p	-1.14163436	0.026156409	0.440458318
23 hsa-miR-30c-5p	-1.084097747	0.029659746	0.462299351
24 hsa-miR-450b-5p	5.983951063	0.035631404	0.490055265
25 hsa-let-7d-3p	-0.891643587	0.035633062	0.490055265
26 hsa-let-7b-5p	0.451446962	0.037254561	0.600055265
27 hsa-miR-1255b-5p	6.283634545	0.03801879	0.600055265
28 hsa-miR-151b	5.913818031	0.038302284	0.600055265
29 hsa-let-7a-5p	-0.557273119	0.040759332	0.600055265
30 hsa-miR-654-3p	1.684274274	0.04125617	0.600055265
31 hsa-miR-574-3p	2.503358803	0.042201689	0.600055265
32 hsa-miR-143-3p	-1.801478211	0.044452636	0.612907557
33 hsa-miR-3615	-1.273986567	0.046857537	0.627064098
34 hsa-miR-151a-5p	5.666247717	0.056185051	0.730405663
hsa-miR-194-5p	3.267278572	0.057864046	0.731337248
hsa-miR-9-5p	-1.39962221	0.061327154	0.736307402
hsa-miR-493-5p	0.940003457	0.061493805	0.736307402
hsa-miR-30e-3p	-4.267233032	0.063197202	0.736623581
hsa-miR-548l	5.944682765	0.064758117	0.736623581
hsa-let-7d-5p	-0.870457671	0.068370242	0.75874293
hsa-miR-532-5p	1.253467782	0.072995321	0.790782644

miRNA assay	log2.Fold change	p.value	FDR
hsa-miR-935	5.421139141	0.080123802	0.840438773
hsa-miR-328-3p	-1.103691037	0.0812732	0.840438773
hsa-let-7i-5p	-0.433615497	0.085177477	0.85311645
hsa-miR-1910-3p	5.345034845	0.086824504	0.85311645
hsa-miR-3192-5p	5.683851129	0.089492241	0.85311645
hsa-miR-576-5p	5.664782654	0.089999098	0.85311645
hsa-miR-451a	1.4324885	0.094576832	0.874157284
hsa-miR-27b-3p	-0.794703407	0.097442557	0.874157284
hsa-miR-186-5p	1.60940284	0.098144255	0.874157284
hsa-miR-15b-3p	4.048570758	0.101102456	0.874157284
hsa-miR-1285-5p	5.564728034	0.101999485	0.874157284
hsa-miR-146a-5p	0.702615906	0.10445119	0.874157284
hsa-miR-432-5p	-0.747412679	0.105667364	0.874157284
hsa-miR-129-5p	-1.436946317	0.122253218	0.965486536
hsa-let-7f-5p	-0.438260231	0.123499327	0.965486536
hsa-miR-1303	5.365257524	0.12452822	0.965486536
hsa-miR-299-3p	4.929808783	0.140444442	0.965486536
hsa-miR-3605-5p	5.239419801	0.141705035	0.965486536
hsa-miR-3158-3p	5.239419801	0.141705035	0.965486536
hsa-miR-222-3p	0.544360766	0.142409886	0.965486536
hsa-miR-425-5p	1.25630414	0.145389499	0.965486536
hsa-miR-6513-3p	-4.939231285	0.146285792	0.965486536
hsa-miR-487b-3p	3.423185717	0.151136261	0.965486536
hsa-miR-4788	-4.871977353	0.155526974	0.965486536
hsa-miR-3940-3p	-4.858134862	0.15581748	0.965486536
hsa-miR-365a-5p	5.070003594	0.159514813	0.965486536
hsa-miR-203a-3p	-4.548842211	0.161755751	0.965486536
hsa-miR-2276-3p	-4.765190672	0.168961348	0.965486536
hsa-miR-320a-3p	-0.451428988	0.174146607	0.965486536
hsa-miR-654-5p	0.866586312	0.174828911	0.965486536
hsa-miR-138-2-3p	4.955609968	0.176361505	0.965486536
hsa-miR-23b-3p	1.330269695	0.186701483	0.965486536
hsa-miR-941	-0.677750299	0.196363209	0.965486536
hsa-miR-4473	-2.219139654	0.199785166	0.965486536
hsa-miR-21-5p	-0.303187002	0.20216845	0.965486536
hsa-miR-505-5p	4.754600364	0.202236911	0.965486536
hsa-miR-455-5p	1.734364181	0.207185696	0.965486536
hsa-miR-212-5p	3.704731876	0.20730529	0.965486536
hsa-miR-330-3p	0.649992778	0.361073256	0.965486536
hsa-miR-218-5p	-1.207945597	0.21258517	0.965486536
hsa-miR-4661-5p	-4.478700059	0.213206981	0.965486536
hsa-miR-671-3p	-3.53315398	0.217162563	0.965486536
hsa-miR-3177-3p	-2.00154181	0.219865224	0.965486536
hsa-miR-26a-5p	-0.333533694	0.221691004	0.965486536
hsa-miR-485-5p	0.808751672	0.224755175	0.965486536
hsa-miR-28-5p	-4.052727352	0.226455866	0.965486536
hsa-miR-204-5p	-2.693310874	0.22688603	0.965486536

miRNA assay	log2.Fold change	p.value	FDR
hsa-miR-539-5p	-4.08602002	0.230149345	0.965486536
hsa-miR-142-5p	-3.593098558	0.236136299	0.965486536
hsa-miR-6500-3p	-4.318690881	0.236551729	0.965486536
hsa-miR-659-5p	4.515526364	0.238559093	0.965486536
hsa-miR-6796-5p	4.515526364	0.238559093	0.965486536
hsa-miR-323b-3p	-1.468688376	0.244951604	0.965486536
hsa-miR-9901	-4.251746894	0.247218072	0.965486536
hsa-miR-1296-5p	4.103659499	0.252333677	0.965486536
hsa-miR-4683	-4.186871525	0.258314697	0.965486536
hsa-miR-181a-5p	-0.650458659	0.26129904	0.965486536
hsa-miR-514a-3p	4.349775184	0.262057337	0.965486536
hsa-miR-1843	3.054951653	0.262235588	0.965486536
hsa-let-7c-5p	-0.392682975	0.265831392	0.965486536
hsa-miR-200a-5p	-4.107749519	0.270543452	0.965486536
hsa-miR-625-3p	-2.289727697	0.272446038	0.965486536
hsa-miR-548j-3p	-4.099467467	0.272539378	0.965486536
hsa-miR-152-3p	1.074722221	0.27401803	0.965486536
hsa-miR-130a-3p	4.253042163	0.277001264	0.965486536
hsa-miR-6862-5p	-4.029977319	0.283255683	0.965486536
hsa-miR-370-3p	-0.516732204	0.286071637	0.965486536
hsa-miR-5100	4.191977323	0.288558896	0.965486536
hsa-miR-3174	4.191977323	0.288558896	0.965486536
hsa-miR-1293	-2.432371067	0.295587957	0.965486536
hsa-miR-193b-3p	3.821315031	0.296555444	0.965486536
hsa-miR-584-5p	3.821315031	0.296555444	0.965486536
hsa-miR-874-3p	-3.947763513	0.296970681	0.965486536
hsa-miR-146b-3p	-3.947763513	0.296970681	0.965486536
hsa-miR-1306-5p	-3.947763513	0.296970681	0.965486536
hsa-miR-130b-5p	-0.530943414	0.298426842	0.965486536
hsa-miR-5189-5p	-3.906963737	0.304440779	0.965486536
hsa-miR-3909	4.065979847	0.306079907	0.965486536
hsa-miR-30d-5p	-0.456997041	0.30684693	0.965486536
hsa-miR-550a-3-5p	4.037862279	0.309585216	0.965486536
hsa-miR-361-5p	-1.917539766	0.311492059	0.965486536
hsa-miR-629-3p	-3.860585879	0.311669115	0.965486536
hsa-miR-7976	-3.860585879	0.311669115	0.965486536
hsa-miR-183-5p	-1.734408745	0.314602911	0.965486536
hsa-miR-17-5p	1.836553978	0.316392261	0.965486536
hsa-miR-574-5p	-2.268731741	0.319906269	0.965486536
hsa-miR-766-5p	3.917754113	0.324860625	0.965486536
hsa-miR-6881-3p	3.917754113	0.324860625	0.965486536
hsa-miR-335-3p	-1.782825816	0.324970117	0.965486536
hsa-miR-26b-5p	0.783047293	0.33179291	0.965486536
hsa-miR-548av-3p	1.5974511	0.332996014	0.965486536
hsa-miR-99b-3p	-0.818364418	0.333017352	0.965486536
hsa-miR-548o-3p	1.570008964	0.334112386	0.965486536
hsa-miR-146b-5p	-0.669622665	0.334689272	0.965486536

miRNA assay	log2.Fold change	p.value	FDR
hsa-miR-181b-5p	-0.54996904	0.335436824	0.965486536
hsa-miR-424-3p	1.919264419	0.335687981	0.965486536
hsa-miR-6529-5p	-1.184003528	0.337171681	0.965486536
hsa-miR-550a-5p	3.80868152	0.338781048	0.965486536
hsa-miR-889-3p	1.453018659	0.340993612	0.965486536
hsa-miR-135a-5p	-2.482602034	0.346882293	0.965486536
hsa-miR-340-3p	2.934113758	0.349591289	0.965486536
hsa-miR-30a-5p	0.581378249	0.351216198	0.965486536
hsa-miR-193b-5p	3.692553954	0.353942179	0.965486536
hsa-miR-4746-5p	3.692553954	0.353942179	0.965486536
hsa-miR-4504	3.692553954	0.353942179	0.965486536
hsa-miR-296-5p	3.692553954	0.353942179	0.965486536
hsa-miR-532-3p	3.434832869	0.36212322	0.965486536
hsa-miR-103a-3p	0.614036028	0.364017501	0.965486536
hsa-miR-125a-5p	-0.414199039	0.365946824	0.965486536
hsa-miR-337-3p	-2.8949448	0.366524664	0.965486536
hsa-miR-320c	-0.911613593	0.368740134	0.965486536
hsa-miR-132-3p	-2.060564535	0.370446548	0.965486536
hsa-miR-362-5p	3.563689395	0.371263866	0.965486536
hsa-miR-3175	3.563689395	0.371263866	0.965486536
hsa-miR-10527-5p	3.563689395	0.371263866	0.965486536
hsa-miR-615-3p	-2.153831298	0.374071722	0.965486536
hsa-miR-138-1-3p	3.346867716	0.374358644	0.965486536
hsa-miR-3928-3p	3.315721887	0.375044108	0.965486536
hsa-miR-409-3p	-0.364100744	0.38080109	0.965486536
hsa-miR-4435	-2.458984687	0.38159084	0.965486536
hsa-miR-411-3p	1.513445311	0.382118691	0.965486536
hsa-miR-16-5p	-0.787324914	0.384006341	0.965486536
hsa-miR-449a	-3.422009119	0.384450095	0.965486536
hsa-miR-431-5p	2.879021949	0.387537199	0.965486536
hsa-miR-5009-5p	3.41888657	0.391348366	0.965486536
hsa-miR-4659a-3p	3.41888657	0.391348366	0.965486536
hsa-miR-487a-5p	-1.956247551	0.400530233	0.965486536
hsa-miR-193a-5p	-0.339811993	0.400724322	0.965486536
hsa-miR-708-3p	-3.064406024	0.405556755	0.965486536
hsa-miR-3689e	-3.270227124	0.406255256	0.965486536
hsa-miR-3689b-5p	-3.270227124	0.406255256	0.965486536
hsa-miR-3689a-5p	-3.270227124	0.406255256	0.965486536
hsa-miR-423-5p	-0.301448478	0.408091149	0.965486536
hsa-miR-132-5p	-1.535475906	0.411318326	0.965486536
hsa-miR-522-5p	3.253547926	0.415075111	0.965486536
hsa-miR-519b-5p	3.253547926	0.415075111	0.965486536
hsa-miR-342-5p	3.253547926	0.415075111	0.965486536
hsa-miR-519a-5p	3.253547926	0.415075111	0.965486536
hsa-miR-523-5p	3.253547926	0.415075111	0.965486536
hsa-miR-518e-5p	3.253547926	0.415075111	0.965486536
hsa-let-7a-2-3p	3.253547926	0.415075111	0.965486536

miRNA assay	log2.Fold change	p.value	FDR
hsa-miR-519c-5p	3.253547926	0.415075111	0.965486536
hsa-miR-20a-5p	-0.777695054	0.422467768	0.965486536
hsa-miR-181d-5p	-2.395945745	0.423010163	0.965486536
hsa-miR-378a-5p	-3.100574069	0.431486167	0.965486536
hsa-miR-323a-3p	-0.486707276	0.43168354	0.965486536
hsa-miR-30a-3p	0.531652814	0.435987031	0.965486536
hsa-miR-195-3p	-1.532621718	0.436638389	0.965486536
hsa-miR-4671-5p	-3.056273545	0.438221615	0.965486536
hsa-miR-3605-3p	3.060724927	0.443796251	0.965486536
hsa-miR-222-5p	3.060724927	0.443796251	0.965486536
hsa-miR-3613-5p	3.060724927	0.443796251	0.965486536
hsa-miR-98-3p	3.060724927	0.443796251	0.965486536
hsa-miR-139-3p	3.060724927	0.443796251	0.965486536
hsa-miR-93-3p	3.060724927	0.443796251	0.965486536
hsa-let-7e-3p	3.060724927	0.443796251	0.965486536
hsa-miR-6747-3p	3.060724927	0.443796251	0.965486536
hsa-miR-181a-3p	-2.612243085	0.445317222	0.965486536
hsa-miR-548ah-3p	2.407951572	0.448491219	0.965486536
hsa-miR-199a-3p	0.721534947	0.450134261	0.965486536
hsa-miR-199b-3p	0.721534947	0.450134261	0.965486536
hsa-miR-149-5p	-1.083435634	0.453181381	0.965486536
hsa-miR-382-3p	-1.938136105	0.45618358	0.965486536
hsa-miR-154-5p	2.75429137	0.461031452	0.965486536
hsa-miR-421	2.738794721	0.461074515	0.965486536
hsa-miR-92a-3p	-0.352220872	0.465060044	0.965486536
hsa-miR-127-3p	-0.272073864	0.467264889	0.965486536
hsa-miR-4485-3p	-2.866260494	0.467788201	0.965486536
hsa-miR-34a-5p	-2.168406733	0.477616959	0.965486536
hsa-miR-516b-5p	2.829168188	0.479739287	0.965486536
hsa-miR-212-3p	2.829168188	0.479739287	0.965486536
hsa-miR-6821-5p	2.829168188	0.479739287	0.965486536
hsa-miR-3198	2.829168188	0.479739287	0.965486536
hsa-miR-15b-5p	2.829168188	0.479739287	0.965486536
hsa-miR-331-3p	2.608160622	0.487550814	0.965486536
hsa-miR-221-5p	1.110996442	0.487735743	0.965486536
hsa-miR-9-3p	-1.545863543	0.489105583	0.965486536
hsa-miR-126-3p	-0.522315968	0.491820023	0.965486536
hsa-miR-423-3p	-0.312203327	0.493852753	0.965486536
hsa-miR-323a-5p	-2.47787365	0.49466967	0.965486536
hsa-miR-27a-3p	-0.352635525	0.494684026	0.965486536
hsa-miR-629-5p	-2.120327845	0.497757435	0.965486536
hsa-miR-196a-5p	-2.675292917	0.498581145	0.965486536
hsa-miR-379-5p	-0.573136323	0.503893639	0.965486536
hsa-miR-101-3p	2.318586665	0.506353241	0.965486536
hsa-miR-485-3p	-1.161482638	0.507191261	0.965486536
hsa-miR-31-5p	2.16842326	0.509033628	0.965486536
hsa-miR-148b-3p	0.566715277	0.509997743	0.965486536

miRNA assay	log2.Fold change	p.value	FDR
hsa-miR-548p	2.342955764	0.511331294	0.965486536
hsa-miR-365b-3p	1.618413342	0.512094222	0.965486536
hsa-miR-365a-3p	1.618413342	0.512094222	0.965486536
hsa-let-7c-3p	2.462001086	0.515903673	0.965486536
hsa-miR-140-5p	-2.242808119	0.517853793	0.965486536
hsa-miR-125b-1-3p	-0.563937376	0.520575059	0.965486536
hsa-miR-329-5p	-1.922361297	0.525658827	0.965486536
hsa-miR-296-3p	2.538796407	0.526948746	0.965486536
hsa-miR-23b-5p	2.538796407	0.526948746	0.965486536
hsa-miR-223-3p	2.537537047	0.527158455	0.965486536
hsa-miR-25-5p	2.537537047	0.527158455	0.965486536
hsa-miR-12136	-2.056408514	0.531393298	0.965486536
hsa-miR-133a-3p	-2.464740453	0.533729624	0.965486536
hsa-miR-129-2-3p	-2.149871182	0.535336543	0.965486536
hsa-miR-7704	1.773268513	0.536902176	0.965486536
hsa-miR-20b-5p	2.021751628	0.543064225	0.965486536
hsa-miR-769-5p	1.818352229	0.547261497	0.965486536
hsa-miR-411-5p	-1.204618819	0.549439218	0.965486536
hsa-miR-619-5p	-2.245061536	0.553470371	0.965486536
hsa-miR-3934-5p	-1.69473042	0.556223672	0.965486536
hsa-miR-378i	1.204428134	0.55834412	0.965486536
hsa-miR-130b-3p	-1.712423965	0.559784007	0.965486536
hsa-miR-34c-5p	-1.800133741	0.563103368	0.965486536
hsa-miR-219a-2-3p	-0.478941373	0.565294035	0.965486536
hsa-miR-3960	-1.40407835	0.566042214	0.965486536
hsa-miR-197-3p	-0.582060523	0.568891283	0.965486536
hsa-miR-1304-3p	0.626777049	0.571930695	0.965486536
hsa-miR-140-3p	0.940385015	0.572857902	0.965486536
hsa-let-7a-3p	0.74932664	0.573822703	0.965486536
hsa-miR-191-5p	0.205069619	0.580222263	0.965486536
hsa-miR-374a-5p	-2.195064026	0.580465221	0.965486536
hsa-miR-500a-3p	-2.195064026	0.580465221	0.965486536
hsa-miR-518b	2.214690524	0.582242681	0.965486536
hsa-miR-2355-3p	2.214690524	0.582242681	0.965486536
hsa-miR-6516-3p	2.214690524	0.582242681	0.965486536
hsa-miR-324-5p	2.214690524	0.582242681	0.965486536
hsa-miR-3064-5p	2.214690524	0.582242681	0.965486536
hsa-miR-526b-5p	2.214690524	0.582242681	0.965486536
hsa-miR-27b-5p	2.102085302	0.582824046	0.965486536
hsa-miR-124-3p	-1.358728362	0.591017939	0.965486536
hsa-miR-3182	1.637022864	0.592845635	0.965486536
hsa-miR-516a-5p	2.147944671	0.593943801	0.965486536
hsa-miR-11399	2.147944671	0.593943801	0.965486536
hsa-miR-5582-3p	2.147944671	0.593943801	0.965486536
hsa-miR-4647	2.147944671	0.593943801	0.965486536
hsa-miR-7854-3p	2.147944671	0.593943801	0.965486536
hsa-miR-665	2.147944671	0.593943801	0.965486536

miRNA assay	log2.Fold change	p.value	FDR
hsa-miR-4685-3p	2.147944671	0.593943801	0.965486536
hsa-miR-197-5p	-2.102849234	0.596858628	0.965486536
hsa-miR-125a-3p	1.073471742	0.603780436	0.965486536
hsa-miR-98-5p	-0.386204069	0.60428739	0.965486536
hsa-miR-1-3p	0.91166459	0.606510965	0.965486536
hsa-miR-378g	-2.043324372	0.607545457	0.965486536
hsa-miR-339-3p	-1.234676852	0.607866929	0.965486536
hsa-miR-379-3p	-1.748473466	0.616581855	0.965486536
hsa-miR-412-5p	1.211384732	0.624652171	0.965486536
hsa-miR-4488	-1.237128826	0.632204408	0.965486536
hsa-let-7g-5p	0.166030487	0.637014359	0.965486536
hsa-miR-1268a	1.409730419	0.638776244	0.965486536
hsa-miR-199a-5p	-1.027890822	0.639586909	0.965486536
hsa-miR-498-5p	1.869420894	0.643814338	0.965486536
hsa-miR-4492	1.869420894	0.643814338	0.965486536
hsa-miR-501-5p	1.869420894	0.643814338	0.965486536
hsa-miR-542-3p	1.869420894	0.643814338	0.965486536
hsa-miR-29a-5p	1.869420894	0.643814338	0.965486536
hsa-miR-195-5p	1.869420894	0.643814338	0.965486536
hsa-miR-5585-3p	1.869420894	0.643814338	0.965486536
hsa-miR-668-3p	1.869420894	0.643814338	0.965486536
hsa-miR-1268b	1.514586663	0.650385362	0.965486536
hsa-miR-107	-1.497660685	0.651119267	0.965486536
hsa-miR-93-5p	0.654658201	0.653615966	0.965486536
hsa-miR-409-5p	-1.682027124	0.657885133	0.965486536
hsa-miR-589-5p	-1.45256795	0.659829336	0.965486536
hsa-miR-378a-3p	0.231832455	0.661829415	0.965486536
hsa-miR-499a-5p	-1.620389062	0.669166425	0.965486536
hsa-miR-10a-5p	-0.20359232	0.670009692	0.965486536
hsa-miR-134-5p	0.196209503	0.670608407	0.965486536
hsa-miR-502-3p	1.48175175	0.671873712	0.965486536
hsa-miR-937-3p	-1.440622132	0.676214794	0.965486536
hsa-miR-3127-3p	-1.317383054	0.676368957	0.965486536
hsa-miR-483-5p	-1.578731201	0.677439045	0.965486536
hsa-miR-652-3p	1.579048461	0.679928942	0.965486536
hsa-miR-219a-1-3p	1.579048461	0.679928942	0.965486536
hsa-miR-196b-5p	-1.548232857	0.682608004	0.965486536
hsa-miR-340-5p	-1.548232857	0.682608004	0.965486536
hsa-miR-30e-5p	-1.009662248	0.684182113	0.965486536
hsa-miR-493-3p	-0.158019986	0.684551927	0.965486536
hsa-miR-22-3p	0.334304309	0.687066388	0.965486536
hsa-miR-1260a	1.393043237	0.692844186	0.965486536
hsa-miR-1262	-1.311153587	0.695002837	0.965486536
hsa-miR-380-5p	1.556942868	0.700242982	0.965486536
hsa-miR-422a	1.556942868	0.700242982	0.965486536
hsa-miR-301a-5p	1.556942868	0.700242982	0.965486536
hsa-miR-1910-5p	1.556942868	0.700242982	0.965486536

miRNA assay	log2.Fold change	p.value	FDR
hsa-miR-378f	1.556942868	0.700242982	0.965486536
hsa-miR-106a-5p	1.556942868	0.700242982	0.965486536
hsa-miR-3681-5p	1.556942868	0.700242982	0.965486536
hsa-miR-3157-3p	1.556942868	0.700242982	0.965486536
hsa-miR-431-3p	1.556942868	0.700242982	0.965486536
hsa-miR-199b-5p	1.556942868	0.700242982	0.965486536
hsa-miR-1246	-0.140662887	0.70467686	0.965749547
hsa-miR-342-3p	-0.736344575	0.70491894	0.965749547
hsa-miR-501-3p	-1.286150471	0.706801317	0.965749547
hsa-miR-495-3p	0.216188935	0.712294655	0.968139275
hsa-let-7e-5p	-0.4300069	0.71389672	0.968139275
hsa-miR-517a-3p	-1.446160246	0.718691721	0.968139275
hsa-miR-517b-3p	-1.446160246	0.718691721	0.968139275
hsa-miR-215-5p	1.22761141	0.721747878	0.968139275
hsa-miR-4508	-1.2778388	0.722754293	0.968139275
hsa-miR-484	0.45415228	0.725800176	0.968139275
hsa-miR-374b-5p	1.109508208	0.727998926	0.968139275
hsa-miR-320e	-1.281140445	0.736566005	0.968139275
hsa-miR-200b-3p	-1.206664869	0.738447595	0.968139275
hsa-miR-125b-5p	0.111204902	0.739045129	0.968139275
hsa-miR-2110	-0.29841254	0.742885799	0.968139275
hsa-miR-22-5p	0.221728821	0.743277797	0.968139275
hsa-miR-92b-5p	0.583847808	0.74678183	0.968139275
hsa-miR-381-3p	0.167118966	0.748141281	0.968139275
hsa-miR-4324	-0.987395706	0.748475236	0.968139275
hsa-miR-18a-3p	-0.575074559	0.753647616	0.968139275
hsa-miR-145-3p	1.187744337	0.755024051	0.968139275
hsa-miR-216b-5p	1.187744337	0.755024051	0.968139275
hsa-miR-369-3p	1.187701449	0.755376467	0.968139275
hsa-miR-664a-5p	-0.504481678	0.760465725	0.968139275
hsa-miR-339-5p	-0.423696196	0.763021477	0.968139275
hsa-miR-1290	-0.206456813	0.763666227	0.968139275
hsa-miR-1271-5p	0.797072312	0.76426517	0.968139275
hsa-miR-548am-3p	0.683646699	0.765114074	0.968139275
hsa-let-7f-1-3p	0.835109281	0.767101501	0.968139275
hsa-miR-483-3p	-0.584510134	0.771065851	0.968139275
hsa-miR-106b-3p	0.235575602	0.775129432	0.968139275
hsa-miR-1301-3p	-0.460968631	0.776221707	0.968139275
hsa-miR-138-5p	-1.106626012	0.784297217	0.968139275
hsa-miR-1298-5p	-1.020576636	0.787644597	0.968139275
hsa-miR-375-3p	-1.020576636	0.787644597	0.968139275
hsa-miR-99a-5p	0.06087429	0.78904279	0.968139275
hsa-miR-1908-5p	-0.622771244	0.790320445	0.968139275
hsa-miR-576-3p	0.441349591	0.790719546	0.968139275
hsa-miR-382-5p	0.155016769	0.79635756	0.968139275
hsa-miR-192-5p	-0.175320098	0.797266426	0.968139275
hsa-let-7b-3p	0.579092533	0.797676106	0.968139275

miRNA assay	log2.Fold change	p.value	FDR
hsa-miR-4745-5p	0.800589369	0.802460144	0.968139275
hsa-miR-369-5p	0.48016749	0.8065949	0.968139275
hsa-miR-744-5p	-0.18335631	0.807169912	0.968139275
hsa-miR-3150a-5p	0.773805487	0.808448309	0.968139275
hsa-miR-455-3p	0.909159233	0.809078487	0.968139275
hsa-miR-150-5p	-0.615357962	0.814257249	0.968139275
hsa-miR-27a-5p	0.132661741	0.816609545	0.968139275
hsa-miR-29c-3p	-0.933373863	0.817931252	0.968139275
hsa-miR-145-5p	-0.933373863	0.817931252	0.968139275
hsa-miR-345-5p	0.673256036	0.853266889	0.968139275
hsa-miR-1193	0.44517196	0.860696511	0.968139275
hsa-miR-24-3p	0.061376465	0.861694385	0.968139275
hsa-miR-214-3p	0.522600005	0.863651141	0.968139275
hsa-miR-450a-5p	-0.498214656	0.864061945	0.968139275
hsa-miR-1180-3p	-0.076599967	0.87085326	0.968139275
hsa-miR-424-5p	0.596250617	0.875909402	0.968139275
hsa-miR-452-5p	0.223529565	0.876098436	0.968139275
hsa-miR-4497	0.556324525	0.88229597	0.968139275
hsa-miR-338-5p	-0.231710983	0.882751145	0.968139275
hsa-miR-494-3p	0.253988593	0.886012997	0.968139275
hsa-miR-182-5p	-0.105133339	0.886796297	0.968139275
hsa-miR-1304-5p	-0.408668756	0.893048722	0.968139275
hsa-miR-7-5p	-0.040327126	0.896761903	0.968139275
hsa-miR-330-5p	-0.284298083	0.896836767	0.968139275
hsa-miR-433-3p	-0.198489851	0.898431966	0.968139275
hsa-miR-30c-2-3p	0.421180176	0.904619313	0.968139275
hsa-miR-122-5p	-0.076632587	0.906250637	0.968139275
hsa-miR-338-3p	-0.40529321	0.907310344	0.968139275
hsa-miR-488-3p	-0.373959057	0.91238487	0.968139275
hsa-miR-139-5p	0.184296084	0.91508506	0.968139275
hsa-miR-877-5p	-0.236459056	0.918935372	0.968139275
hsa-miR-1306-3p	-0.239897207	0.919007723	0.968139275
hsa-miR-1260b	-0.339628668	0.923299234	0.968139275
hsa-miR-99b-5p	0.034321519	0.924787148	0.968139275
hsa-miR-758-3p	0.30639056	0.926164203	0.968139275
hsa-miR-4326	0.146251652	0.926755354	0.968139275
hsa-miR-425-3p	-0.207706683	0.93093488	0.968139275
hsa-miR-3176	-0.317652491	0.933607488	0.968139275
hsa-miR-2682-5p	-0.156296289	0.933689326	0.968139275
hsa-miR-760	-0.091250204	0.938680584	0.968139275
hsa-miR-4664-3p	0.184906583	0.938866286	0.968139275
hsa-miR-10b-3p	-0.288399366	0.94002742	0.968139275
hsa-miR-185-5p	0.088811699	0.940225675	0.968139275
hsa-miR-129-1-3p	-0.24974513	0.951144503	0.968139275
hsa-miR-490-3p	-0.24974513	0.951144503	0.968139275
hsa-miR-873-5p	-0.24974513	0.951144503	0.968139275
hsa-miR-127-5p	-0.24974513	0.951144503	0.968139275

miRNA assay	log2.Fold change	p.value	FDR
hsa-miR-6516-5p	-0.24974513	0.951144503	0.968139275
hsa-miR-181c-5p	-0.24974513	0.951144503	0.968139275
hsa-miR-548s	-0.24974513	0.951144503	0.968139275
hsa-miR-30d-3p	-0.24974513	0.951144503	0.968139275
hsa-miR-187-3p	-0.24974513	0.951144503	0.968139275
hsa-miR-592	-0.24974513	0.951144503	0.968139275
hsa-miR-126-5p	-0.24974513	0.951144503	0.968139275
hsa-miR-363-3p	-0.24974513	0.951144503	0.968139275
hsa-miR-3127-5p	-0.24974513	0.951144503	0.968139275
hsa-miR-24-1-5p	-0.24974513	0.951144503	0.968139275
hsa-miR-490-5p	-0.24974513	0.951144503	0.968139275
hsa-miR-34b-3p	-0.24974513	0.951144503	0.968139275
hsa-miR-181c-3p	-0.24974513	0.951144503	0.968139275
hsa-miR-410-3p	-0.24974513	0.951144503	0.968139275
hsa-miR-29b-2-5p	-0.249745154	0.951195984	0.968139275
hsa-miR-214-5p	-0.249745154	0.951195984	0.968139275
hsa-miR-1323	-0.249745154	0.951195984	0.968139275
hsa-miR-517c-3p	-0.249745154	0.951195984	0.968139275
hsa-miR-3916	-0.249745154	0.951195984	0.968139275
hsa-miR-378c	-0.249745154	0.951195984	0.968139275
hsa-miR-302d-3p	-0.249745154	0.951195984	0.968139275
hsa-miR-6765-3p	-0.249745154	0.951195984	0.968139275
hsa-miR-200a-3p	-0.249745154	0.951195984	0.968139275
hsa-miR-363-5p	-0.249745154	0.951195984	0.968139275
hsa-miR-497-5p	-0.249745154	0.951195984	0.968139275
hsa-miR-371a-5p	-0.249745154	0.951195984	0.968139275
hsa-miR-200c-3p	-0.249745154	0.951195984	0.968139275
hsa-miR-29b-1-5p	-0.22754117	0.951732678	0.968139275
hsa-miR-1307-3p	-0.031239446	0.952205454	0.968139275
hsa-miR-1285-3p	0.218531796	0.953584774	0.968139275
hsa-miR-30b-5p	0.158866189	0.955372603	0.968139275
hsa-miR-125b-2-3p	0.167112406	0.961864663	0.970884221
hsa-miR-24-2-5p	0.08373391	0.963188365	0.970884221
hsa-miR-320b	-0.023373686	0.964482787	0.970884221
hsa-miR-383-5p	0.123157663	0.972029748	0.97632127

Table S2 Six selected miRNAs upregulated by SOX17 and reduced in patients with IPH and hypoxic mice models of PH.

	miRNA	Accession in miRBase
1	miR-361-3p	h MIMAT0004682/m MIMAT0017075
2	miR-221-3p	h MIMAT0000278/m MIMAT0000669
3	miR-654-3p	h MIMAT0004814/m MIMAT0004898
4	miR-224-5p	h MIMAT0000281/m MIMAT0000671
5	miR-574-3p	h MIMAT0003239/m MIMAT0004894
6	miR-503-3p	h MIMAT0022925/m MIMAT0004790

Table S3 Kyoto Encyclopedia of Genes and Genomes (KEGG) pathway associations of miR-224-5p and miR-361-3p target genes.

	Term	Pathway ID	*P*-value	Gene Names
1	Cytokine-cytokine receptor interaction	hsa04060	1.63E-11	CSF3/CXCL1/CXCL5/GDF15/IL1A/IL1B/IL6/IL7R/TNFRSF10B/FAS/CXCL2/CXCL3/CXCL8/CXCL6/INHBA/CSF1/CSF2/CCL5/IL32/TNFRSF10C/EBI3/TNFRSF14/IL11/CD70/EDA2R/TNFRSF9/TNFSF9/CCL20/CCL2/IL24/IL12RB1/IL1RL1/TNFRSF25/TNFSF10/NGFR/IL7/IL2RB/TNFSF4/ACVRL1/CCR7/GHR/CCR4/CXCL11/CCL3/CXCR2/IL22RA1/CX3CL1/TNFSF14/IL2RG/IL18/IL16/BMP8A/EDAR/CXCL12/GDF9/IFNL1/TNFSF15/CXCL10/NODAL/IL23A/IFNB1/PRLR/IL20RB/TNFSF13B/TNF/TNFSF11/CSF2RB/TNFRSF18/TNFRSF8/BMP2/IL33
2	Rheumatoid arthritis	hsa05323	2.88E-11	CXCL1/CXCL5/ICAM1/IL1A/IL1B/IL6/CXCL2/CXCL3/CXCL8/CXCL6/CSF1/CSF2/CCL5/IL11/CCL20/CCL2/CCL3/MMP1/TLR2/FOS/CTSK/IL18/HLA-DMA/ATP6V0A4/HLA-DOB/HLA-DOA/CXCL12/HLA-DRB1/IL23A/HLA-DQB1/TNFSF13B/TNF/TNFSF11/ATP6V1G2
3	TNF signaling pathway	hsa04668	1.19E-10	CXCL1/CXCL5/ICAM1/IL1B/IL6/TNFAIP3/FAS/CXCL2/PTGS2/BIRC3/CXCL3/CXCL6/CFLAR/NFKBIA/CSF1/CSF2/CCL5/TRAF1/IRF1/DAB2IP/CASP10/MAPK11/VCAM1/JUNB/MAPK13/CCL20/CCL2/MAP3K8/EDN1/SELE/CX3CL1/FOS/CXCL10/IFNB1/MMP9/NOD2/TNF
4	Viral protein interaction with cytokine and cytokine receptor	hsa04061	2.14E-08	CXCL1/CXCL5/IL6/TNFRSF10B/CXCL2/CXCL3/CXCL8/CXCL6/CSF1/CCL5/TNFRSF10C/TNFRSF14/CCL20/CCL2/IL24/TNFSF10/IL2RB/CCR7/CCR4/CXCL11/CCL3/CXCR2/IL22RA1/CX3CL1/TNFSF14/IL2RG/IL18/CXCL12/CXCL10/IL20RB/TNF
5	p53 signaling pathway	hsa04115	5.59E-08	CDKN1A/MDM2/PMAIP1/TP53I3/CD82/TNFRSF10B/GADD45A/FAS/PIDD1/DDB2/ZMAT3/SESN2/RRM2B/SESN1/CCNG1/APAF1/PERP/BBC3/PPM1D/STEAP3/SFN/IGFBP3/SERPINB5/ADGRB1/CCND2
6	NF-κB signaling pathway	hsa04064	5.94E-08	CXCL1/ICAM1/IL1B/TNFAIP3/GADD45A/CXCL2/PTGS2/BIRC3/PIDD1/CXCL3/CXCL8/CFLAR/NFKBIA/DDX58/TRAF1/NFKB2/RELB/PLCG2/VCAM1/EDA2R/BCL2A1/BLNK/TNFSF14/EDAR/CXCL12/CARD14/TNFSF13B/TNF/TNFSF11/LAT/CARD11
7	Cell adhesion molecules	hsa04514	6.36E-08	ICAM1/SDC4/CLDN1/VCAN/HLA-B/F11R/CLDN11/SDC1/ICOSLG/NRCAM/CDH5/VCAM1/PDCD1LG2/L1CAM/HLA-

				F/ICAM2/CDH3/SELE/SELPLG/CDH4/CD34/ITGB8/ESAM/ITGAM/CNTN2/HLA-DMA/HLA-DOB/CLDN14/HLA-DOA/HLA-DRB1/CLDN23/HLA-DQB1/NLGN4X/CLDN16/NFASC/SDC2/CDH15/CLDN4/SELP
8	Malaria	hsa05144	3.46E-07	CSF3/ICAM1/IL1B/IL6/LRP1/CXCL8/SDC1/VCAM1/CCL2/THBS2/SELE/HGF/TLR2/IL18/CD36/KLRC4-KLRK1/TNF/SDC2/SELP
9	Lipid and atherosclerosis	hsa05417	4.09E-07	CXCL1/ICAM1/IL1B/IL6/SOD2/TNFRSF10B/FAS/CXCL2/CXCL3/CXCL8/NFKBIA/POU2F2/CCL5/LDLR/HSPA8/APAF1/HSP90AA1/MIB2/DDIT3/PPARG/CASP1/MAPK11/PLCB4/VCAM1/ERN1/MAPK13/NCF2/CCL2/CYP1A1/TNFSF10/PYCARD/SELE/CCL3/OLR1/MMP1/NLRP3/TLR2/CAMK2B/FOS/IL18/HSPA6/IFNB1/MMP9/CD36/TNF/RXRG/CAMK2A/SELP
10	Pertussis	hsa05133	5.76E-07	C3/CXCL5/IL1A/IL1B/IL6/CXCL8/CXCL6/C1R/IRF1/CASP1/MAPK11/C1S/MAPK13/C5/PYCARD/SERPING1/NLRP3/C4B/C2/FOS/C4A/ITGAM/IL23A/TNF
11	IL-17 signaling pathway	hsa04657	9.61E-07	CSF3/CXCL1/CXCL5/IL1B/IL6/TNFAIP3/CXCL2/PTGS2/CXCL3/CXCL8/CXCL6/NFKBIA/CSF2/TRAF4/HSP90AA1/MAPK11/MAPK13/CCL20/CCL2/FOSB/MMP1/FOS/MUC5AC/CXCL10/MMP9/MMP13/TNF
12	Complement and coagulation cascades	hsa04610	1.49E-06	C3/PLAT/SERPINB2/F2RL2/C1R/CFB/CFH/C1S/THBD/SERPINA1/F8/C5/SERPIND1/SERPING1/VWF/C4B/C2/C4A/ITGAM/CFD/C5AR1/FGB/VTN/ITGAX/FGG
13	Legionellosis	hsa05134	3.51E-06	C3/CXCL1/IL1B/IL6/CXCL2/CXCL3/CXCL8/NFKBIA/HSPA8/APAF1/NFKB2/CASP1/EEF1A2/PYCARD/TLR2/ITGAM/IL18/HSPA6/TNF
14	Staphylococcus aureus infection	hsa05150	5.09E-06	C3/ICAM1/KRT17/C1R/CFB/CFH/PTAFR/C1S/C5/SELPLG/C4B/C2/KRT14/C4A/ITGAM/HLA-DMA/HLA-DOB/HLA-DOA/CFD/HLA-DRB1/HLA-DQB1/C5AR1/KRT19/FPR2/FGG/SELP
15	ECM-receptor interaction	hsa04512	1.03E-05	LAMB3/SDC4/COL4A5/COL4A1/AGRN/LAMB2/SDC1/COL9A3/ITGB3/COL4A6/HSPG2/COL9A2/VWF/THBS2/RELN/LAMC3/COL4A4/ITGA11/ITGB8/TNXB/GP1BB/COL2A1/CD36/VTN
16	Influenza A	hsa05164	2.12E-05	ICAM1/IL1A/IL1B/IL6/MX1/OAS2/TNFRSF10B/FAS/OAS3/CXCL8/NFKBIA/DDX58/CCL5/IFIH1/OAS1/APAF1/CASP1/IRF9/MX2/RSAD2/CCL2/TLR3/TNFSF10/PYCARD/CIITA/NLRP3/IL18/HLA-DMA/HLA-DOB/HLA-

				DOA/HLA-DRB1/CXCL10/HLA-DQB1/IFNB1/TNF/IL33/NXF3
17	Coronavirus disease - COVID-19	hsa05171	4.52E-05	C3/CSF3/IL1B/IL6/ISG15/MX1/OAS2/OAS3/CXCL8/NFKBIA/DDX58/CSF2/IFIH1/NRP1/OAS1/C1R/CFB/CASP1/IRF9/MX2/MAPK11/PLCG2/C1S/MAPK13/CCL2/TLR3/C5/VWF/MMP1/NLRP3/TLR2/RPL22L1/C4B/C2/FOS/C4A/CFD/CXCL10/IFNB1/C5AR1/ACE2/FGB/TNF/FGG/SELP
18	Graft-versus-host disease	hsa05332	6.77E-05	IL1A/IL1B/IL6/FAS/HLA-B/HLA-F/PRF1/HLA-DMA/HLA-DOB/HLA-DOA/HLA-DRB1/KLRC1/HLA-DQB1/TNF
19	Apoptosis	hsa04210	7.73E-05	PMAIP1/TNFRSF10B/GADD45A/FAS/BIRC3/PIDD1/CFLAR/NFKBIA/TRAF1/APAF1/LMNB1/BBC3/DDIT3/DAB2IP/CTSS/CASP10/PARP3/ERN1/CTSF/BCL2A1/TNFSF10/DFFB/FOS/CTSK/PRF1/CTSO/NTRK1/TNF/CSF2RB/TUBA3E
20	AGE-RAGE signaling pathway in diabetic complications	hsa04933	0.000271446	ICAM1/IL1A/IL1B/IL6/COL4A5/CXCL8/COL4A1/MAPK11/PLCG2/PLCB4/THBD/VCAM1/COL4A6/MAPK13/PLCD1/CCL2/COL3A1/PRKCZ/EDN1/SELE/COL4A4/AGT/TNF
21	Type I diabetes mellitus	hsa04940	0.000368922	IL1A/IL1B/FAS/HLA-B/CPE/HLA-F/PRF1/HLA-DMA/HLA-DOB/HLA-DOA/HLA-DRB1/HLA-DQB1/TNF
22	Protein digestion and absorption	hsa04974	0.000427958	COL7A1/COL4A5/COL4A1/COL17A1/COL9A3/COL27A1/COL4A6/COL9A2/COL5A3/COL3A1/KCNN4/ATP1B2/COL4A4/SLC7A8/KCNQ1/COL2A1/COL15A1/ACE2/SLC8A2/COL11A1/SLC7A7/FXYD2/SLC8A3
23	NOD-like receptor signaling pathway	hsa04621	0.000455052	CXCL1/IL1B/IL6/OAS2/TNFAIP3/CXCL2/BIRC3/CXCL3/OAS3/CXCL8/NLRP1/NFKBIA/TXNIP/CCL5/OAS1/HSP90AA1/GABARAPL1/CASP1/IRF9/MAPK11/PLCB4/MAP1LC3A/MAPK13/CCL2/PYCARD/GBP4/NLRP3/GBP5/IL18/AIM2/GBP2/IFNB1/MAP1LC3B2/NOD2/TNF
24	C-type lectin receptor signaling pathway	hsa04625	0.000495338	IL1B/IL6/MDM2/PTGS2/NFKBIA/NFKB2/IRF1/PLK3/RELB/CASP1/IRF9/MAPK11/PLCG2/MRAS/MAPK13/PYCARD/NLRP3/EGR2/LSP1/IL23A/TNF/EGR3/CD209
25	ABC transporters	hsa02010	0.000601617	ABCA13/TAP1/ABCA7/ABCD1/ABCA2/ABCA12/ABCG2/ABCA8/ABCG4/ABCC9/ABCC6/ABCA4/ABCC11
26	Hematopoietic cell lineage	hsa04640	0.000607455	CSF3/IL1A/IL1B/IL6/IL7R/CSF1/CSF2/KITLG/ITGB3/IL11/IL7/CD34/ITGAM/HLA-DMA/HLA-DOB/HLA-DOA/HLA-DRB1/GP1BB/HLA-DQB1/CD36/TNF/CD33
27	Osteoclast differentiation	hsa04380	0.000874188	IL1A/IL1B/NFKBIA/CSF1/NFKB2/PPARG/RELB/ITGB3/IRF9/MAPK11/PLCG2/JUNB/MAPK13/NCF2/FOSB/TREM2/BLNK/OSCAR/FOS/CTSK/SOC

				S1/SIRPG/IFNB1/TNF/TNFSF11/SIRPB1
28	Amoebiasis	hsa05146	0.000930778	CXCL1/IL1B/IL6/LAMB3/COL4A5/CXCL2/CXCL3/CXCL8/COL4A1/CSF2/LAMB2/PLCB4/COL4A6/COL3A1/MUC2/LAMC3/COL4A4/TLR2/ITGAM/GNA15/GNA14/TNF
29	Leishmaniasis	hsa05140	0.000997143	C3/IL1A/IL1B/PTGS2/NFKBIA/EEF1A2/MAPK11/MAPK13/NCF2/TLR2/FOS/ITGAM/HLA-DMA/HLA-DOB/HLA-DOA/HLA-DRB1/HLA-DQB1/TNF
30	Inflammatory bowel disease	hsa05321	0.001040142	IL1A/IL1B/IL6/STAT4/IL12RB1/TLR2/IL2RG/IL18/HLA-DMA/HLA-DOB/HLA-DOA/HLA-DRB1/IL23A/HLA-DQB1/NOD2/TNF
31	Antigen processing and presentation	hsa04612	0.001169996	HLA-B/TAP1/HSPA8/HSP90AA1/CTSS/IFI30/HLA-F/CIITA/HLA-DMA/HLA-DOB/HLA-DOA/HSPA6/HLA-DRB1/KLRC1/HLA-DQB1/KLRC3/TNF/CD74
32	African trypanosomiasis	hsa05143	0.001206935	ICAM1/IL1B/IL6/FAS/APOL1/PLCB4/VCAM1/SELE/IL18/IDO1/TNF
33	Epstein-Barr virus infection	hsa05169	0.001322614	CDKN1A/ICAM1/IL6/ISG15/MDM2/OAS2/TNFAIP3/GADD45A/FAS/DDB2/OAS3/HLA-B/TAP1/NFKBIA/DDX58/OAS1/APAF1/NFKB2/RELB/IRF9/MAPK11/PLCG2/MAPK13/HLA-F/BLNK/TLR2/JAK3/HLA-DMA/HLA-DOB/HLA-DOA/HLA-DRB1/CXCL10/CCND2/HLA-DQB1/IFNB1/TNF
34	Fluid shear stress and atherosclerosis	hsa05418	0.001423172	ICAM1/IL1A/IL1B/PLAT/SDC4/GPC1/SDC1/HSP90AA1/ITGB3/MAPK11/THBD/CDH5/VCAM1/ASS1/MAPK13/NCF2/CCL2/GSTA4/PRKCZ/EDN1/GSTM2/SELE/FOS/NPPC/MMP9/TNF/SDC2
35	PI3K-Akt signaling pathway	hsa04151	0.002505136	CDKN1A/CSF3/FGF2/IL6/IL7R/LAMB3/MDM2/AREG/COL4A5/COL4A1/DDIT4/CSF1/EREG/LAMB2/KITLG/EPHA2/HSP90AA1/COL9A3/ITGB3/NR4A1/PIK3AP1/PGF/EFNA1/COL4A6/COL9A2/GNGT2/FGFR3/ANGPT4/NGFR/IL7/IL2RB/VWF/THBS2/RELN/LAMC3/GHR/COL4A4/HGF/ITGA11/TLR2/ITGB8/JAK3/GNB3/NTF4/PDGFRA/IL2RG/TNXB/ANGPT2/CCND2/COL2A1/IFNB1/PRLR/NTRK1/VTN/NTRK2
36	Measles	hsa05162	0.002998214	IL1A/IL1B/IL6/MX1/OAS2/TNFAIP3/FAS/OAS3/NFKBIA/DDX58/IFIH1/OAS1/HSPA8/APAF1/BBC3/IRF9/MX2/IL2RB/TLR2/JAK3/FOS/IL2RG/HSPA6/CCND2/IFNB1/CD209
37	Tuberculosis	hsa05152	0.004728741	C3/IL1A/IL1B/IL6/APAF1/PLK3/IRAK2/CTSS/CASP10/MAPK11/MAPK13/CIITA/TLR2/LSP1/CAMK2B/ITGAM

				/IL18/HLA-DMA/ATP6V0A4/HLA-DOB/HLA-DOA/HLA-DRB1/IL23A/HLA-DQB1/IFNB1/NOD2/TNF/ITGAX/CAMK2A/CD209/CD74
38	MAPK signaling pathway	hsa04010	0.004777679	FGF2/IL1A/IL1B/AREG/GADD45A/FAS/DUSP5/CSF1/ARRB1/EREG/HSPA8/KITLG/EPHA2/NFKB2/DDIT3/RELB/NR4A1/MAPK11/PGF/ECSIT/EFNA1/MRAS/MAPK13/PLA2G4C/MAP3K8/CACNA2D4/FGFR3/ANGPT4/NGFR/RASGRF1/HGF/FOS/NTF4/PDGFRA/CACNA1A/CACNA1G/HSPA6/MAPT/CACNB4/DUSP9/ANGPT2/CACNA2D2/NTRK1/TNF/NTRK2/PTPN7
39	Allograft rejection	hsa05330	0.005323199	FAS/HLA-B/HLA-F/PRF1/HLA-DMA/HLA-DOB/HLA-DOA/HLA-DRB1/HLA-DQB1/TNF
40	Human papillomavirus infection	hsa05165	0.00675044	CDKN1A/ISG15/LAMB3/MDM2/MX1/OASL/COL4A5/FAS/PTGS2/HLA-B/COL4A1/LAMB2/IRF1/NOTCH3/COL9A3/ITGB3/IRF9/PARD6G/MX2/HES2/COL4A6/HES4/COL9A2/NOTCH4/HLA-F/APC2/WNT9A/PRKCZ/TLR3/TCF7L1/VWF/THBS2/RELN/LAMC3/HES6/COL4A4/ITGA11/WNT4/ITGB8/ATP6V0A4/TNXB/HEY1/CCND2/COL2A1/IFNB1/WNT7B/TNF/ATP6V1G2/VTN/HES5
41	Toxoplasmosis	hsa05145	0.007017466	LAMB3/BIRC3/NFKBIA/LDLR/HSPA8/LAMB2/MAPK11/MAPK13/ALOX5/CIITA/LAMC3/TLR2/SOCS1/HLA-DMA/HLA-DOB/HLA-DOA/HSPA6/HLA-DRB1/HLA-DQB1/TNF/IRGM
42	Th1 and Th2 cell differentiation	hsa04658	0.00774295	NFKBIA/JAG2/NOTCH3/MAPK11/MAPK13/STAT4/IL12RB1/IL2RB/JAK3/DLL4/FOS/IL2RG/HLA-DMA/HLA-DOB/HLA-DOA/HLA-DRB1/HLA-DQB1/LAT
43	Small cell lung cancer	hsa05222	0.00774295	CDKN1A/LAMB3/GADD45A/COL4A5/PTGS2/BIRC3/DDB2/COL4A1/NFKBIA/LAMB2/TRAF1/APAF1/TRAF4/COL4A6/CKS2/LAMC3/COL4A4/RXRG
44	Th17 cell differentiation	hsa04659	0.009624586	IL1B/IL6/NFKBIA/EBI3/HSP90AA1/MAPK11/MAPK13/IL12RB1/IL2RB/JAK3/FOS/IL2RG/HLA-DMA/HLA-DOB/HLA-DOA/HLA-DRB1/IL23A/HLA-DQB1/LAT/RXRG
45	Axon guidance	hsa04360	0.010091538	EFNB1/PLXNB3/NRP1/MYL9/EPHA2/NTN1/RHOD/PLXNA3/PLXNB1/SEMA3F/PARD6G/SEMA3B/PLCG2/ABLIM1/EFNA1/TRPC1/RND1/SRGAP3/L1CAM/PAK6/UNC5B/PRKCZ/BUB1B-PAK6/WNT4/CAMK2B/SEMA3G/CXCL12/EPHB3/RGMA/CAMK2A

46	Chagas disease	hsa05142	0.010755728	C3/IL1B/IL6/FAS/CXCL8/CFLAR/NFKBIA/CCL5/MAPK11/PLCB4/MAPK13/CCL2/CCL3/TLR2/FOS/GNA15/GNA14/IFNB1/TNF
47	Transcriptional misregulation in cancer	hsa05202	0.012054652	CDKN1A/IL6/MDM2/NFKBIZ/PLAT/GADD45A/BIRC3/DDB2/CXCL8/CSF2/TRAF1/DDIT3/PPARG/ETV1/NUPR1/IGFBP3/NR4A3/PBX1/ETV7/SPINT1/LYL1/BCL2A1/ID2/NGFR/IL2RB/PAX5/ITGAM/CCND2/MMP9/NTRK1/RXRG
48	Leukocyte transendothelial migration	hsa04670	0.017108479	ICAM1/CLDN1/F11R/MYL9/CLDN11/MAPK11/PLCG2/CDH5/VCAM1/PTK2B/MAPK13/NCF2/ESAM/ITGAM/CLDN14/CXCL12/CLDN23/MMP9/CLDN16/CLDN4
49	Relaxin signaling pathway	hsa04926	0.017512114	COL4A5/ACTA2/COL4A1/NFKBIA/ARRB1/MAPK11/PLCB4/COL4A6/MAPK13/GNGT2/COL3A1/PRKCZ/EDN1/SHC4/MMP1/COL4A4/FOS/GNB3/GNA15/MMP9/MMP13/RXFP1
50	Autoimmune thyroid disease	hsa05320	0.022180879	FAS/HLA-B/HLA-F/TG/PRF1/HLA-DMA/HLA-DOB/HLA-DOA/HLA-DRB1/TPO/HLA-DQB1
51	Arachidonic acid metabolism	hsa00590	0.025793375	PTGS2/GGT1/GPX3/PLA2G4C/CYP4F2/CYP4F3/ALOX5/PTGS1/PLA2G6/ALOX12B/PLB1/PTGIS
52	Hepatitis C	hsa05160	0.026344999	CDKN1A/MX1/OAS2/CLDN1/FAS/OAS3/CFLAR/NFKBIA/IFIT1/DDX58/LDLR/OAS1/CLDN11/APAF1/IRF9/MX2/RSAD2/TLR3/CLDN14/CXCL10/CLDN23/IFNB1/CLDN16/TNF/CLDN4
53	Neuroactive ligand-receptor interaction	hsa04080	0.026618145	C3/ADM/F2RL2/LTB4R/GRIN3B/PTAFR/LTB4R2/PTGER2/ADRB2/MC1R/UCN2/EDNRA/EDN1/C5/PTGER1/GABBR2/GHR/P2RY6/CHRM4/CALCRL/GRM2/HTR4/GABRQ/EDN2/OPRL1/GABRB1/MLNR/AGT/KISS1/PPY/PTGIR/C5AR1/NPY1R/CHRNA6/PRLR/GALR2/CHRNA9/GLRA3/CHRNA3/TAC3/FPR2/PDYN/RXFP1/GPR35/SSTR1/CHRNB2/S1PR5/ADRA1D
54	PPAR signaling pathway	hsa03320	0.0286781	ANGPTL4/SCD/PLTP/GK/PPARG/SCD5/ACSL5/PLIN4/OLR1/MMP1/PLIN5/CD36/RXRG/SLC27A2
55	Epithelial cell signaling in Helicobacter pylori infection	hsa05120	0.032239378	CXCL1/CXCL2/CXCL3/F11R/CXCL8/NFKBIA/CCL5/MAPK11/PLCG2/MAPK13/CXCR2/ATP6V0A4/ATP6V1G2
56	Cytosolic DNA-sensing pathway	hsa04623	0.032471749	IL1B/IL6/NFKBIA/DDX58/CCL5/CASP1/PYCARD/IL18/AIM2/CXCL10/IFNB1/IL33
57	JAK-STAT signaling pathway	hsa04630	0.036946965	CDKN1A/CSF3/IL6/IL7R/CSF2/IRF9/IL11/STAT4/IL24/IL12RB1/IL7/IL2RB/GHR/IL22RA1/JAK3/SOCS1/PDGFRA/IL2RG/IFNL1/CCND2/IL23A/IFNB1/PRLR/IL20RB/CSF2RB
58	Natural killer cell mediated cytotoxicity	hsa04650	0.036978049	ICAM1/TNFRSF10B/FAS/HLA-B/CSF2/PLCG2/ULBP1/PTK2B/ICA

				M2/TNFSF10/SHC4/RAET1G/PRF1/RAET1L/KLRC1/SH2D1B/IFNB1/KLRC3/KLRC4-KLRK1/TNF/LAT
59	Kaposi sarcoma-associated herpesvirus infection	hsa05167	0.037940088	C3/CDKN1A/CXCL1/FGF2/ICAM1/IL6/FAS/CXCL2/PTGS2/CXCL3/HLA-B/CXCL8/NFKBIA/CSF2/IRF9/MAPK11/PLCG2/MAP1LC3A/MAPK13/GNGT2/HLA-F/TLR3/TCF7L1/CCR4/FOS/GNB3/ANGPT2/IFNB1/MAP1LC3B2
60	Thyroid cancer	hsa05216	0.038218488	CDKN1A/GADD45A/DDB2/PPARG/TCF7L1/RET/NTRK1/RXRG
61	cAMP signaling pathway	hsa04024	0.040126067	PDE4C/NFKBIA/SLC9A1/MYL9/HCN2/GRIN3B/HCAR3/PTGER2/ADRB2/HCAR2/SOX9/EDNRA/EDN1/GABBR2/ATP1B2/CNGB1/CAMK2B/FOS/HTR4/EDN2/ATP2B2/PDE10A/PDE3A/NPY1R/RYR2/HCN4/CNGA1/ATP2B3/ADCY10/SSTR1/CAMK2A/FXYD2
62	Primary immunodeficiency	hsa05340	0.044058848	IL7R/TAP1/RAG1/BLNK/CIITA/JAK3/IL2RG/CD79A
63	Proteoglycans in cancer	hsa05205	0.044996925	CDKN1A/FGF2/MDM2/SDC4/GPC1/FAS/TIMP3/SLC9A1/SDC1/ANK1/ITGB3/MAPK11/PLCG2/MRAS/HSPG2/MAPK13/TFAP4/WNT9A/DCN/HGF/WNT4/TLR2/CAMK2B/LUM/MMP9/WNT7B/TNF/SDC2/VTN/CAMK2A
64	Toll-like receptor signaling pathway	hsa04620	0.048325173	IL1B/IL6/CXCL8/NFKBIA/CCL5/MAPK11/MAPK13/MAP3K8/TLR3/CXCL11/CCL3/TLR2/FOS/CTSK/CXCL10/IFNB1/TNF
65	Hypertrophic cardiomyopathy	hsa05410	0.052944698	IL6/ITGB3/PRKAB1/CACNA2D4/EDN1/ITGA11/ITGB8/DES/CACNB4/AGT/RYR2/SLC8A2/CACNA2D2/TNF/SLC8A3
66	Steroid biosynthesis	hsa00100	2.07E-16	MSMO1/FDFT1/SQLE/CYP51A1/LSS/DHCR7/EBP/SC5D/TM7SF2/HSD17B7
67	Terpenoid backbone biosynthesis	hsa00900	4.10E-10	HMGCS1/MVD/HMGCR/ACAT2/FDPS/MVK/IDI1
68	Fatty acid metabolism	hsa01212	8.64E-06	ACAT2/ELOVL6/FADS2/FADS1/SCD/FASN
69	Biosynthesis of unsaturated fatty acids	hsa01040	7.75E-05	ELOVL6/FADS2/FADS1/SCD
70	PPAR signaling pathway	hsa03320	0.004122805	HMGCS1/FADS2/SCD/FABP3
71	TGF-beta signaling pathway	hsa04350	0.00872576	ID1/ID3/INHBE/NOG
72	Hepatitis C	hsa05160	0.011334378	MX1/OAS2/OAS1/PPP2R2C/MX2
73	Cytokine-cytokine receptor interaction	hsa04060	0.013404959	IL21R/TNFSF9/CXCL5/GDF15/IL7R/TNFSF18/INHBE

74	Glycerolipid metabolism	hsa00561	0.01559606	LPIN1/LIPG/AKR1B10
75	Influenza A	hsa05164	0.016294379	FDPS/MX1/OAS2/OAS1/MX2
76	AMPK signaling pathway	hsa04152	0.019893856	HMGCR/SCD/FASN/PPP2R2C
77	Butanoate metabolism	hsa00650	0.024116965	HMGCS1/ACAT2
78	Glyoxylate and dicarboxylate metabolism	hsa00630	0.027448645	ACSS2/ACAT2
79	Measles	hsa05162	0.031946467	MX1/OAS2/OAS1/MX2
80	Coronavirus disease - COVID-19	hsa05171	0.049850481	MX1/OAS2/VWF/OAS1/MX2

Table S4 The intersection genes of in silico predicted target genes and RNASeq-validated target genes of miR-224-5p and miR-361-3p associated with pathways regulating cell proliferation, inflammation, and apoptosis.

**miR-224-5p**		
**Gene**	**Name**	**Function**
1 IL6	interleukin 6	cell migration/proliferation/inflammation/ angiogenesis
2 MMP12/13	matrix metallopeptidase 12/13	cell proliferation/inflammation/vascular remodeling/ angiogenesis
3 ICAM1/4	intercellular adhesion molecule 1	cell migration/proliferation/inflammation/ angiogenesis
4 FI44L	interferon induced protein 44 like	cell migration/proliferation/inflammation/ angiogenesis
5 PCYT2	phosphate cytidylytransferase 2 , ethanolamine	cell apoptosis
6 CCL2	C-C motif chemokine ligand 2	cell migration/proliferation/inflammation/apoptosis/ angiogenesis
7 FASN	fatty acid synthase	cell migration/proliferation/inflammation/apoptosis/ remodeling/angiogenesis
8 TCN2	transcobalamin 2	cell migration/proliferation
9 OLR1	oxidized low density lipoprotein receptor 1	cell migration/proliferation/inflammation/vascular remodeling
10 ITM2B	integral membrane protein 2B	cell proliferation/apoptosis/vascular
11 RNF144B	ring finger protein 144B	cell migration/proliferation/inflammation/apoptosis
12 EGR2	early growth response 2	cell migration/proliferation/inflammation/apoptosis/ remodeling
13 RIOK3	RIO kinase 3	cell migration/proliferation/inflammation/apoptosis
14 NR4A3	nuclear receptor subfamily 4 group A member 3	cell migration/proliferation/inflammation/apoptosis/ remodeling/angiogenesis
15 KLLN	killin , p53 regulated DNA replication inhibitor	cell proliferation/apoptosis
16 vWF	von Willebrand factor	cell proliferation/inflammation/apoptosis/ vascular remodeling/angiogenesis

**miR-361-3p**		
**Gene**	**Name**	**Function**
1 APLN	apelin	cell migration/proliferation/inflammation/apoptosis/ vascular remodeling/angiogenesis
2 IL21R	interleukin 21 receptor	cell proliferation/inflammation/apoptosis/ vascular remodeling/angiogenesis
3 MAP3K7L	MAP3K7 C-terminal like	vascular remodeling
4 MVK	mevalonate kinase	proliferation/inflammation/apoptosis/vascular remodeling
5 PDGFB	platelet derived growth factor subunit B	cell migration/vascular angiogenesis
6 TNFSF18	TNF superfamily member 18	cell migration/proliferation/apoptosis/ vascular angiogenesis
7 DAAM2	dishevelled associated activator of morphogenesis 2	cell migration/proliferation/remodeling
8 LSS	lanosterol synthase	cell migration/proliferation/inflammation/apoptosis/ vascular remodeling
9 ORAI3	ORAI calcium release-activated calcium modulator 3	cell migration/proliferation/inflammation/apoptosis/ vascular remodeling/angiogenesis
10 ELOVL6	ELOVL fatty acid elongase 6	cell proliferation/inflammation/apoptosis/vascular remodeling
11 SLC16A6	solute carrier family 16-member 6	cell proliferation/remodeling
12 LIPG	lipse G , endothelial type	cell proliferation/inflammation/vascular remodeling/angiogenesis
13 BMF	Bcl2 modifying factor	cell migration/proliferation/inflammation/apoptosis/ vascular remodeling/angiogenesis
14 SCD	stearoyl-CoA desaturase	cell migration/proliferation/inflammation/apoptosis/ vascular remodeling/angiogenesis
15 ADAMTS14	ADAM metallopeptidase with thrombospondin type 1 motif 14	cell migration/proliferation
16 PSCK9	proprotein convertase subtilisin/kexin type 9	cell migration/proliferation/inflammation
17 NOG	noggin	cell migration/proliferation/inflammation/apoptosis/ remodeling/angiogenesis
18 TSC22D3	TSC22 domain family member 3	cell proliferation/apoptosis
19 BDKRB2	bradykinin receptor B2	cell migration/proliferation/inflammation/apoptosis/ vascular remodeling/angiogenesis

Table S5 Demographics of blood donors, IPH patients and healthy volunteers. Data represented as median (range), Control (n=14), IPH (n=12).

		Control	IPH
Male/Females		4 per 14	2 per 12
Age (years)		36.0 (23.0 - 48.0)	38.33 (27.0 - 51.0)
Time from diagnosis (months)		-	13.0 (0.1 - 60.0)
mPAP (mmHg)			58.0 (29 - 85)
WHO Functional Class	I	-	2
	II	-	4
	III	-	5
	IV	-	1
No treatment		-	1
Warfarin		-	3
Statins		-	1
Prostanoids		-	6
PDE5 Inhibitors		-	7
ER Antagonists		-	5
Calcium Antagonists		-	2

PDE5, Phosphodiesterase type 5; ER, Estrogen-Receptor
mPAP, mean Pulmonary Arterial Pressure.

Table S6 Demographics of lung tissue donors including IPH patients (n = 7) and control individuals (n = 7). Data represented as median (range).

	Control	IPH
Male/Females	4 per 7	3 per 7
Age (years)	36.0 (26.0 - 58.0)	40.15 (22.0 - 50.0)
Time from diagnosis (months)	-	36 (24 - 78)
mPAP (mmHg)	-	61.0 (53.0 – 71.0)
No treatment	-	4
ER Antagonists	-	2
Prostanoids	-	1

ER, Estrogen-Receptor

mPAP, mean Pulmonary Arterial Pressure.

Table S7 Primers used in this study

Primers for qRT-PCR	Sequences
h-SOX17 Forward	ATCCTCAGACTCCTGGGTTT
h-SOX17 Reverse	ACTGTTCAAGTGGCAGACAAA
h-NR4A3 Forward	TGCGTCCAAGCCCAATATAGC
h-NR4A3 Reverse	GGTGTATTCCGAGCTGTATGTCT
h-PCSK9 Forward	ATGGTCACCGACTTCGAGAAT
h-PCSK9 Reverse	GTGCCATGACTGTCACACTTG
h-β-actin Forward	GAAATCGTGCGTGACATTAAG
h-β-actin Reverse	GCTAGAAGCATTTGCGGTGGA
m-SOX17 Forward	ACCTACACTTACGCTCCAGTC
m-SOX17 Reverse	GCCGTAGTACAGGTGCAGAG
m-NR4A3 Forward	AGGATTCACTGATCTCCCCAA
m-NR4A3 Reverse	GATGCAGGACAAGTCCATTGC
m-PCSK9 Forward	TCTATGCTTCCTGCTGCCAT
m-PCSK9 Reverse	AGAGAGCCATGCAGGCATAT
m-β-actin Forward	GTGACGTTGACATCCGTAAAGA
m-β-actin Reverse	GCCGGACTCATCGTACTCC

Supplementary Figures

Figure S1

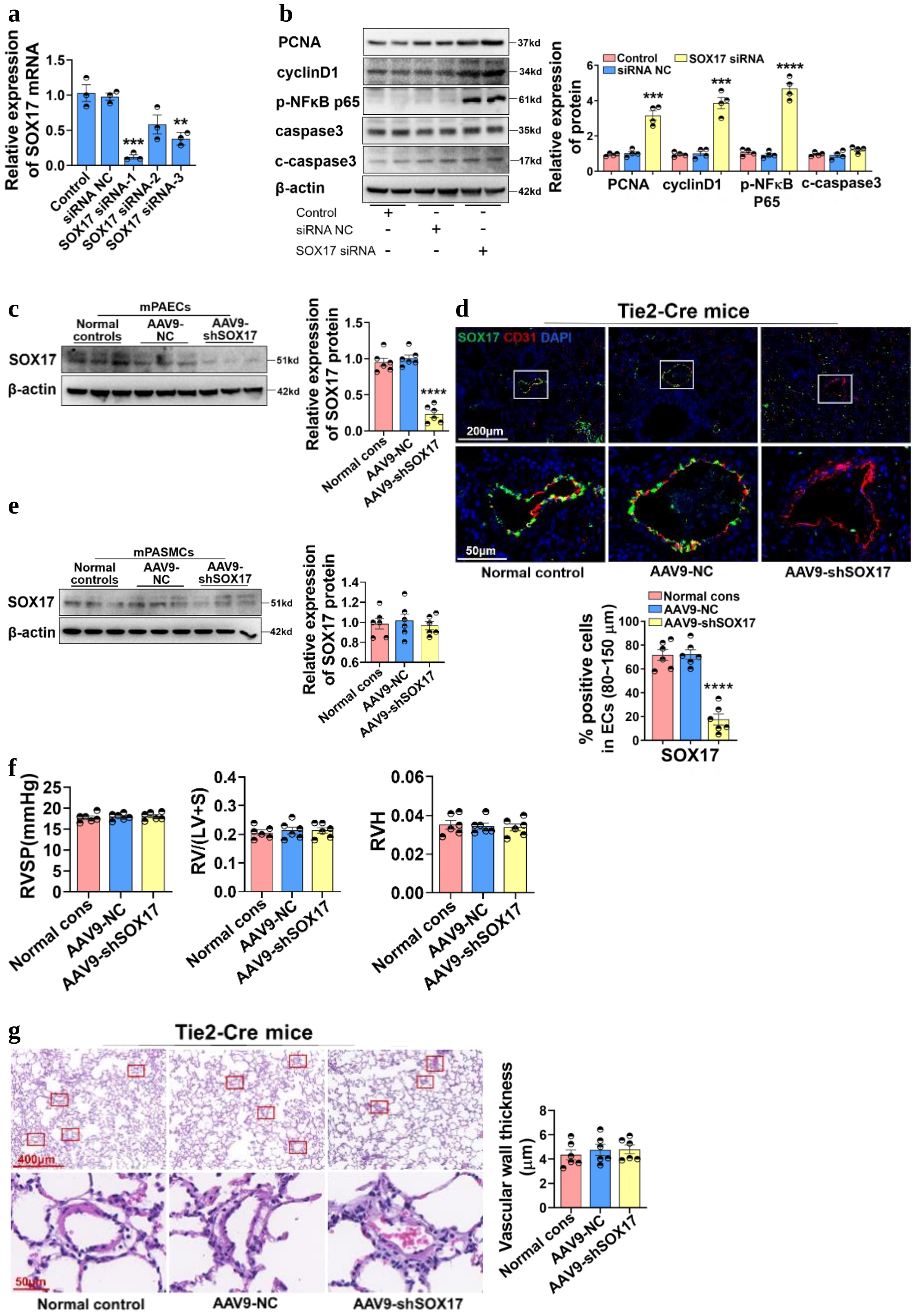

Figure S1 SOX17 knockdown in the pulmonary endothelium of Tie2-Cre mice did not cause PH. a Real-time PCR examination for the expression of SOX17 mRNA in the HPAECs treated with SOX17 siRNA, ***P* < 0.01, ****P* < 0.001 vs siRNA NC, n=3. **b** Western blotting examination for the expression of PCNA, cyclinD1, p-NFκB p65, caspase3m and cleaved-caspase3 proteins in the HPAECs treated with SOX17 siRNA, ****P* < 0. 01, *****P* < 0. 0001 vs siRNA NC, n=4. **c** Western blotting examination for the expression of SOX17 protein in the primary pulmonary artery endothelial cells separated from the Tie2-Cre mice treated with AAV9-shSOX17-loxp (AAV9-shSOX17), *****P* < 0.0001 vs AAV9-NC-loxp (AAV9-NC), n = 6. **d** Dual-IF staining for the location and expression of SOX17 and CD31 in the lung tissues of Tie2-Cre mice treated with AAV9-shSOX17, *****P* < 0.0001 vs AAV9-NC. **e** Western blotting examination for the expression of SOX17 protein in the primary pulmonary artery smooth muscle cells separated from the Tie2-Cre mice treated with AAV9-shSOX17-loxp (AAV9-shSOX17), n = 6. **f** RVSP, RV/LV+S, RVH in the Tie2-Cre mice treated with AAV9-shSOX17 were examined, n = 6. **g** HE staining for the pulmonary vascular remodeling in the Tie2-Cre mice treated with AAV9-shSOX17.

Figure S2

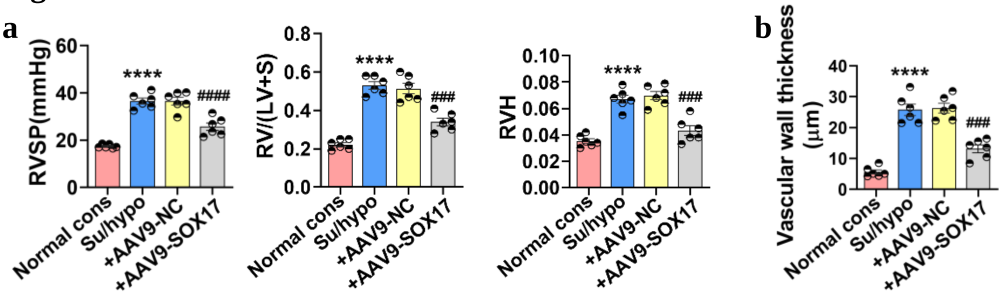

Figure S2 SOX17 overexpression in the pulmonary endothelium of Tie2-Cre mice attenuated Su/hypo-induced PH. a RVSP, RV/LV+S, RVH in the Tie2-Cre mice treated with Su/hypo and AAV9-SOX17 were examined, ****P < 0.0001 vs Normal controls, ###P < 0.001, ####P < 0.0001 vs Su/hypo+AAV9-NC, n = 6. **b** The quantification of vascular wall thickness (80-150 μm), ****P < 0.0001 vs Normal controls, ###P < 0.001 vs Su/hypo+AAV9-NC, n = 6.

Figure S3

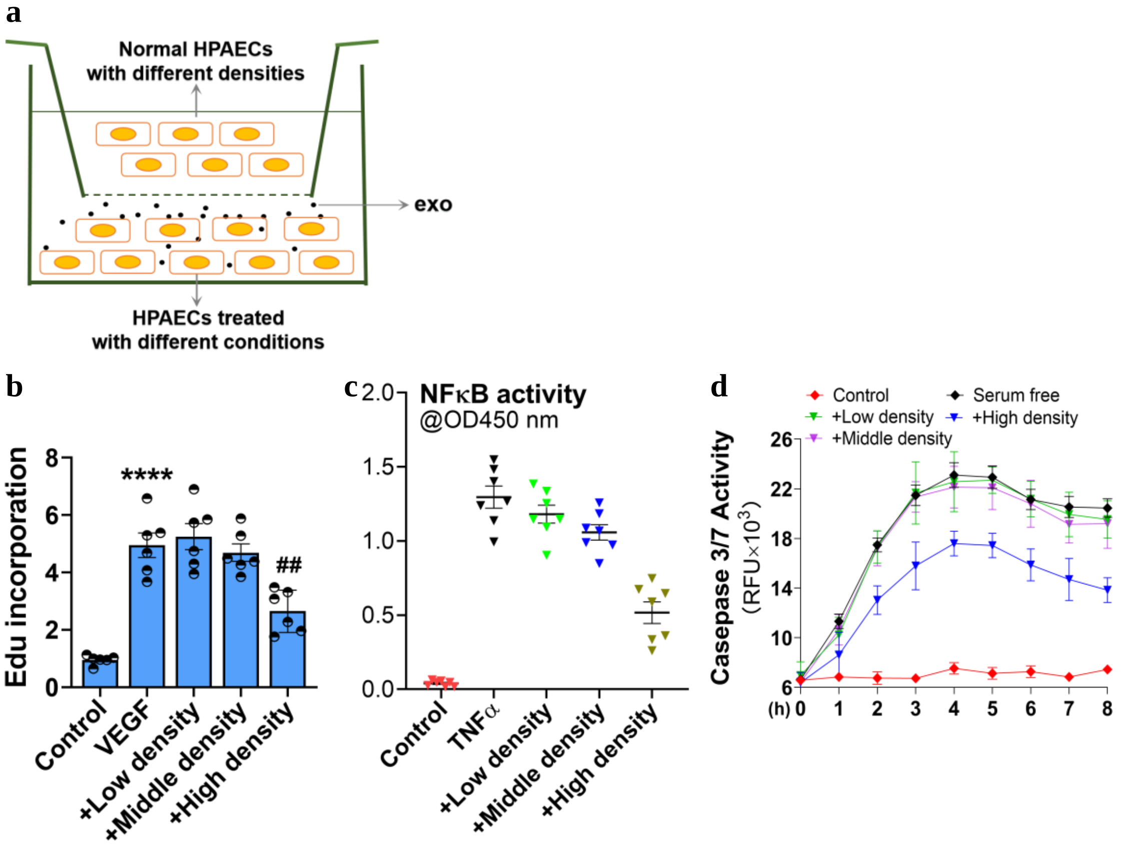

Figure S3 The endothelial dysfunction of HPAECs was alleviated under co-cultured condition.

(a) A porous membrane in a Transwell dish was used to separate HPAECs. The porous membrane is 0.4 μm and allows exchange of exosomal particles. HPAECs was seeded in a Transwell dish in 1×10^5 cells/mL and co-cultured with normal HPAECs in the low density of 1×10^4 cells/mL, middle density of 1×10^5 cells/mL, and high density of 1×10^6 cells/mL. **(b)** The DNA replication activity (Edu assay) for the proliferation induced by VEGF in HPAECs treated with HPAECs in different densities, $****P < 0.0001$ vs Control, $\#\#P < 0.01$ vs VEGF group, $n = 6$. **(c)** NFκB activation stimulated by TNF-α in HPAECs treated with HPAECs in different densities was examined by NFκB p65 transcription factor assay kit, $n = 7$. **(d)** Caspase 3/7 activation from 0~8 h induced by serum free in HPAECs treated with HPAECs at different densities was examined by a caspase 3/7 assay kits, $n = 6$.

Figure S4

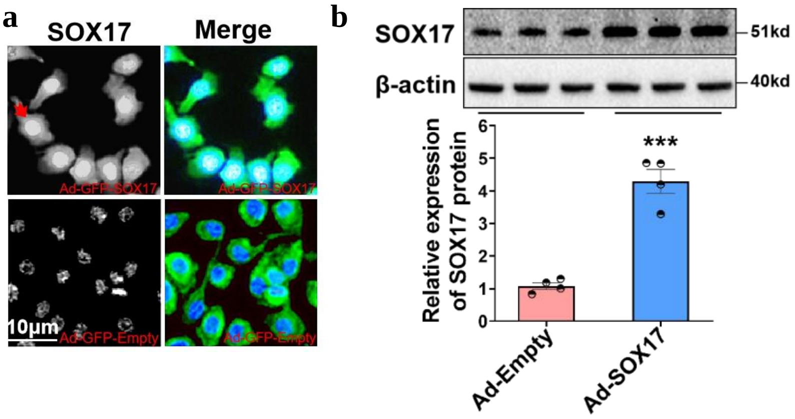

Figure S4 The expression of SOX17 was induced by Ad-SOX17 in HPAECs. (a) SOX17 expression was examined in HPAECs treated with Ad-GPF-SOX17, green fluorescence pointed infected cells and red arrowhead indicated location. **(b)** Western blotting assay for the expression of SOX17 in HPAECs treated by Ad-Empty or Ad-SOX17, n = 4, ****P* < 0.001 vs Ad-Empty.

Figure S5

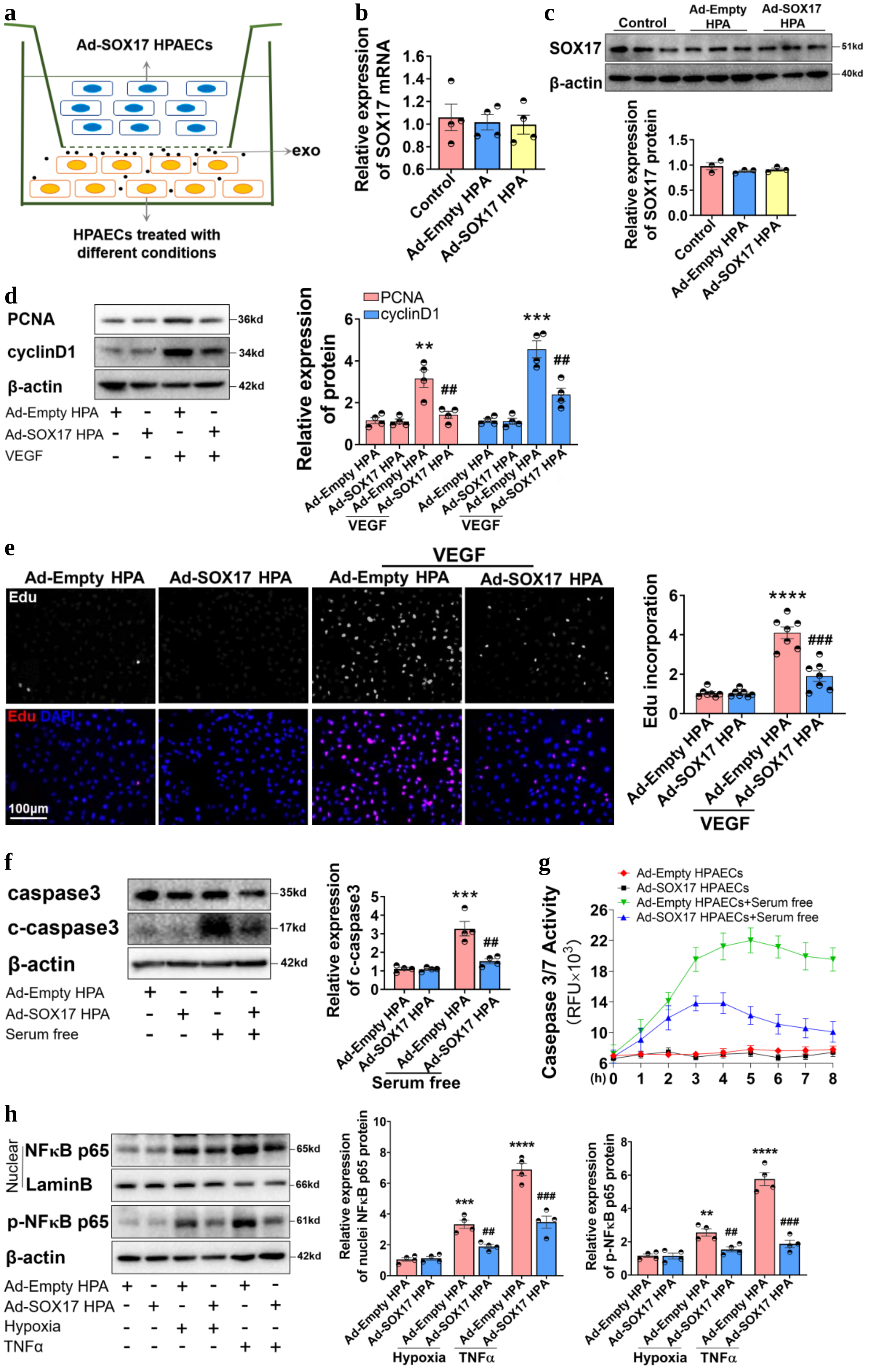

a
Ad-SOX17 HPAECs
exo
HPAECs treated with different conditions
b
Relative expression of SOX17 mRNA
Control
Ad-Empty HPA
Ad-SOX17 HPA
c
Control
Ad-Empty HPA
Ad-SOX17 HPA
SOX17
β-actin
51kd
40kd
Relative expression of SOX17 protein
d
PCNA
cyclinD1
β-actin
36kd
34kd
42kd
Ad-Empty HPA
Ad-SOX17 HPA
VEGF
Relative expression of protein
e
Edu
Edu DAPI
100μm
Edu incorporation
f
caspase3
c-caspase3
β-actin
35kd
17kd
42kd
Serum free
Relative expression of c-caspase3
g
Ad-Empty HPAECs
Ad-SOX17 HPAECs
Ad-Empty HPAECs+Serum free
Ad-SOX17 HPAECs+Serum free
Casepase 3/7 Activity (RFU×10³)
(h)
h
Nuclear
NFκB p65
LaminB
p-NFκB p65
β-actin
65kd
66kd
61kd
42kd
Hypoxia
TNFα
Relative expression of nuclei NFκB p65 protein
Relative expression of p-NFκB p65 protein

i

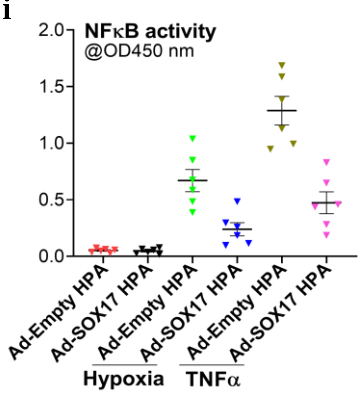

Figure S5 HPAECs dysfunction was reversed by SOX17-overexpressing HPAECs under co-cultured condition.

(a) A porous membrane in a Transwell dish was used to separate HPAECs from SOX1-overexpressing HPAECs. **(b)** qRT-PCR assay for the mRNA expression of SOX17 in HPAECs treated with SOX17-overexpressing HPAECs, n = 4. **(c)** Western blotting assay for the expression of SOX17 protein in HPAECs treated with SOX17-overexpressing HPAECs, n = 3. **(d)** Western blotting assay for the protein expression of PCNA and cyclinD1 induced by VEGF in HPAECs co-cultured with SOX17-overexpressing HPAECs, and the quantified data, ***P* < 0.01, ****P* < 0.001 vs Ad-Empty HPA group, ##*P* < 0.01 vs Ad-Empty HPA + VEGF group, n = 4. **(e)** The DNA replication activity (Edu assay) for the proliferation induced by VEGF in HPAECs treated with SOX17-overexpressing HPAECs, *****P* < 0.0001 vs Ad-Empty HPA, ###*P* < 0.001 vs Ad-Empty HPA + VEGF group, n = 6-7. **(f)** Western blotting assay for the protein expression of caspase3 and cleave-caspase3 induced by serum free in HPAECs treated with SOX17-overexpressing HPAECs, and the quantified data, ****P* < 0.001 vs Ad-Empty HPA; ##*P* < 0.01 vs Ad-Empty HPA + serum-free group, n = 4. **(g)** Caspase 3/7 activation from 0~8 h induced by serum free in HPAECs treated with SOX17-overexpressing HPAECs were examined by a caspase-3/7 assay kits, n = 6. **(h)** Western blotting assay for the protein expression of nuclear NFκB p65 and p-NFκB p65 stimulated by hypoxia and TNF-α in HPAECs treated with SOX17-overexpressing HPAECs, and the quantified data, ****P* < 0.001, *****P* < 0.0001 vs Ad-Empty HPA, ##*P* < 0.01, ###*P* < 0.001 vs Ad-Empty HPA + hypoxia or TNF-α, n = 4. **(i)** NFκB activation stimulated by hypoxia or TNF-α in HPAECs treated with SOX17-overexpressing HPAECs were examined by NFκB p65 transcription factor assay kit, n = 6.

Figure S6

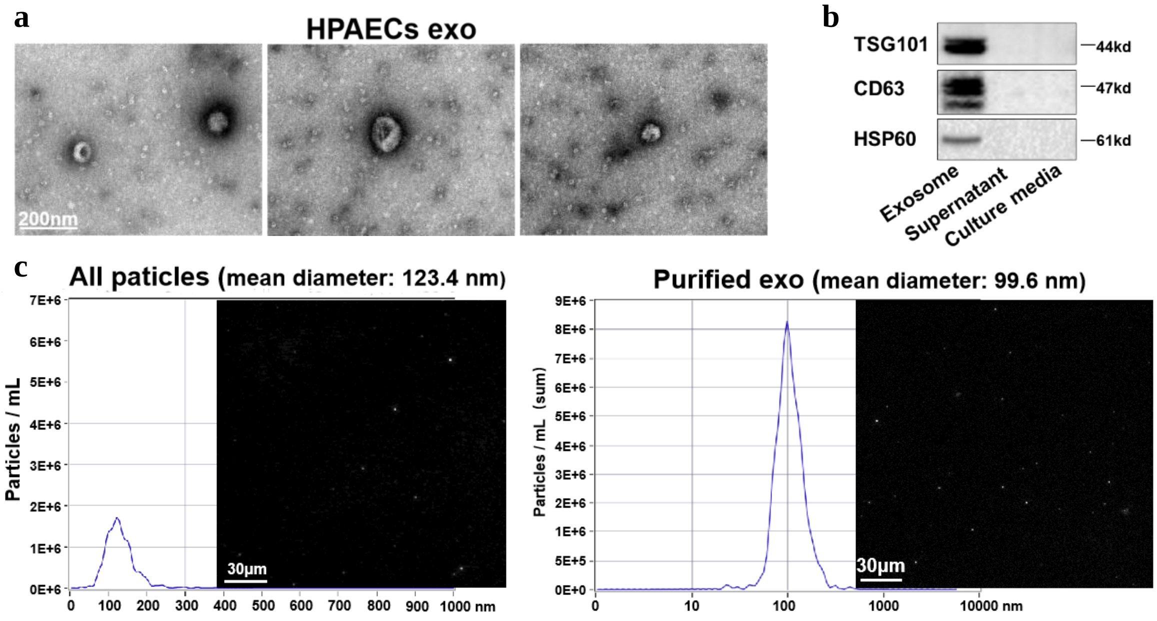

Figure S6 Exosomes generated from SOX17-overexpressing HPAECs was identified and confirmed.

(a-b) The transmission electron microscopy (TEM) examination for exosomes from SOX17-overexpressing HPAECs and western blotting examination for the exosomal markers including CD63, TSG101 and HSP60 in exosome pellet, supernatant, and culture media. **(c)** The particle size of total particles and exosomes in the purified exosome fraction of HPAECs detected by ZetaView PMX 110 Particle Size Analyzer and Particle Counter.

Figure S7

a

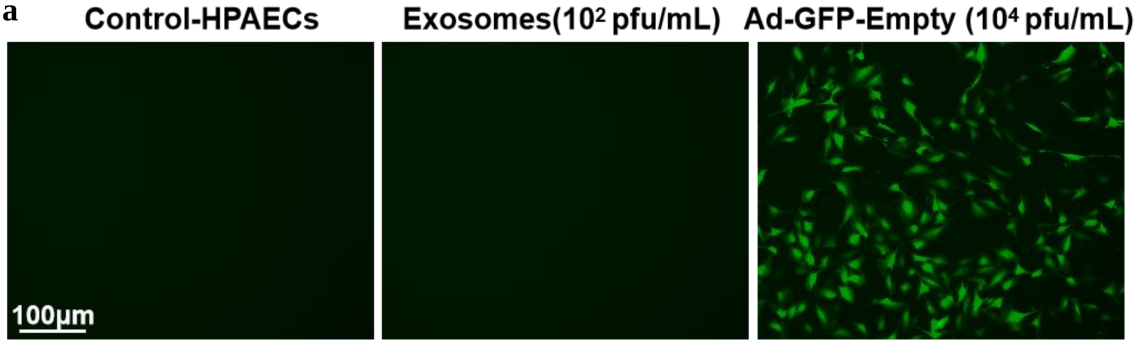

b

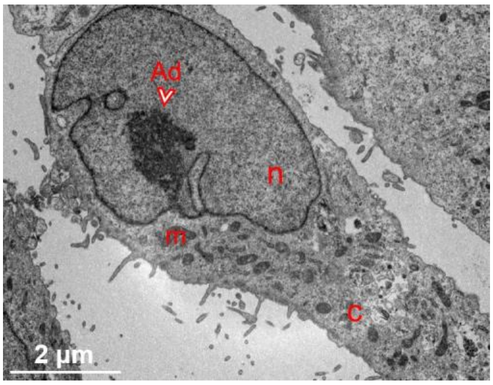

Figure S7 There was no adenovirus inside in exosome fractions.
(a) HPAECs were treated with SOX17-associated exosomes and Ad-GPF-Empty for 12 h, green represented infected cells. **(b)** HPAECs were treated with Ad-Empty for 12 h and detected by TEM. c-cytoplasm; m-mitochondrion, n-nucleus, Ad-adenoviral particles. Titration of the virus was carried out through preparing serial dilutions of the exosome fraction (10^{-2}-10^{-6}) in 96-well plates containing 10^2 cells per well, n=3-4.

Figure S8

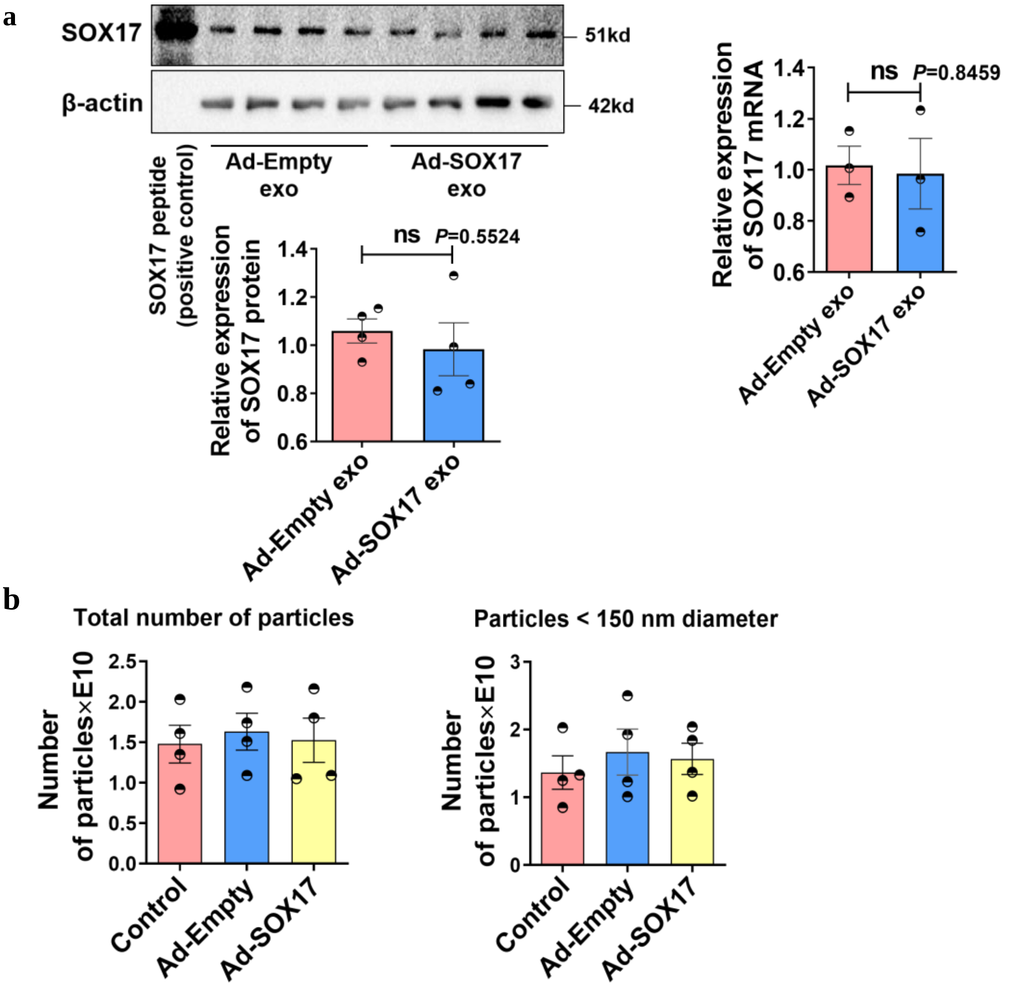

Figure S8 SOX17-associated exosomes had no influence on the expression of SOX17 in HPAECs and SOX17 overexpression had no influence on the number of exosomes generated from HPAECs.

(a) Western blotting and qRT-PCR assay for the expression of SOX17 in HPAECs treated with SOX17-associated exosomes, n = 3-4. **(b)** The number of total particles and exosomes (< 150 nm diameter) in HPAECs was detected by ZetaView PMX 110 Particle Size Analyzer and Particle Counter, n = 4.

Figure S9

a

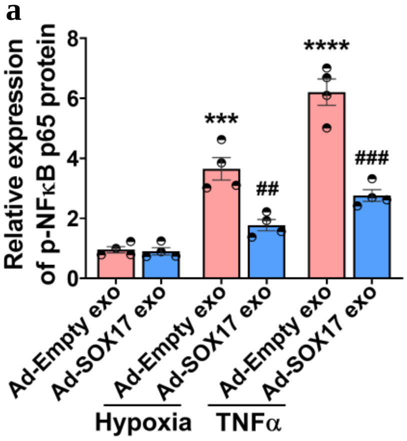

Figure S9 The quantification data of p-NFκB p65 protein.

(**a**) Quantification of western blotting assay for the protein expression of p-NFκB p65 stimulated by hypoxia and TNF-α in HPAECs treated with SOX17-associated exosomes, ***$P < 0.001$, ****$P < 0.0001$ vs Ad-Empty exo, ##$P < 0.01$, ###$P < 0.001$ vs Ad-Empty exo + hypoxia or TNF-α, n = 4.

Figure S10

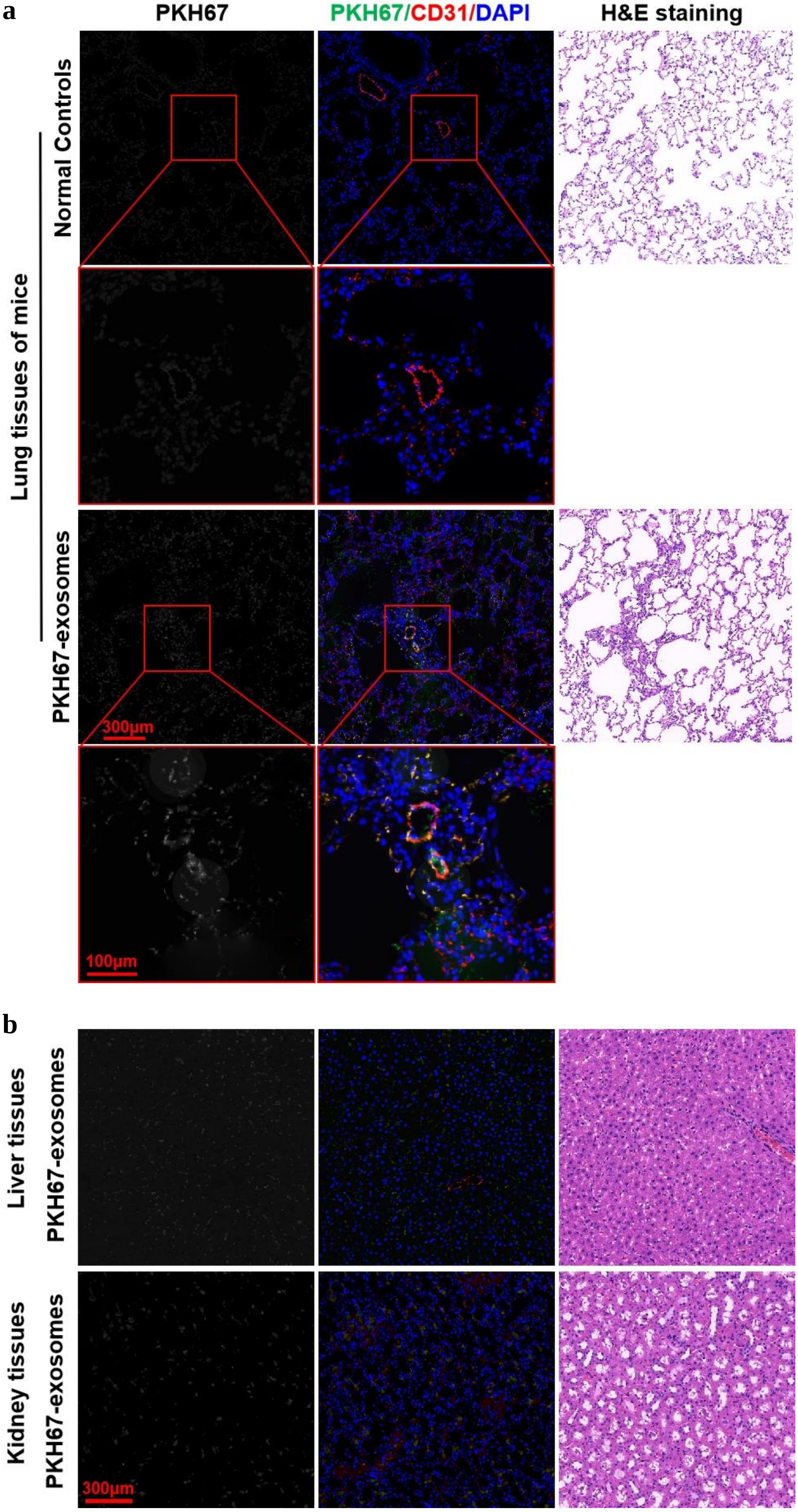
a
PKH67
PKH67/CD31/DAPI
H&E staining
Lung tissues of mice
Normal Controls
PKH67-exosomes
300μm
100μm
b
Liver tissues
PKH67-exosomes
Kidney tissues
PKH67-exosomes
300μm

Figure S10 Visualization of PKH67-labelled exosomes *in vivo*.

SOX17-associated exosomes were labelled with PKH67 and injected into mice through tail veins (about 3×10^{11} particles every 100 µL PBS). Multiple-IF and HE staining were conducted to detect the localization of exosomes in **(a)** lung, **(b)** liver, and kidney tissues. PKH67 was labelled with delight 488 and indicated exosomes, and CD31 was labeled with Cy3 and indicated vascular endothelium.

Figure S11

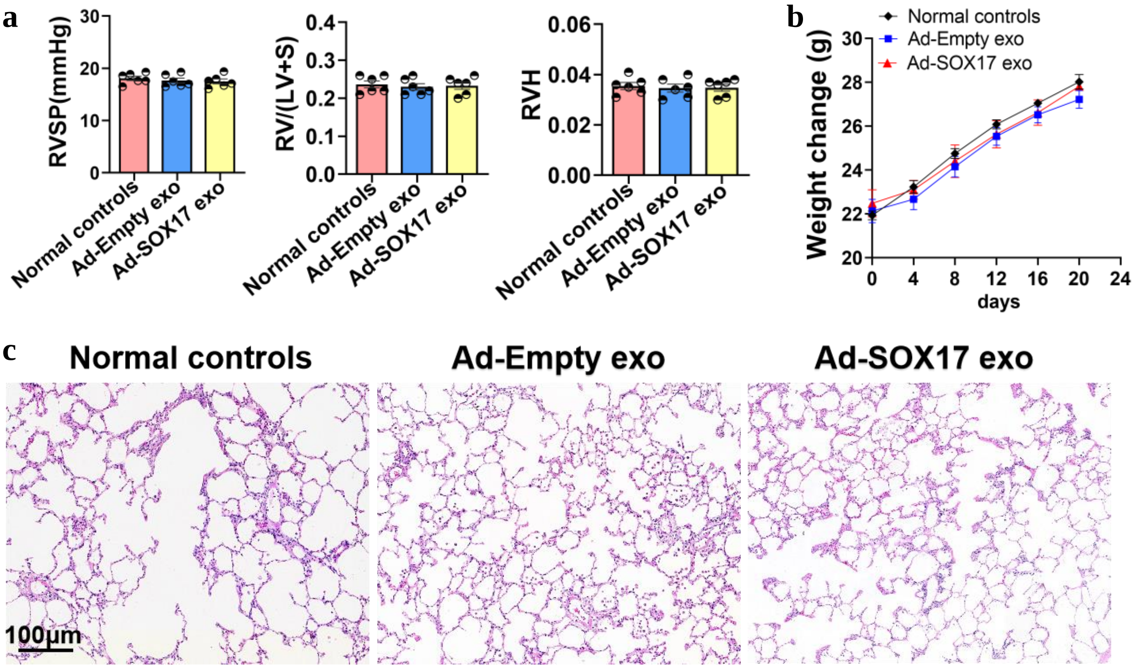

Figure S11 SOX17-associated exosomes injection had no influence in the hemodynamic index and the morphology of lung tissues from normal mice.

(a-b) RVSP, RV/LV+S, RVH, and body weight in the mice treated with SOX17-associated exosomes were examined, n = 6. **(c)** HE staining assay for the lung tissues of mice treated with SOX17-associated exosomes.

Figure S12

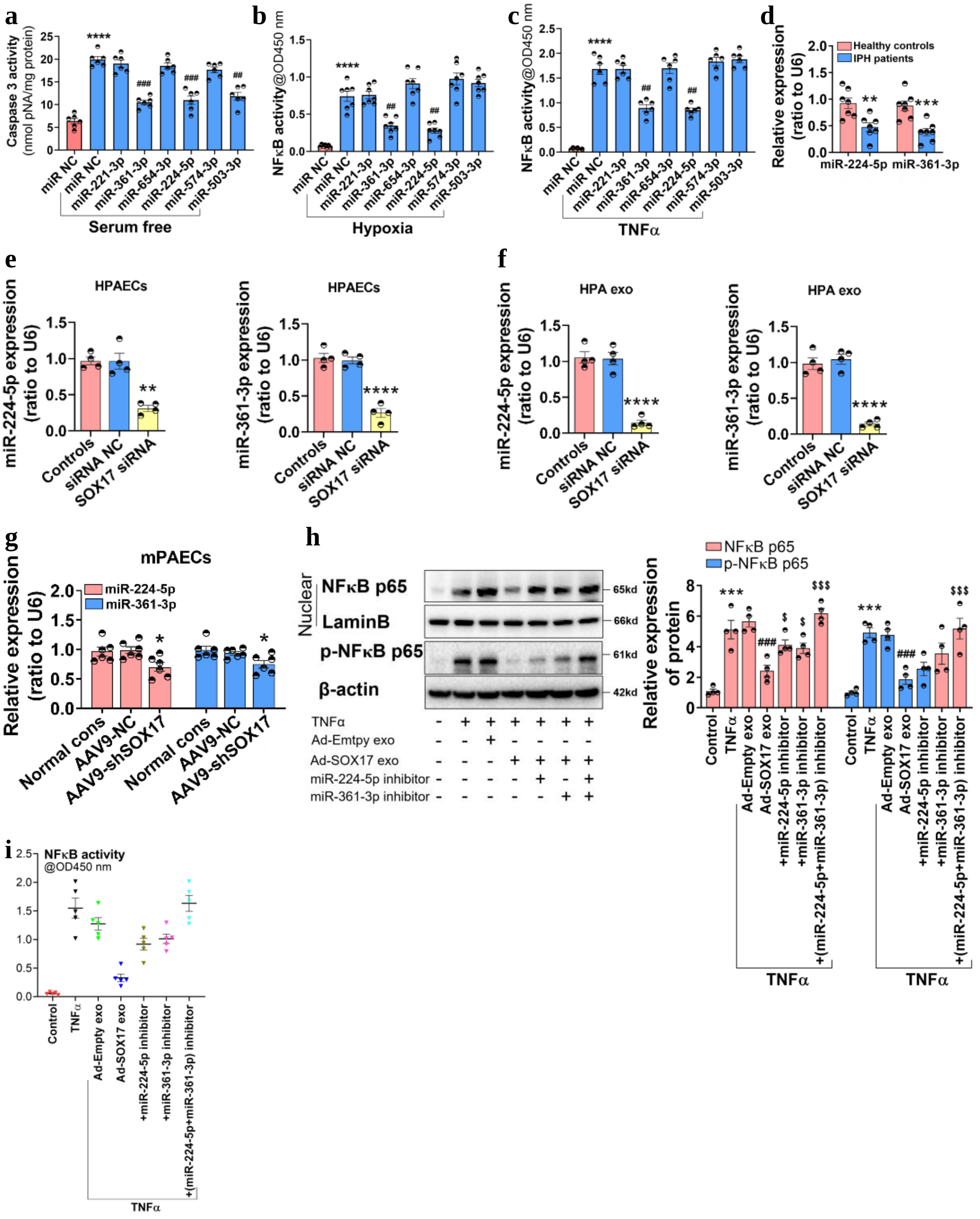
a
b
c
d
Caspase 3 activity (nmol pNA/mg protein)
Serum free
NFκB activity@OD450 nm
Hypoxia
TNFα
Healthy controls
IPH patients
Relative expression (ratio to U6)
miR-224-5p
miR-361-3p
e
f
HPAECs
HPA exo
miR-224-5p expression (ratio to U6)
miR-361-3p expression (ratio to U6)
Controls
siRNA NC
SOX17 siRNA
g
mPAECs
Normal cons
AAV9-NC
AAV9-shSOX17
h
Nuclear
NFκB p65
LaminB
p-NFκB p65
β-actin
65kd
66kd
61kd
42kd
TNFα
Ad-Emtpy exo
Ad-SOX17 exo
miR-224-5p inhibitor
miR-361-3p inhibitor
Relative expreision of protein
Control
Ad-Empty exo
+miR-224-5p inhibitor
+miR-361-3p inhibitor
+(miR-224-5p+miR-361-3p) inhibitor
i
NFκB activity
@OD450 nm

Figure S12 miR-224-5p and miR-361-3p were selected and mediated the protective role of SOX17-associated exosomes in the inflammation response.

(a) Caspase 3 activity induced by serum free in HPAECs transfected with miRNA mimics were examined by caspase-3 assay kits, ****P < 0.0001 vs miR NC, ##P < 0.01, ###P < 0.001 vs miR NC + serum free, n = 6. **(b-c)** NFκB activity induced by hypoxia or TNFα in HPAECs transfected with miRNA mimics was examined by NFκB p65 transcription factor assay kits, n = 6-7, ****P < 0.0001 vs miR NC, ##P < 0.01 vs miR NC + Hypoxia or TNFα. (**d**) Real-time PCR assay for the expression of miR-224-5p and miR-361-3p in the lung tissue homogenates from healthy controls and PH patients (n = 7), **P < 0.01, ***P < 0.001 vs healthy controls. (**e**)Real-time-PCR assay for the expression of miR-224-5p and miR-361-3p in HPAECs treated with SOX17 siRNA, **P < 0. 01, ****P < 0. 0001 vs siRNA NC, n=4. (**f**) Real-time-PCR assay for the expression of miR-224-5p and miR-361-3p in the exosomes generated from HPAECs treated with SOX17 siRNA, ****P < 0. 0001 vs siRNA NC, n=4. (**g**) Real-time PCR assay for the expression of miR-224-5p and miR-361-3p in the primary pulmonary artery endothelial cells from Tie2-Cre mice treated with AAV9-shSOX17 (mPAECs), *P < 0.05 vs AAV9-NC, n=6. (**h**) Western blotting assay for the protein expression of nuclear NFκB p65 and p-NFκB p65 induced by TNFα in HPAECs treated with SOX17-associated exosomes and miR inhibitors, ***P < 0.001 vs Control, ###P < 0.001 vs TNFα + Ad-Empty exo, \$$P$ < 0.05, \$\$\$$P$ < 0.001 vs TNFα + Ad-SOX17 exo, n = 4. (**i**) NFκB p65 transcription factor assay kits for the NFκB activity in HPAECs, n = 5.

Figure S13

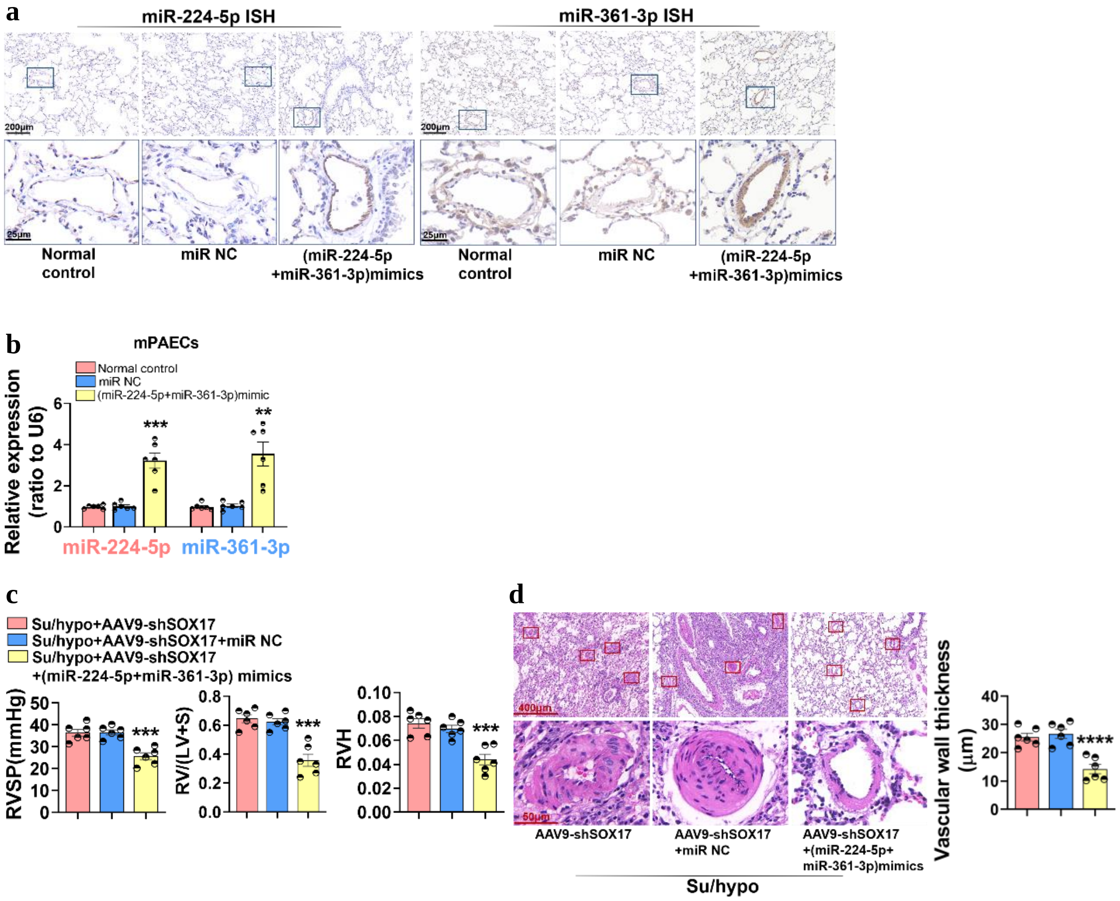

Figure S13 Combined treatment of miR-224 and miR-361 mimics reversed PH and vascular remodeling in the Tie2-Cre mice induced by Su/hypo and SOX17 knockdown. (a-b) A-B RNA-ISH assay for the expression of miRNA-224-5p and miR-361-3p in the lung tissues of mice treated by miRNA-224-5p and miR-361-3p mimics through tail intravenous injection, $^{**}P < 0.01$, $^{***}P < 0.001$ vs miR NC, n = 6. **(c)** RVSP, RV/LV+S, RVH in the Tie2-Cre mice treated with Su/hypo, AAV9-shSOX17 and miR mimics were examined, $^{***}P < 0.001$ vs Su/hypo+AAV9-shSOX17+miR NC group, n = 6. **(d)** HE staining for the vascular remodeling in the Tie2-Cre mice, and the quantification of vascular wall thickness (80-150 μm), $^{****}P < 0.0001$ vs Su/hypo+AAV9-shSOX17+miR NC group, n = 6.

Figure S14

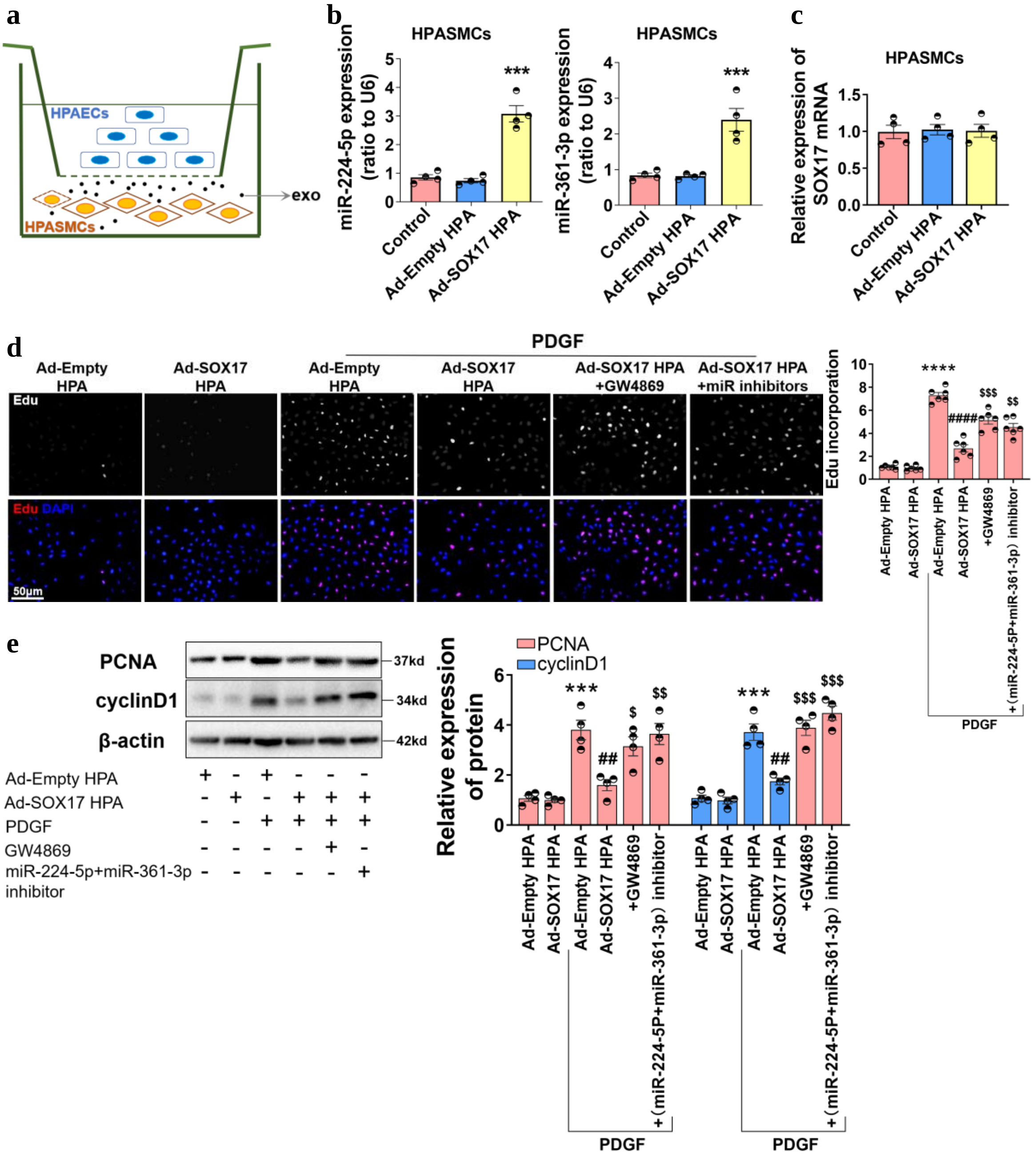

Figure S14 SOX17-overexpressing HPAECs blocked the proliferation of HPASMCs through exosomes.

(a) A porous membrane in a Transwell dish was used to separate HPASMCs from SOX1-overexpressing HPAECs. **(b)** qRT-PCR assay for the expression of miR-224-5p and miR-361-3p in HPASMCs co-cultured with SOX17-overexpressing HPAECs, n = 4, ****P* < 0.001 vs Ad-Empty HPA group. **(c)** qRT-PCR assay for the mRNA expression of SOX17 in HPASMCs co-cultured with SOX17-overexpressing HPAECs, n = 4. **(d)** The DNA replication activity (Edu assay) for the proliferation induced by PDGF in HPASMCs co-cultured with SOX17-overexpressing HPAECs and treated with exosomal inhibitor (GW4869) or miR-224-5p and miR-361-3p inhibitors. GW4869 was served as

a negative control, ****$P < 0.0001$ vs Ad-Empty HPA, ####$P < 0.0001$ vs Ad-Empty HPA + PDGF, $$$P < 0.01$, $$$ $P < 0.001$ vs Ad-SOX17 HPA + PDGF, n = 6. **(e)** Western blotting assay for the expression of PCNA and cyclinD1 proteins induced by PDGF in HPASMCs co-cultured with SOX17-overexpressing HPAECs and treated with exosomal inhibitor (GW4869) or miR-224-5p and miR-361-3p inhibitors, and the quantified data, ***$P < 0.001$ vs Ad-Empty HPA, ##$P < 0.001$ vs Ad-Empty HPA + PDGF, $$P < 0.05$, $$$P < 0.01$, $$$ $P < 0.001$ vs Ad-SOX17 HPA + PDGF, n = 4.

Figure S15

a

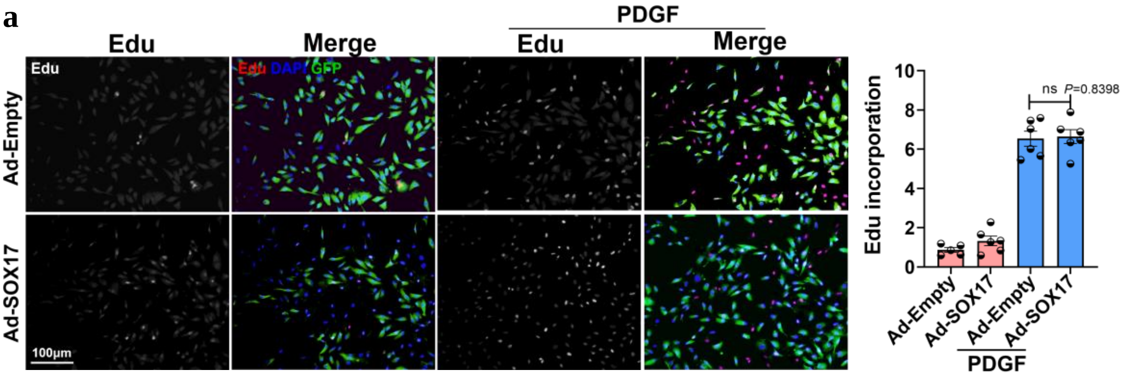

Figure S15 SOX17 overexpression in HPASMCs had no influence on the proliferation of HPASMCs. (a) Representative images and corresponding graph showed the effect of Ad-GFP-SOX17 on the proliferation of HPASMCs treated with PDGF, n = 5-6. Blue staining represented nuclei (DAPI), red staining represented nuclei incorporating Edu, and green staining represented cells infected with Ad-GFP-Empty or Ad-GFP-SOX17.

Figure S16

a

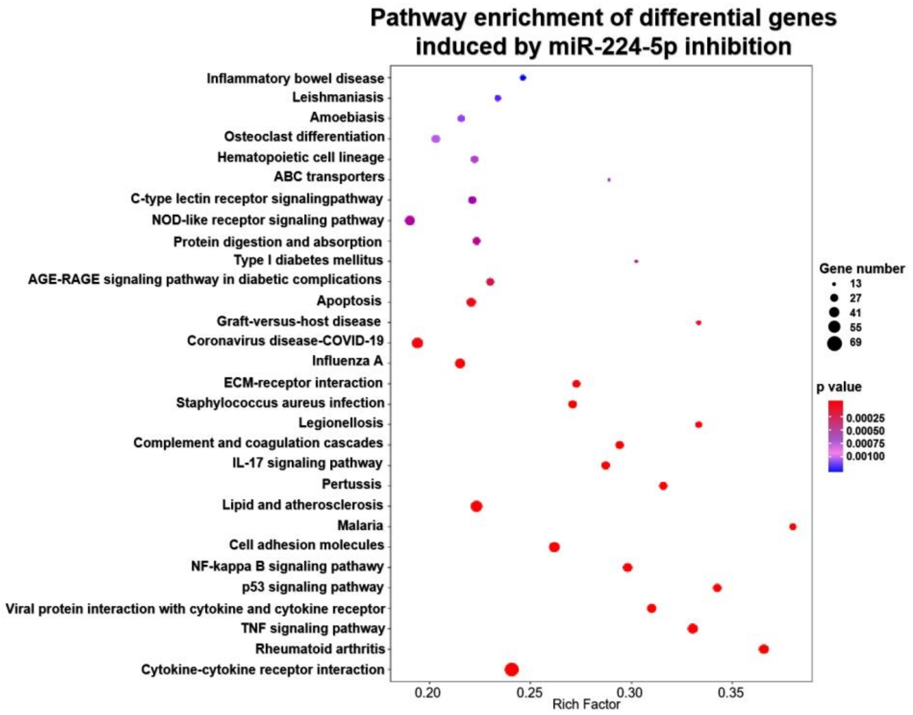

b

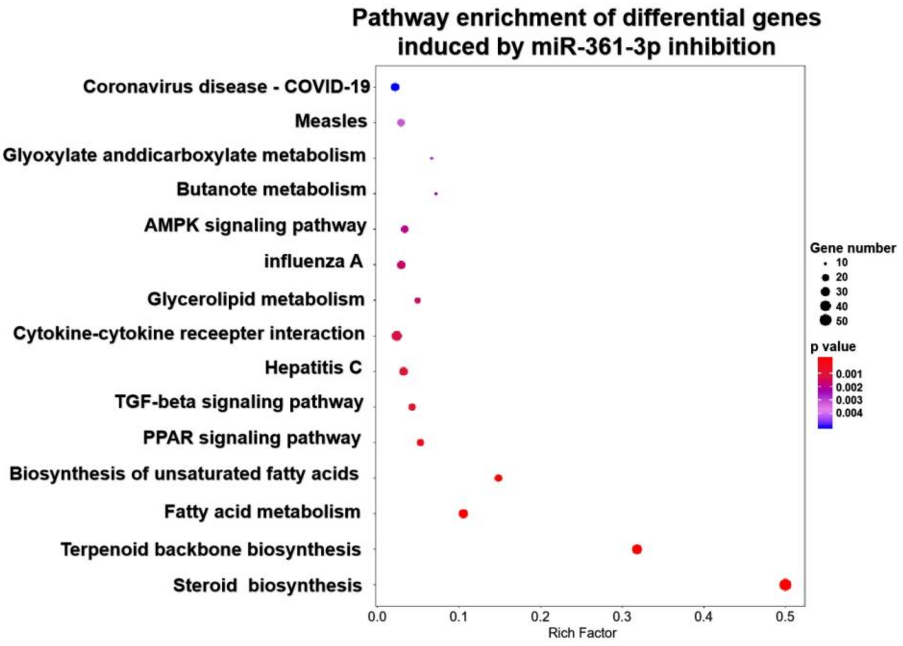

Figure S16 The analysis of differential transcripts induced by miR-224-5p and miR-361-3p inhibition.

(a-b) Pathway enrichment (KEGG database) analysis in HPAECs transfected with miR-224-5p or miR-361-3p inhibitor.

Figure S17

a

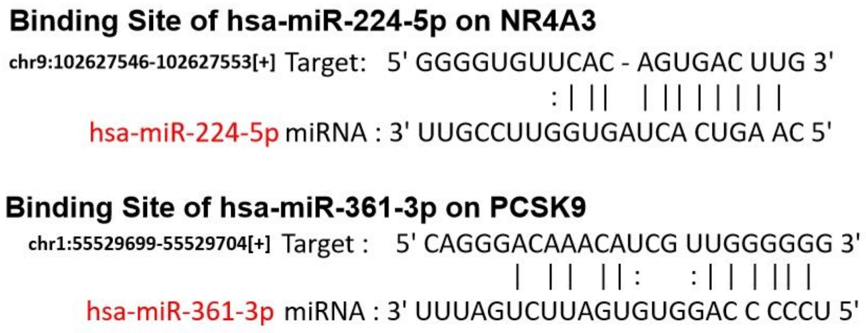

Figure S17 The binding sites of miR-224-5p in NR4A3 and miR-361-3p in PCSK9 (using miRmap and PITA database).

Figure S18

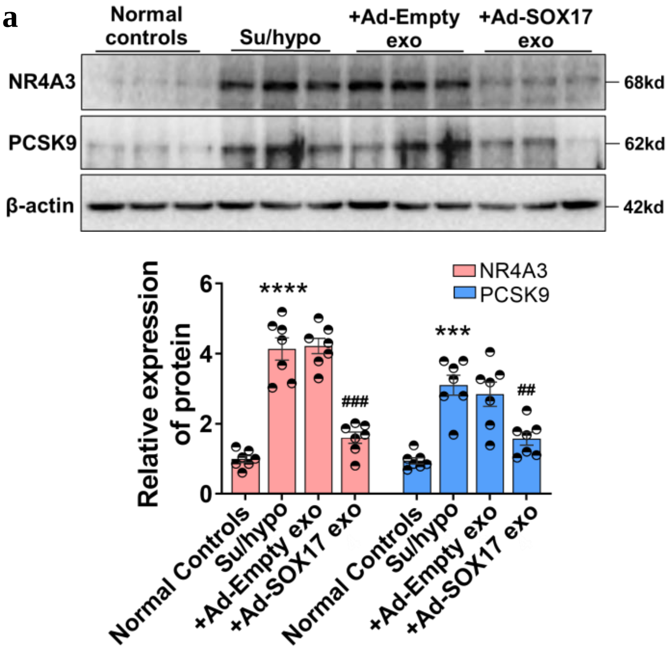

Figure S18 SOX17-associated exosomes blocked the up-regulation of NR4A3 and PCSK9 induced by Su/hypo in the lung tissues of PH mice

(a) Western blotting assay for the expression of NR4A3 and PCSK9 proteins in the lung tissues of healthy mice, PH mice and PH mice treated with SOX17-associated exosomes, ***$P < 0.001$, ****$P < 0.0001$ vs Normal Controls, ##$P < 0.01$, ###$P < 0.001$ vs Su/hypo + Ad-Empty exo group, n = 7.

Figure S19

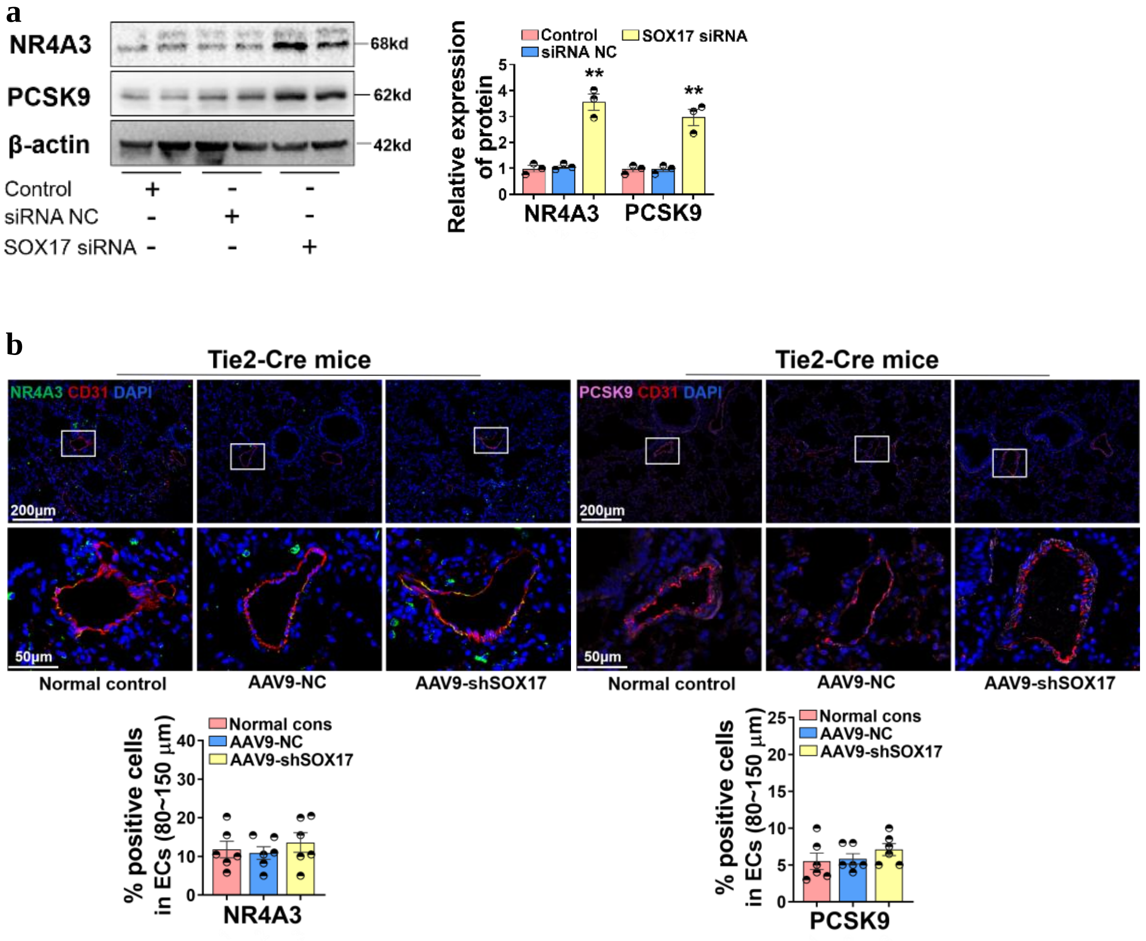

Figure S19 The regulation of SOX17 knockdown in the expression of NR4A3 and PCSK9 *in vitro* and *in vivo*. (a) Western blotting examination for the expression of NR4A3 and PCSK9 in HPAECs treated with SOX17 siRNA, ** $P < 0.01$ vs siRNA NC, n=3. **(b)** Dual-IF staining for the location and expression of NR4A3 or PCSK9 and CD31 in the lung tissues of Tie2-Cre mice treated with AAV9-shSOX17, n = 6.

Figure S20

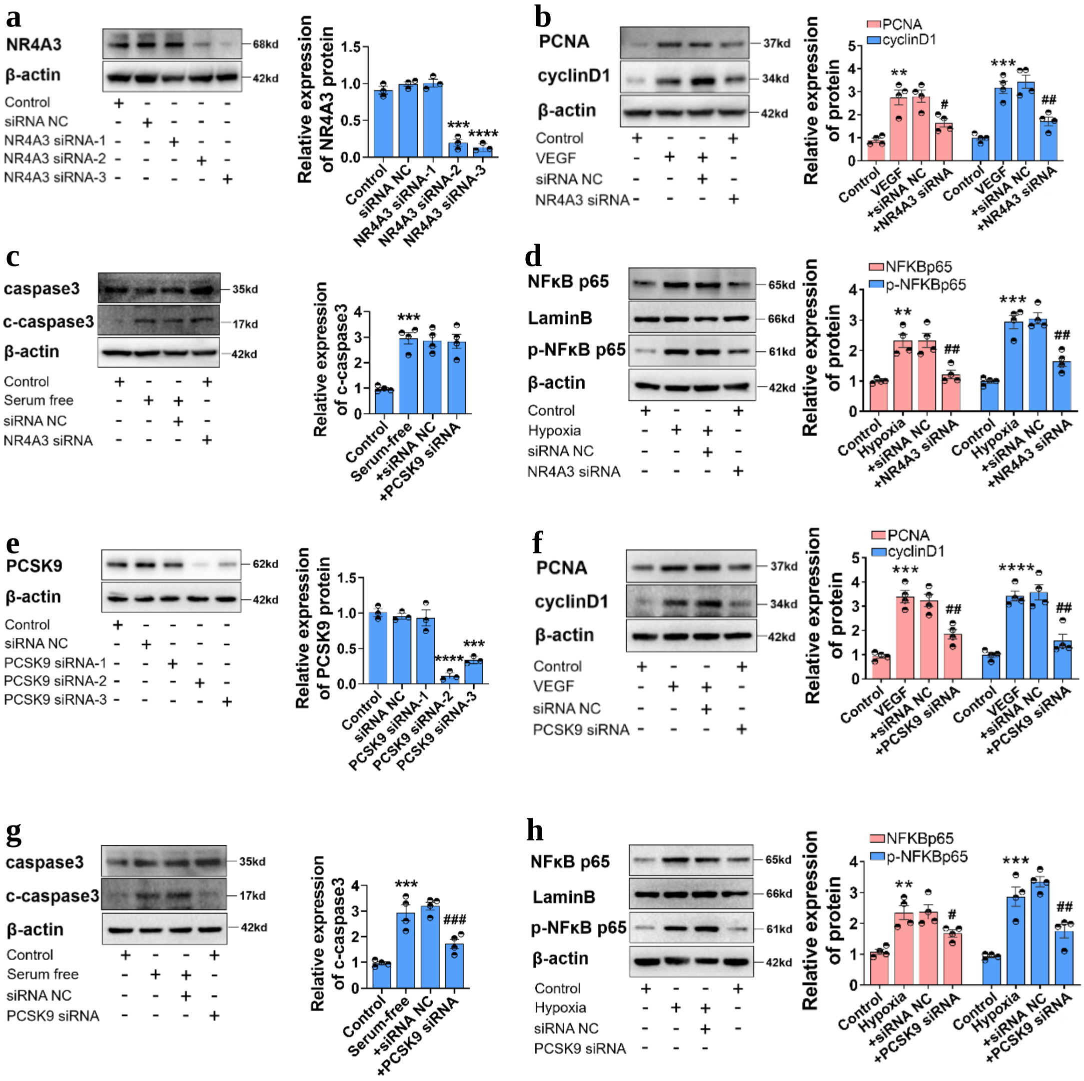

Figure S20 NR4A3 and PCSK knockdown blocked the VEGF-induced proliferation, serum free-induced apoptosis, and hypoxia-induced inflammation of HPAECs. (a) Western blotting examination for the protein expression of NR4A3 in HPAECs treated with NR4A3 siRNA, ***P < 0.001, ****P < 0.0001 vs siRNA NC, n = 3. **(b-d)** Western blotting examination for the protein expression of PCNA, cyclinD1, c-caspase3/caspase3, NFκB p65，p-NFκB p65 in HPAECs treated with NR4A3 siRNA and VEGF or Serum free or Hypoxia, **P < 0.01, ***P < 0.001 vs Control, #P < 0.05, ##P < 0.01 vs treatment + siRNA NC, n = 4. **(e)** Western blotting examination for the protein expression of PCSK9 in HPAECs treated with PCSK9 siRNA, ***P < 0.001, ****P < 0.0001 vs siRNA NC, n = 3. **(f-h)** Western blotting examination for the protein expression of PCNA, cyclinD1, c-caspase3/caspase3, NFκB p65, p-NFκB p65 in HPAECs treated with PCSK9 siRNA and VEGF or serum free or hypoxia, **P < 0.01, ***P < 0.001, ****P < 0.0001 vs Control, #P < 0.05, ##P < 0.01, ###P < 0.001 vs treatment + siRNA NC, n = 4.

Figure S21

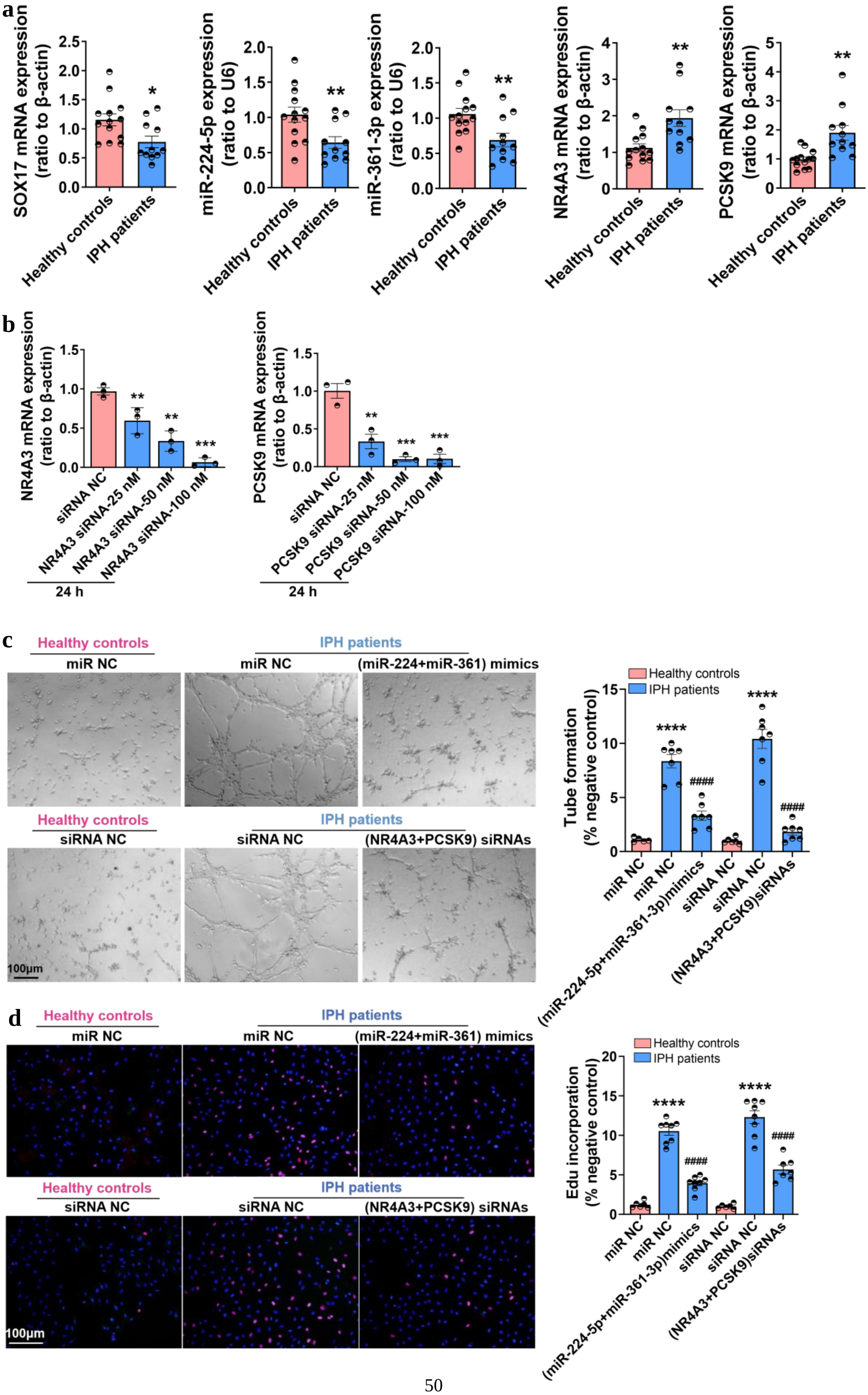
a
SOX17 mRNA expression (ratio to β-actin)
miR-224-5p expression (ratio to U6)
miR-361-3p expression (ratio to U6)
NR4A3 mRNA expression (ratio to β-actin)
PCSK9 mRNA expression (ratio to β-actin)
Healthy controls
IPH patients
b
NR4A3 mRNA expression (ratio to β-actin)
siRNA NC
NR4A3 siRNA-25 nM
NR4A3 siRNA-50 nM
NR4A3 siRNA-100 nM
24 h
PCSK9 mRNA expression (ratio to β-actin)
PCSK9 siRNA-25 nM
PCSK9 siRNA-50 nM
PCSK9 siRNA-100 nM
c
Healthy controls
IPH patients
miR NC
(miR-224+miR-361) mimics
siRNA NC
(NR4A3+PCSK9) siRNAs
100μm
Tube formation (% negative control)
(miR-224-5p+miR-361-3p)mimics
(NR4A3+PCSK9)siRNAs
d
Edu incorporation (% negative control)

Figure S21 SOX17 signaling was activated in ECFCs from IPH patients and inhibited the injury of ECFCs.

(a) qRT-PCR assay for the mRNA expression of SOX17, miR-224-5p, miR-361-3p, NR4A3 and PCSK9 in ECFCs from healthy controls (n =13-14) and PH patients (n = 11-12), $*P < 0.05$, $**P < 0.01$ vs healthy controls. **(b)** HPAECs were treated with NR4A3 siRNA and PCSK9 siRNA for 24h, and the mRNA expression of NR4A3 and PCSK9 were examined by qRT-PCR, $**P < 0.01$, $***P < 0.001$ vs siRNA NC, n = 3. **(c-d)** The tube formation and proliferation were examined in ECFCs treated with miR-224-5p and miR-361-3p mimic or NR4A3 and PCSK9 siRNA, values are mean fold-changes of miR NC or siRNA NC. Tube formation of live ECFCs were labelled with fluorescent and shown underneath the graph, $****P < 0.0001$ vs miR NC or siRNA NC of health controls, $\#\#\#\#P < 0.0001$ vs miR NC or siRNA NC of IPH patients, n = 5-8.

Supplementary Methods

Cell cultures

Cells were cultured in standard incubator conditions including 5% CO_2, 21% O_2, and 37℃. Cells were treated using serum free, recombinant human TNF-α (PeproTech, 300-01A, 50 ng/mL, 24 h), VEGF165 (PeproTech, GMP100-20, 100 ng/mL, 18 h), hypoxia (1% O_2, 48 h) and PDGF BB (Stem RD, HY-000170, 10 ng/mL, 48h). Alternatively, the cells were seeded into 6-, 12-, 24-, 96-well culture plates (Corning, China).

HPAEC and HPASMC cultures

HPAECs were kindly provided by Prof. Hu from the Xiangya School of Pharmaceutical Sciences at Central South University in China. Before culturing, T-25 flasks (Corning, China) were coated with a solution containing bovine plasma fibronectin (Sciencell^{TM}, cat.8248) diluted using phosphate-buffered saline (PBS) to 20 mg/L. The cells were cultured in ECM endothelial cells medium (Sciencell^{TM}, cat.1001), supplemented with 5% fetal bovine serum (FBS) (Sciencell^{TM}, cat.0025), 1% endothelial cell growth supplement (ECGS, cat.1052), and 1% antibiotic solution (P/S, cat. 0503).

HPASMCs from Sciencell^{TM} were maintained in T-25 flasks in SMCM smooth muscle cell medium (Sciencell^{TM}, cat.1101), supplemented with 2% FBS, 1% smooth muscle cell growth supplement and 1% antibiotic solution (P/S, cat.0503). Cells between passages 4 and 10 were used to conduct experiments. Cells that were positive for α-SMA antibody staining were selected for further analysis.

Primary mPAEC and mPASMC separation and cultures

The primary pulmonary artery endothelial cells (PAECs) were separated from Tie2-Cre mice and cultured. After anesthesia, pulmonary arteries were first isolated from the left lobe of lung tissues owing to the relative ease of working with a single, large lobe. The intrapulmonary arteries of the first to third order branches were selected and carefully dissected away from connective tissues under a light microscope. After removing the adventitia, the isolated pulmonary arteries were digested in the endothelial basal medium (EBM; Lonza, PromoCell) containing type V collagenase (175U.ml-1) and neutral protease (0.8U.ml-1) at 37°C for 1 hour, shaking every 5 min. Digestion was terminated with the endothelial growth medium (EGM; Lonza, PromoCell) containing 5% fetal

bovine serum (FBS Gibco™, NY, United States) and filtered through a 200-mesh sieve to remove incompletely digested tissue. The filtered digest was then centrifuged at 1000rpm for 5 min and the cells were resuspended and incubated with anti-mouse CD31-FITC (Biolegend, cat.102413) endothelial cell labeling antibody at room temperature and protected from light for 15 min. Then CD31 microbeads (Miltenyi Biotec, Order No. 130-097-418) were added and incubated at room temperature for 15 min away from light. After centrifugation at 2000rpm for 10 min, the cells were re-suspended with 500ul EBM, and the re-suspended cell suspension was added to the MS column (Miltenyi Biotec, Order No. 130-042-201) to flow freely. 3ml EBM cleaning MS column 3 times; 5 ml EGM with 5% FBS was added and the cells were quickly pushed out using a piston. The expelled cell suspension is placed in an incubator for culturing.

The primary PASMCs were isolated from Tie2-Cre mice and cultured as previously described[1]. In brief, pulmonary arteries were first isolated from the left lobe of lung tissues owing to the relative ease of working with a single, large lobe. The intrapulmonary arteries of the first to third order branches were selected and carefully dissected away from connective tissues under a light microscope. After removing the adventitia, the isolated pulmonary arteries were digested in dispersion medium containing 40 μmol/l $CaCl_2$, 1 mg/ml elastase, 1 mg/ml collagenase, 0.2 mg/ml soybean trypsin inhibitor, and 2 mg/ml albumin for 30 min at 37 °C. After filtration with 100-μm cell strainers, PASMCs were collected through centrifugation at 225 × g for 10 min and subcultured with Dulbecco's Modified Eagle's medium (DMEM) containing 10% fetal bovine serum (FBS) and antibiotics. Only early-passage (passage 3-5) PASMCs were used for all experiments.

Endothelial colony-forming cell (ECFC) cultures

Human endothelial cells, also known as ECFCs or late outgrowth endothelial progenitor cells, were derived from the peripheral blood[2]. All investigations were conducted as per the Declaration of Helsinki. The ethics approval for studies on human subjects was provided by the Ethics Committee of Zhong Nan Hospital of Wuhan University (2021077k) and informed written consent was obtained from all the donors. Table S5

shows the clinical features and demographics of the doners. The venous blood samples were placed in vacutainers containing EDTA, and were diluted 1:1 with PBS containing 2% FBS and 0.2% EDTA. Next, the samples were layered onto Ficoll Plaque^{TM} Plus (Amersham Biosciences, USA) for density gradient centrifugation. Peripheral blood mononuclear cells (MNCs) were washed with PBS and resuspended in EGM-2 medium (Lonza, CC-3156) containing 20% FBS, growth factors (Lonza, CC-4176, EGM™-2 bullet kit), and 1% antibiotic and antimycotic solution (ScienCell^{TM}, cat.0503). MNCs were seeded into 6-well plates coated with rat tail tendon collagen type I (NC-Bio, LCB002) at a density of ~ 4×10^7 MNCs in 5 mL medium per well. To exclude non-adherent cells, the culture medium was replaced every day for the first week, and in the next few weeks the medium was replaced every 3 days. Individual colonies were harvested by using cloning equipment (Sigma-Aldrich, C7983-50EA) with vacuum grease and using preheated 0.05% Trypsin-EDTA solution (Sigma-Aldrich, T4049) to remove any cells in the equipment. The collected cells were resuspended in fresh EGM-2 medium, seeded in culture vessels precoated with 1% gelatin at a density of 4×10^7 cells/cm^2, and propagated until 80%-90% confluent. Distinct colonies of cells, capable of serial propagation for at least eight serial passages, clonal growth, and displaying a stable population doubling time and endothelial phenotype were used between passages 4 to 7. The endothelial cell lineage was confirmed using specific markers, including anti-CD31 antibody (Abcam, ab24590), anti-Von Willebrand Factor (vWF) antibody (Abcam, ab195028), and FITC-conjugated Ulex europaeus agglutinin-1 (UEA-1) lectin. As previously described, a three-color FACSCalibur flow cytometer (BD Biosciences) was used to analyze stained cells via flow cytometry[2].

Acquisition of lung tissues

Lung samples of patients with IPH

Surgical samples of lung tissues were obtained from patients with IPH undergoing transplantation (n = 7), and healthy lung tissue samples were acquired from donors that had suffered accidents or sudden illnesses (n = 7). All investigations were conducted in accordance with the principles outlined in the Declaration of Helsinki. Ethical approval for studies on human subjects was provided by the Ethics Committee of Zhong Nan

Hospital of Wuhan University (2021077k). Informed consent was obtained prior to acquisition of samples from all donors. Clinical information for the patients is shown in Table S6. Paraffin sections were used for histopathological examination. Fresh lung tissues stored in liquid nitrogen were transported by dry ice and stored at -80 °C for further analysis. Tissue samples (excluding major arteries and airways) were cut from the lung's periphery for western blotting analysis. Lung samples, 200 mg each, were washed with 1 mL ice-cold PBS containing 1 mM EDTA and 1 mM L-dithiothreitol and were homogenized for 2 min using a PT-K Polytron® Stand Homogenizer (Kinematica AG, Switzerland). The protein content was measured using BCA protein quantitation kits (Beyotime, P0010).

Lung samples from mice

All the experimental procedures were performed and approved by the Animal Ethics Committee of Zhejiang Provincial People's Hospital (A20220069).

C57BL/6 mice (20 ~ 22 g, male, 8 weeks old) were sourced from the SLAC Laboratory Animal Co. (Shanghai, China) and randomly assigned to the various experimental groups. Age and weight-matched mice were selected for analysis. Su/hypo groups received a single weekly subcutaneous injection of SU5416 (20 mg/kg) and were exposed to hypoxia (10% O_2, 3 weeks) as previously described[3]. Control groups were injected with vehicle alone. Ad-SOX17 exosome groups were treated using Su/hypo and injected with SOX17-associated exosomes labeled with PKH67 (~3 × 10^{11} particles per 100 µL PBS) thrice a week. Ad-Empty exosome groups were treated using Su/hypo and received an equal number of exosomes from control samples.

Tie2-Cre mice were purchased from the GemPharmatech Co. Ltd. (Strain number: T003764). AAV9-shSOX17-loxp (AAV9-shSOX17), and its negative control AAV9-NC-loxp (AAV9-NC), AAV9-SOX17-loxp (AAV9-SOX17) and its negative control AAV9-NC-loxp (AAV9-NC) were constructed by Genechem Co. Ltd. The Tie2-Cre mice (20 ~ 25 g, male, 6 ~ 8 weeks old) were given AAV9-shSOX17 (1E+11 v.g/mouse) and AAV9-NC (1E+11v.g/mouse) through a single tail vein injection and feed for 21 days to build endothelial-specific SOX17 knockdown mice. The efficiency of knockdown of SOX17 was examined by the protein expression of SOX17 using western blotting and dual-IF

staining for the location and expression of SOX17 and CD31 in the lung tissues of mice. For constructing the endothelial-specific SOX17 overexpression mice, the Tie2-Cre mice were given AAV9-SOX17 (1E+11v.g/mouse) and AAV9-NC (1E+11v.g/mouse) through a single tail vein injection and feed for 21 days. Control groups were injected with vehicle alone. mirVana® miRNA mimic of miR-224-5p (Ambion, AssayID MC12336), miR-361-3p (Ambion, AssayID MC19234), and negative control #1 (Ambion) were mixed with transfection reagent (Invitrogen, Invivofectamine® 3.0). miRNA-overexpressing Tie2-Cre mice groups were injected through tail vein injection once every three days. Scramble groups were injected with the miR negative control alone. The Tie2-Cre mice with endothelial-specific SOX17 knockdown or overexpression as well as miRNA overexpression were further treated by Su/hypo to induce PH.

As described previously[4], mice were anesthetized via intraperitoneal injection with pentobarbitone (30 mg/kg) and ventilated through a transtracheal catheter. A left parasternal incision was made, a 1.2-F (for mice) micro-tip pressure transducer catheter (Millar Instruments, USA) was carefully inserted immediately into the right ventricular apex, and right ventricular systolic pressure (RVSP) was recorded continuously for 2 min using the PowerLab Data Acquisition System (ADInstruments, Australia). After hemodynamic measurements, the lung and heart tissues were obtained and weighed. The left lungs of the animals were flash-frozen in liquid nitrogen for subsequent homogenization and RNA/protein extraction. The lower lobes of the right lungs were fixed in 4% paraformaldehyde. Morphometric analysis was performed by an investigator blinded to the experimental procedure. Pulmonary arterioles with external diameters <100 μm in the entire lung tissue were selected. Right ventricular hypertrophy was determined from the ratio of the weight of the right ventricle to the weight of the left ventricle plus septum (RV/LV+S). The tibia was dissected for calculating RV weight/tibial length. All analyses were performed in a blinded manner.

Cell transfection

Ad-Empty or Ad-SOX17 were constructed by Shanghai GeneChem Company Limited (China). Ad-Empty or Ad-SOX17 was infected into HPAECs (multiplicity of infection

was 1:100) or HPASMCs (multiplicity of infection was 1:250), respectively, when cells reached 70%-80% confluency. The culture medium was replaced after 4 h, and a subset of cells were treated with Serum free, VEGF165, hypoxia, TNF-α, and PDGF.

*mir*Vana® miRNA mimic of miR-224-5p (Ambion, AssayID MC35019), miR-361-3p (Ambion, AssayID MC11176), miR-221-3p (Ambion, AssayID MC10337), miR-654-3p (Ambion, AssayID PM11205), miR-574-3p (Ambion, AssayID MC12848), and miR-503-3p (Ambion, AssayID MC25720), Anti-miR™ miRNA inhibitor of miR-224-5p (Ambion, AssayID MH35019) and miR-361-3p (AssayID MH11176), and negative control #1 (Ambion) were transfected into HPAECs using the Lipofectamine™ RNAiMAX Transfection Reagent (Invitrogen™, cat.13778075) according to the manufacturer's instructions. NR4A3 and PCSK9 siRNA and negative control were purchased from Santa Cruz (sc-38842, sc-45482) and transfected using the Lipofectamine™ RNAiMAX Transfection Reagent. After cells were cultured in ECM medium supplemented with 1% FBS, 1% endothelial cell growth supplement (ECGS, cat.1052), and 1% antibiotic solution (P/S, cat.0503) for 12 h, the medium was replaced with fresh medium containing miRNA mimics, inhibitors, or negative controls (a transfection reagent control). After 12 h, the medium was replaced with fresh complete medium, and the cells were administered hypoxia treatment, TNF-α, or were kept serum-free. The working concentration of miR-224-5p, miR-361-5p, miR-221-3p, miR-654-3p, miR-574-3p, and miR-503-3p mimics used was 200 nM, and the concentration of miR-224-5p and miR-361-3p inhibitors used was 100 nM and 75 nM, respectively. Cy3 dye-labeled pre-miR negative control (20 nM; Ambion, AM17120) was used to assess the transfection efficiency. NR4A3 siRNAs (50 nM), PCSK9 siRNA (50 nM), or negative control siRNA (50 nM) were transfected into HPAECs using Lipofectamine 3000 (Invitrogen, USA) according to the manufacturer’s instructions before hypoxia or TNF-α or serum-free treatment.

Exosome purification and quantification

HPAECs were cultured in ECM medium supplemented with 2% exosome-depleted serum (Gibco, A2720801, USA) and treated with Ad-Empty or Ad-SOX17. The supernatant from the cells was used to separate exosomes using the Umibio® exosome

isolation kit (Umibio, UR52121, China) according to the manufacturer's instructions. In brief, an initial spin was performed at 3000 ×*g* at 4℃ for 10 min and 10,000 ×*g* at 4℃ for 20 min to remove the cells and debris, and the corresponding reagents were added proportionally to the starting sample volume. Mixtures were vortexed and incubated at 4℃ for 1.5-2 h and centrifuged at 10,000 ×*g* at 4℃ for 1 h to precipitate exosome pellets. Pellets were resuspended in PBS and purified using an exosome purification filter at 3000×*g* at 4℃ for 10 min. According to the manufacturer's instructions, the resuspension volume for exosome pellets was 200 µL for a starting volume of 20 mL. The purified exosomes were stored at -80℃ immediately after isolation until further use. The ZetaView PMX 110 Particle Size Analyzer and Particle Counter (Particle Metrix Ltd) was used to track and analyze the size distribution and concentration of exosomes in liquid suspension. The presence of exosome membrane markers was confirmed by western blotting analysis. BCA protein quantitation kits (Beyotime, P0010) were used to measure protein concentrations for the exosome fractions. Eight µg protein from exosome samples were added into each well of 10% SDS/PAGE gels and subjected to electrophoresis. PVDF membranes (Immobilon, IPVH00010) were incubated with exosome biomarkers including anti-TSG101 (Abcam, ab125011, 1:2000), anti-CD63 (Abcam, ab134045, 1:1000) and anti-HSP60 antibodies (Abcam, ab190828, 1:1000). Secondary antibodies used were HRP-linked goat anti-rabbit IgG (Beyotime, A0208, 1:2000). BeyoECL Plus (Beyotime, P0018M) was used to visualize the target bands using a ChemiDoc™ Imager (Bio-Rad, USA). Image J software was used to detect the relative intensity of the immunoreactive bands.

The purified exosome fractions were diluted 50-fold to incubate with HPAECs. After 24 h, the expression of SOX17 in HPAECs was analyzed. The membranes were incubated with anti-SOX17 (Abcam, ab224637, 1:1000) and anti-β-actin antibodies (Proteintech, 20536-1-AP, 1:2000). The secondary antibodies used were HRP-linked goat anti-rabbit IgG (Beyotime, A0208, 1:2000). SOX17 peptide (Abcam, 253177) was used as a positive control.

Analysis of exosome internalization

To visualize exosomes *in vitro*, 20 mL of conditioned medium was used to isolate

exosomes and the total number of purified exosomes used was ~3.5×10^{12} particles. About 5×10^{5} exosomes per cell were used to incubate HPAECs. The exosomal cell membrane was labeled with PKH26 red fluorescent cell membrane linker (Sigma-Aldrich, PKH26GL) which was added into the cells to detect internalization. According to the manufacturer's protocol, the exosomes were washed by PBS after labeling with PKH26 and centrifuged at $1 \times 10^{5} \times g$ for 15 min at 4 °C to clear the unbound stain. Next, the PKH26-labeled exosomes were added to HPAECs seeded on coverslips and incubated in 6-well dishes for 2 h at 37 °C. The cells were fixed in 4% formaldehyde for 15 min and permeabilized with 0.1% Triton X-100 for 10 min. The cells were next incubated with phalloidin FITC reagent (Abcam, ab235137) to examine F-actin expression, and the coverslips were washed by PBS and stained with DAPI. After elution, the Olympus IX71 fluorescence microscope (Tokyo, Japan) was used to detect and capture photos.

To detect exosomes *in vivo*, PKH26-labeled exosomes (~3×10^{11} particles in 100 μL of PBS) were injected into healthy mice through the tail vein. Eight h later, the distribution of exosomes in each tissue was analyzed. Lungs, livers, and kidneys were obtained to prepare paraffin sections. Immunofluorescence (IF) and Hematoxylin and Eosin (HE) staining were performed to visualize the protein expression or histopathological change.

Quantitative real-time polymerase chain reaction (qRT-PCR)

RNA isolation and qRT-PCR were performed as described previously[5]. Total RNA was extracted using Trizol reagent (Invitrogen, cat.15506-026) from lung tissues and cells. cDNA was synthesized using the PrimeScript reverse transcription reagent kit. qRT-PCR was performed in an ABI 7300 real-time PCR system using the SYBR Green PCR Master Mix kit. β-actin was used as an internal control to normalize the amount of sample messenger RNA (mRNA). The primers used in the present study are listed in Table S7. The relative expression of RT-PCR products was analyzed via the comparative threshold cycles (Ct) method using the 7300 System SDS software.

EdU proliferation assay

HPAEC EdU proliferation assay after co-cultured with SOX17-overexpressing HPAECs

According to the manufacturer's protocol, EdU Cell Proliferation Assay Kit (EMD Millipore Corp, 17-10527) was used to detect the proliferation. In total, SOX17-overexpressing HPAECs (at 1×10^4, 1×10^5, and 1×10^6 cellular density) were seeded into the Transwell inserts, whereas normal HPAECs (at 1×10^5 cellular density) was seeded onto plastic coverslips (Thermo Fisher Scientific, 174950) inserted into 24-well plate. Then, Transwell inserts with SOX17-overexpressing HPAECs were placed in the wells containing normal HPAECs and the two cell types were co-cultured for further 18 h under VEGF165 (PeproTech, GMP100-20) treatment, supplemented with 10 µM EdU. At the end of the incubation period, the medium was removed, and the cells were fixed in 3.7% formaldehyde solution (Fisher Scientific, F/1501/PB17) in PBS for 15 min at room temperature. After aspiration of the fixing solution, the cells were washed twice with 3% BSA (Sigma-Aldrich, A3294) in PBS (Scientific Laboratory Supplies, LZ51226) and permeabilised with 0.5% Triton X-100 (Sigma-Aldrich, 9002-93-1) for 20 min at room temperature. The cells were then washed twice again with 3% BSA. Two hundred microliters of EdU reaction cocktail prepared according to the manufacturer's instructions were added to the wells. After 30 min incubation in the dark at room temperature, reaction cocktail was removed, and cells were washed three times with 3% BSA.

To visualise cell nuclei, 5 mM stock Nuclear Green DSC1 (Abcam, ab138905) was diluted to 1:1000 in PBS and 200 µl of this solution were added to each well. The cells were incubated for 1 h in the dark at room temperature and then washed two times with PBS. The coverslips were then removed and mounted on a glass slide (VWR, 6310107) in Vectashield® Antifade Mounting Medium (Vector Laboratories, H-1000). The cells were examined under a fluorescent microscope (Olympus IX70). EdU-incorporating nuclei were visualised at Ex/Em 590/617 nm, while all nuclei stained with Nuclear Green were visualised at Ex/Em 503/526 nm. Cell nuclei were counted using ImageJ software with a macro designed by Steven Rothery (Facility for Imaging by Light Microscopy, Department of Medicine, Imperial College London, UK). Data are shown as fold-change in the percentage of EdU-incorporating cells in VEGF-treated cells, compared with untreated controls.

HPAEC EdU proliferation assay after SOX17-associated exosomes treatment

To study the effect of endothelial SOX17-associated exosomes on VEGF-induced HPAECs proliferation, HPAECs were seeded in a T-25 flask at 60% confluency and transfected the day after with Ad-Empty or Ad-SOX17, as previously described. After transfection, 5 mL of growth factor- and heparin-free, serum-reduced (2% exosome-depleted FBS) ECM were added to each flask. After 24 h, endothelial cell media were collected, centrifuged for 10 min at 1×10^4 g to remove cell debris, and stored at -20 °C. Then, normal HPAECs were seeded onto coverslips inserted into the wells of the 24-well plate (at 1×10^5 cells cellular density) and cultured in normal ECM for 24 h. The medium was removed, and the cells were starved for 2 h in serum- and growth factor-free ECM. Conditioned media from Ad-Empty and Ad-SOX17-overexpressing HPAECs were defrosted, supplemented with 10 µM EdU, with or without VEGF165 and added to normal HPAECs. EdU proliferation assay was performed, as previously described.

HPASMC EdU proliferation assay under co-cultured with HPAECs

In total, HPAECs (at 1×10^5 cellular density) were seeded into the Transwell inserts, whereas HPASMCs (at 3×10^5 cellular density) were seeded onto plastic coverslips (Thermo Fisher Scientific, 174950) inserted into 24-well plate. HPAECs were infected with Ad-Empty or Ad-SOX17 and 4 h later the cells were transfected with miRNA inhibitors or negative controls. Next day, Transwell inserts with HPAECs were placed in the wells containing HPASMCs and the two cell types were co-cultured for further 48 h with or without 10 ng/mL PDGF BB. In addition, to verify the role of exosomes in the observed cell responses, the co-cultured cells treated by PDGF BB were also incubated with exosomal inhibitor GW4869. Twenty-four hours before the end of the experiment, 10 µmol/L EdU was added to the cells. EdU proliferation assay was performed, as previously described.

HPAEC and ECFC EdU proliferation assay

HPAECs were cultivated in 96-well black polystyrene microplates with clear bottoms (Corning, CLS3603) at a density of 15,000 cells per well (untreated, treated with exosomes, untransfected or transfected with control miRNA, miRNA mimic, or siRNA, as appropriate). For 8 h, the cells were pre-starved in growth factor-depleted ECM media

containing 1% FBS. In total, VEGF165 and 10 μM EdU were added 18 h before the end of the experiment. ECFCs from healthy donors and IPH patients were seeded at a density of 15,000 cells per well in 96-well black polystyrene microplates with clear bottoms and grown for 8 h in growth factor-free EGM-2 media (Lonza, CC-3156) with 5% FBS and 1% antibiotic and antimycotic solution added (ScienCellTM, 0503). The cells were cultured for another 18 h after treatment with 10 μM EdU. EdU proliferation assay was performed, as previously described.

Apoptosis assay

Apo-One Homogenous Caspase-3/7 assay kit (Promega, G7790) was used to detect cellular apoptosis. According to the instruction of the manufacturer, cells were treated as per the experimental conditions with relevant negative controls included in the assay. Each well of the black polystyrene microplates (Corning, CLS3603) was incubated with 300 μL of cell suspension, and the cell concentrations were controlled at 2-8×10^6 cells/mL. After serum free treatment, the cells were incubated with Apo-One^{R} Assay kits to examine the activity of Caspase-3/7 from 0h to 8h. The Glomax^{TM} luminometer was used to measure the fluorescence intensity of the red fluorescent Rhodamine 110. The plate reader was set to perform an endpoint read. Rhodamine 110 has an optimal excitation and emission wavelength of 498nm to 521 nm. The quantity of fluorescent product represents the activity of caspase 3/7 in the sample.

Tube formation assay

The process was conducted on ice. Before experiments were conducted, Matrigel (Corning, 356234/5 milliliters), tips, and 48-well plates were placed at 4°C for 24 h. The icebox was placed under an ultraviolet lamp for sterilization. Melted Matrigel was added to each well (150 μL) and incubated for 10 min to enable spreading. Next, the plate was placed in an incubator at 37°C for 30 min to solidify the gel. Cells were digested, centrifuged, and resuspended. To each well was added 150 ul of cell suspension, and the cell density was set to 60%. The plates were incubated at 37°C, using 5% CO_2 for 12 h. The endothelial tube size and numbers were analyzed under the microscope and each well was photographed. Any increase or decrease in tube formation in the test compared to control wells (no test molecule added) indicated that the molecule was angiogenic or

anti-angiogenic, respectively.

Luciferase reporter assays

miRNA target clone control vector for pEZX-MT06 (CmiT000001-MT06) and the luciferase reporter plasmids with the 3′ UTR of NR4A3 (HmiT019537) and PCSK9 (HmiT006606) were generated by GeneCopoeia Company (USA). According to the manufacturer's instructions, HPAECs were seeded in 24-well plates at 60% confluency and the vectors were co-transfected with miR-224-5p, miR-361-3p or negative control at 10 nM using a transfection reagent (Invitrogen, Invivofectamine® 3.0). Luciferase activity was measured using the Luc-Pair™ Duo-Luciferase HS Assay Kit (GeneCopoeia) in a Glomax™ luminometer.

The sequences of GABRE and CHM promoter with SOX17 binding site were constructed into the promoter region of pGL3-basic vectors (Promega, USA), respectively. The Ad-SOX17 and constructs were co-transfected into HPAECs in 96-wells plates. After 48 h, the activities of firefly and Renilla were detected using a Dual-Luciferase® Reporter Assay System (E1910, Promega). The constructs containing mutant SOX17 binding site served as the mutant control.

ChIP assay

The 10% formalin was used to fix cells to obtain DNA-protein cross-linking, followed by the cutting of DNA fragments into 200-500 bp via ultrasonic. Cell lysates with indicated fragments were subjected to immunoprecipitation with SOX17 antibody or control IgG antibody (Millipore, Billerica, MA). Then the precipitated DNA fragments were captured by using magnetic beads. The qRT-PCR was employed for quantifying the precipitated DNA.

Pull-down analyses

For DNA pull-down, the 5' biotin-labeled GABRE and CHM promoters and non-labeled promoter probes were conjugated with beads for culturing the protein extracts from cells for 2 h. Proteins were subjected to western blot analysis.

Immunohistochemistry (IHC) analysis

IHC assay was conducted as described previously[6]. The IHC assay kit was purchased from ZSGB-Bio (SP-9000). After dewaxing and rehydration, the sections were placed in

citrate antigen retrieval solution at 95°C for 30 min to retrieve the antigen. Next, the sections were permeabilized with 0.5% Triton-X 100 for 15 min and incubated with 3% H_2O_2 deionized water for 10 min to remove endogenous peroxidase activity. After blocking with goat serum for 1 h, the sections were incubated with primary antibody (Anti-SOX17, Abcam, ab224637, 1:500) overnight at 4°C. Subsequently, the sections were incubated with a biotin-labeled secondary antibody (Beyotime, A0279/A0288) for 1 h at room temperature and further incubated with HRP-labeled streptomycin solution for 1 h at room temperature. The reaction product was visualized using a DAB solution. The Olympus BX-50 microscope was used to obtain images.

Multiple-labeling IF assay

Multiple-labeling IF staining was performed as described previously[6]. After dewaxing and rehydration, the sections were placed in citrate antigen retrieval solution at 95°C for 30 min to retrieve the antigen. Next, the sections were permeabilized with 0.5% Triton-X 100 (Sigma-Aldrich, 9036-19-5) for 15 min and blocked with 3% BSA (Sigma-Aldrich, A1933) for 1 h. Next, the sections were incubated with anti-CD31 antibody (Abcam, ab18291, 1:200) overnight at 4°C and were subsequently incubated with a secondary antibody labeled with Cy3 (Beyotime, P0183) or FITC (Beyotime, P0562) for 1 h at room temperature. The sections were washed using PBS thrice and were incubated with 3% BSA for 1 h. After elution, the sections were incubated with anti-NR4A3 antibody (Proteintech, 55405-1-AP, 1:200) or anti-SOX17 (Abcam, ab224637, 1:400) antibody or anti-Ki67 antibody (Abcam, ab15580, 1:500) overnight at 4°C and subsequently incubated with secondary antibody labeled with Cy5 (Servicebio, GB27301) or FITC (Beyotime, P0562) at room temperature for 1 h. The sections were washed using PBS thrice and incubated with 3% BSA for 1 h. After elution, the sections were incubated with anti-PCSK9 antibody (Proteintech, 27882-1-AP, 1:100) overnight at 4°C and were subsequently incubated with secondary antibody labeled with FITC (Beyotime, P0562) at room temperature for 1 h. After elution using PBS, the sections were subsequently incubated with DAPI to stain the cell nucleus. After elution with PBS, the sections were imaged via Olympus IX71 fluorescence microscopy.

Western blot assays

Western blotting was performed as described previously[64]. Lysis buffer (Beyotime Institute of Biotechnology, China) was used to extract total cellular protein. A BCA concentration assay kit (Beyotime, P0010S) was used to measure the protein concentration in exosomal fractions. Eight μg protein from exosome samples was added into each well of 10% SDS/PAGE gels and subjected to electrophoresis. After blocking with 5% BSA for 1 h, PVDF membranes (Immobilon, cat.IPVH00010) were incubated with primary antibodies including anti-SOX17 (Abcam, ab224637, 1:1000), anti-NR4A3 (Proteintech, 55405-1-AP, 1:1000), anti-PCSK9 (Proteintech, 27882-1-AP, 1:500), anti-NFκB p65 (Abclonal, A2547, 1:500), anti-phospho-NF-kB p65 (Abclonal, AP0124, 1:1000), anti-Caspase3 (Abclonal, A16793, 1:1000), anti-cleaved-Caspase3 (Abclonal, A11021, 1:1000) and anti-PCNA antibodies (Proteintech, 10205-2-AP, 1:3000), anti-cyclinD1 (Proteintech, 26939-1-AP, 1:2000), anti-TNFα (Abclonal, WH235921, 1:1000) overnight at 4°C. The secondary antibodies used were HRP-linked goat anti-rabbit IgG (Beyotime, A0208, 1:2000) and HRP-linked goat anti-mouse IgG (Beyotime, A0216, 1:2000). BeyoECL Plus (Beyotime, P0018M) was used to visualize target bands using the ChemiDoc™ Imager (Bio-Rad). The Image J software was used to analyze the relative intensity of the immunoreactive bands. Density analysis was used to quantify the expression of the target protein relative to β-actin.

NF-κB activity assays

NF-κB p65 Transcription Factor Assay Kit

According to the manufacturer's instructions (Cayman, 10007889), the standard substance was gradient diluted (1500 ng/L, 1000ng/L, 500 ng/L, 250 ng/L, 125 ng/L) and added into the microporous plate for 50 μl per well. Set blank control hole and sample hole to be tested respectively. Add the sample dilution 40μl to the sample well of the elisa coated plate, and then put sample for 10μl. The Elisa coated plate was sealed with sealing plate film and placed into an incubator of 37 °C for 30 min. Dilute the concentrated washing solution 30 times with distilled water. Then the elisa coated plate was wash with above washing solution for 5 times. Beside the blank well, each well was added HRP-conjugate reagent for 50μl. The elisa coated plate was sealed with sealing plate film and placed into an incubator of 37 °C for 30 min, and then wash with washing

solution for 5 times. Then each well was added 50μl chromogenic agents A and B respectively and kept in dark place for 15 min. Finally, each well was add 50 μl stop solution. The absorbance at 450nm was measured with a micro-plate reader.

Nuclear translocation and activation of NFκB p65 in lung tissues

Lung sections were used to perform IF staining. DAPI was used to stain nuclei, and the anti-NFκB p65 antibody (Abcam, ab32536) was used to incubate the sections and label with FITC secondary antibody (Beyotime, P0562) to identify NFκB p65 expression. The nuclear translocation and activity of NFκB p65 were measured using Image J from fluorescence images.

Small RNA sequencing

The miRNA profile in exosomes from HPAECs overexpressing Ad-GFP-Empty and Ad-GFP-SOX17 was analyzed by Ribobio Co. Ltd (Ribobio, China). Exosomes were isolated from the HPAECs supernatant using the RiboTM Exosome Isolation Reagent (Ribobio, China), and exosomal RNA was extracted using the MagZol reagent (Magen, R4801-01) according to the manufacturer's protocol. The quantity and integrity of exosomal RNA yield were assessed using the Qubit®2.0 (Invitrogen, USA) and Agilent 2200 TapeStation (Agilent Technologies, USA). Briefly, RNAs were ligated with a 3′ RNA adapter followed by 5′ adapter ligation. Subsequently, the adapter-ligated RNAs were subjected to RT-PCR and amplified with a low-cycle number. The PCR products were size-selected via PAGE gel electrophoresis according to instructions from the NEBNext® Multiplex Small RNA Library Prep Set for Illumina® (Illumina, USA). The purified library products were evaluated using the Agilent 2200 TapeStation, and the libraries were sequenced using the HiSeq 2500 (Illumina, USA) with single-end 50 bp sequencing at Ribobio Co. Ltd (Ribobio, China). Data analysis: the raw reads were processed by filtering reads with adapters, poly 'N', low quality, smaller than 17 nt using FASTQC to get clean reads. Clean reads were mapped to the reference genome GCF_000001405.25_GRCh37.p13_genomic.gff.chr using bowtie-1.1.1. miRDeep2 was used to identify known mature miRNAs based on data from miRBase (www.miRBase.org) and predict novel miRNA. The databases of Rfam12.1 (https://ftp.ebi.ac.uk/pub/databases/Rfam/12.1/) and pirnabank

(http://pirnabank.ibab.ac.in/) were used to identify rRNA, tRNA, snRNA, snoRNA and piRNA using a BLAST search. The miRNA expression was calculated using RPM (Reads Per Million) values (PRM = (number of reads mapping to miRNA/ number of reads in clean data) $\times 10^6$). The expression levels were normalized by RPM, where RPM is equal to the number of reads that map to miRNA/number of reads in clean data $\times 10^6$. The differential expression between two sets of samples was calculated by the DESeq2 algorithm according to the criteria of ｜log2 (Fold Change)｜≥1 and a P-value < 0.05. KOBAS was used to perform Gene Ontology (GO) and KEGG (Kyoto Encyclopedia of Genes and Genomes) pathway analysis. The original data has been uploaded on the SRA database (BioProject ID: PRJNA781679). The original matadata can be read at https://dataview.ncbi.nlm.nih.gov/object/PRJNA781679?reviewer=qnln9d29u7v0nbed3 5v21f9p6c.

RNA sequencing

RNA sequencing of HPAECs treated with miR-224-5p or miR-361-3p inhibitor with three biological replicates was performed by Ribobio Co. Ltd (Ribobio, China). Total RNA was isolated from cells using the MagZol reagent (Magen, China) according to the manufacturer's protocol, and the quantity and integrity of RNA yield were assessed using the K5500 (Kaiao, China) and the Agilent 2200 Tape Station (Agilent Technologies, USA). Briefly, mRNA was enriched using an oligo-dT based method according to the instructions in the NEBNext® Poly(A) mRNA Magnetic Isolation Module (NEB, USA). The DNA was fragmented to ~200 bp, and the RNA fragments were subjected to first strand and second strand cDNA synthesis followed by adaptor ligation and enrichment at a low PCR cycle number according to the instructions in NEBNext® Ultra™ RNA Library Prep Kit for Illumina. The purified library products were evaluated using the Agilent 2200 Tape Station and Qubit (Thermo Fisher Scientific, USA). The libraries were sequenced on an Illumina platform with paired-end 150 bp obtained at Ribobio Co. Ltd (Ribobio, China). The original data has been uploaded on the SRA database (BioProject ID: PRJNA781655).

Quantification and statistical analysis

All data acquisition and analysis were performed by investigators blinded to the

experimental group. For biochemical analyses, a minimum of three samples per genotype was used for each analysis, whereas *in vivo* analysis included at least six mice per group. These sample sizes are sufficient to determine whether there is a biologically meaningful difference between different genotypes. For *in vitro* studies, a sufficiently large number of cells were analyzed to ensure the identification of biologically meaningful differences using methods from studies cited throughout the paper. Moreover, results obtained *in vitro* were reliably reproduced in at least three independent experiments. All experimental data were expressed as mean ± SEM unless otherwise mentioned. The normality of the variables was tested using the Shapiro-Wilk test. The data from the analysis met the assumptions of the tests and the variance was similar between the experimental groups (if not, the data was not included in the following analysis). Unpaired two-tailed Student's *t*-test was used when comparing two experimental groups, whereas three experimental groups were analyzed using one-way ANOVA followed by Tukey's post hoc test. GraphPad Prism 8.0 was used for calculations and P-values < 0.05 were considered statistically significant.

Reference

1. Luo Y, Teng X, Zhang L, Chen J, Liu Z, Chen X, Zhao S, Yang S, Feng J, Yan X. CD146-HIF-1α hypoxic reprogramming drives vascular remodeling and pulmonary arterial hypertension. *Nat Commun*. 2019;10:3551.
2. Wojciak-Stothard B, Abdul-Salam VB, Lao KH, Tsang H, Irwin DC, Lisk C, Loomis Z, Stenmark KR, Edwards JC, Yuspa SH, et al. Aberrant Chloride Intracellular Channel 4 Expression Contributes to Endothelial Dysfunction in Pulmonary Arterial Hypertension. *Circulation*. 2014;129:1770–1780.
3. Lu A, Zuo C, He Y, Chen G, Piao L, Zhang J, Xiao B, Shen Y, Tang J, Kong D, et al. EP3 receptor deficiency attenuates pulmonary hypertension through suppression of Rho/TGF-β1 signaling. *J Clin Invest*. 2015;125:1228–1242.
4. Jia D, Bai P, Wan N, Liu J, Zhu Q, He Y, Chen G, Wang J, Chen H, Wang C, et al. Niacin Attenuates Pulmonary Hypertension Through H-PGDS in Macrophages. *Circ Res*. 2020;127:1323–1336.
5. Zou X-Z, Gong Z-C, Liu T, He F, Zhu T-T, Li D, Zhang W-F, Jiang J-L, Hu C-P. Involvement of epithelial-mesenchymal transition afforded by activation of LOX-1/ TGF-β1/KLF6 signaling pathway in diabetic pulmonary fibrosis. *Pulmonary Pharmacology & Therapeutics*. 2017;44:70–77.
6. Liu T, Zou X-Z, Huang N, Ge X-Y, Yao M-Z, Liu H, Zhang Z, Hu C-P. Down-regulation of miR-204 attenuates endothelial-mesenchymal transition by enhancing autophagy in hypoxia-induced pulmonary hypertension. *Eur J Pharmacol*. 2019;863:172673.
